# Supplementary material for: Serotonergic regulation of bipolar cell survival in the developing cerebral cortex
Source: Cell Rep. 2022 Jul 5;40(1):111037. doi: 10.1016/j.celrep.2022.111037 (PMC9638000; doi:10.1016/j.celrep.2022.111037)
Supplement: Document S1. Figures S1–S7 and Table S1 [file mmc1.pdf]

Cell Reports, Volume *40*

Supplemental information

Serotonergic regulation of bipolar cell survival

in the developing cerebral cortex

Fong Kuan Wong, Martijn Selten, Claudia Rosés-Novella, Varun Sreenivasan, Noemí Pallas-Bazarra, Eleni Serafeimidou-Pouliou, Alicia Hanusz-Godoy, Fazal Oozeer, Robert Edwards, and Oscar Marín

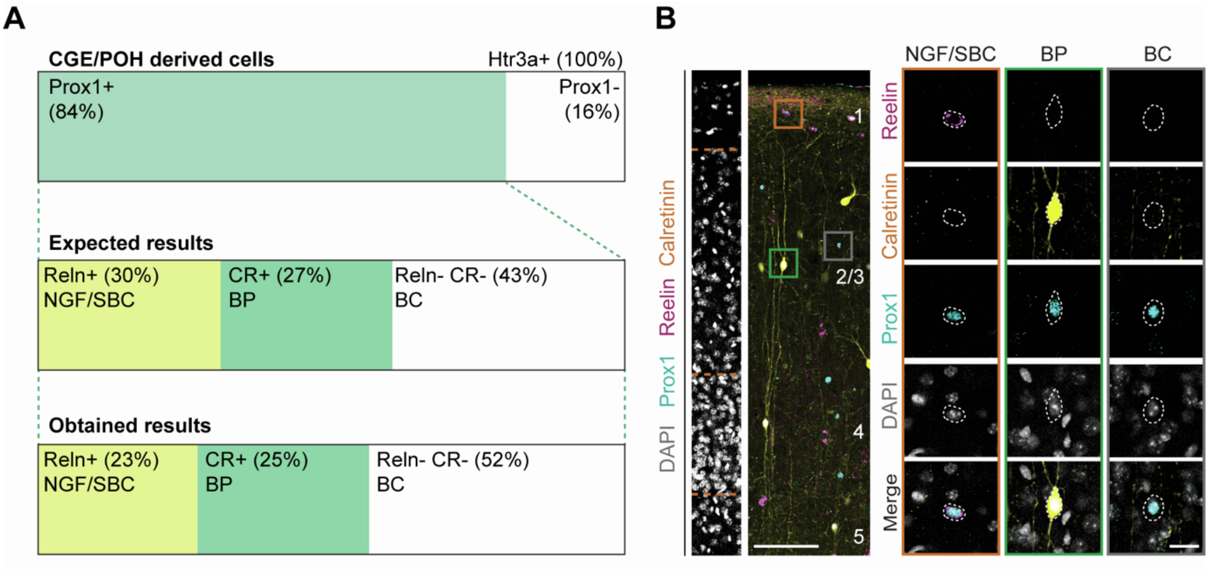

Figure S1. Validation of immunofluorescent strategy related to Figure 1-7

(A) Validation of immunofluorescent strategy. Quantification of percentage of all Htr3a+ interneurons (Prox1+), neurogliaform cells (Prox1+ and Reln+), bipolar cells (Prox1+ and CR+) and CGE-derived basket cells (Prox1+, Reln- and CR-) in wild type mice (n = 5 mice) at P21. Chi-squared test, $p = 0.16$.

(B) Coronal sections through the primary somatosensory cortex of wild type mice at P21 (left) with higher magnifications on the right for neurogliaform/single bouquet cells (yellow box), bipolar cells (green box) and basket cells (grey box) immunostained for Prox1 (cyan), mCherry (red) and calretinin (yellow). DAPI is shown for counterstaining (grey).

Scale bars, 100 μm (left), 20 μm (right).

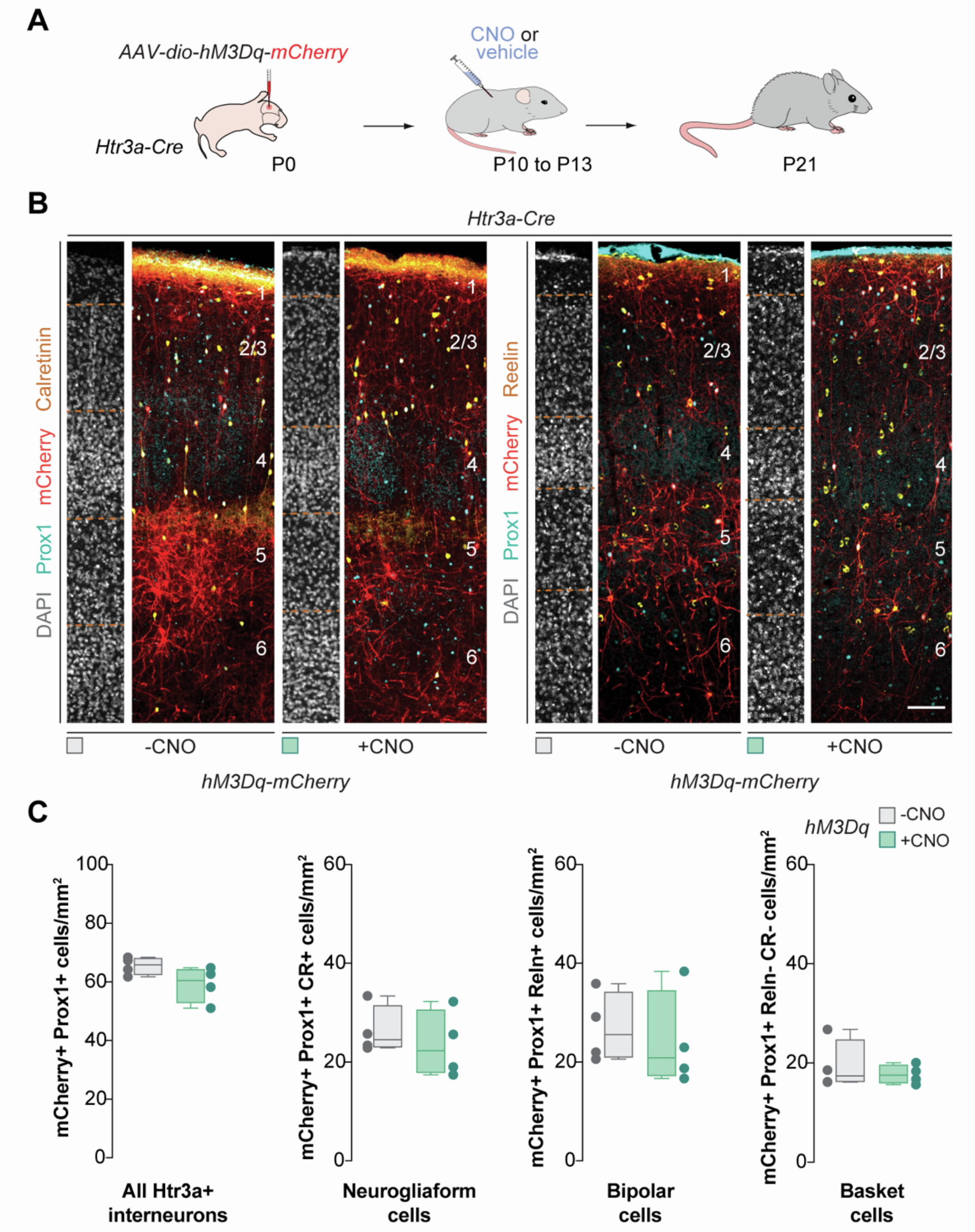

Figure S2. Cell-autonomous changes in neuronal activity beyond the normal period of cell death do not affect the survival of Htr3a+ interneurons related to Figure 3

(A) Schematic of experimental design.

(B) Coronal sections through the primary somatosensory cortex of *Htr3a-Cre* mice at P21 injected with *hM3Dq-mCherry* (left) followed by vehicle or CNO treatment immunostained for Prox1 (cyan), mCherry (red), calretinin (yellow, left) and reelin (yellow, right). DAPI is shown for counterstaining (grey).

(C) Quantification of the density of all transfected Htr3a+ interneurons (mCherry+, Prox1+), neurogliaform cells (mCherry+, Prox1+ and Reln+), bipolar cells (mCherry+, Prox1+ and CR+), and basket cells (mCherry+, Prox1+, Reln- and CR-) in control (grey box plots, $n = 4$ mice) and CNO treated mice injected with *hM3Dq-mCherry* (green box plots, $n = 4$ mice) at P21. Prox1+; two-tailed unpaired Student's t-test, $p = 0.12$. Prox1+ Reln+; two-tailed unpaired Student's t-test, $p = 0.67$. Prox1+ and CR+; two-tailed unpaired Student's t-test, $p = 0.55$. Prox1+, Reln- and CR-; two-tailed unpaired Student's t-test, $p = 0.54$.

Data in panels C are shown as boxplots (median, middle dash), lower and upper quartiles (box borders), and minimum and maximum (whiskers) and the adjacent data points indicate the average cell density in each animal.

Scale bar, 100 μm.

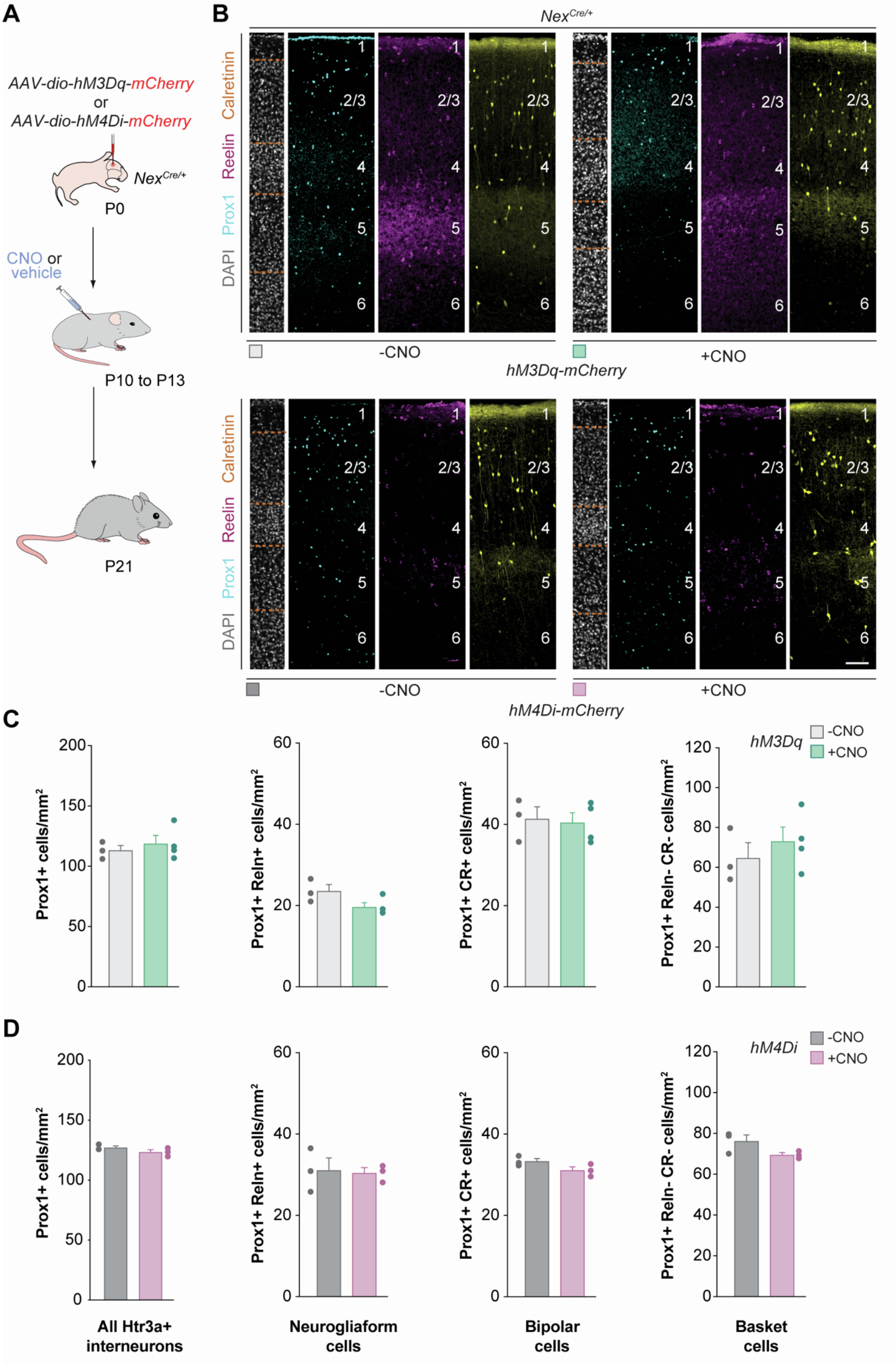
A
AAV-dio-hM3Dq-mCherry
or
AAV-dio-hM4Di-mCherry
Nex Cre/+
P0
CNO or vehicle
P10 to P13
P21
B
Nex Cre/+
DAPI Prox1 Reelin Calretinin
1
2/3
4
5
6
-CNO
+CNO
hM3Dq-mCherry
hM4Di-mCherry
C
Prox1+ cells/mm²
Prox1+ Reln+ cells/mm²
Prox1+ CR+ cells/mm²
Prox1+ Reln- CR- cells/mm²
hM3Dq
-CNO
+CNO
D
hM4Di
-CNO
+CNO
All Htr3a+ interneurons
Neurogliaform cells
Bipolar cells
Basket cells

Figure S3. Non-cell autonomous changes in neuronal activity beyond the normal period of cell death do not affect the survival of Htr3a+ interneuron related to Figure 4

(A) Schematic of experimental design.

(B) Coronal sections through the primary somatosensory cortex of *Nex*$^{Cre/+}$ mice at P21 injected with *hM3Dq-mCherry* virus (top) or *hM4Di-mCherry* (bottom) virus followed by vehicle (left) or CNO (right) treatment immunostained for Prox1 (cyan), reelin (magenta) and calretinin (yellow). DAPI is shown for counterstaining (grey).

(C) Quantification of the density of all Htr3a+ interneurons (Prox1+), neurogliaform cells (Prox1+ and Reln+), bipolar cells (Prox1+ and CR+), and basket cells (Prox1+, Reln- and CR-) in control (grey box plots, $n = 3$ mice) and CNO treated mice injected with hM3Dq-mCherry (green box plots, $n = 4$ mice) at P21. Prox1+; two-tailed unpaired Student's t-test, $p = 0.55$. Prox1+ Reln+; two-tailed unpaired Student's t-test, $p = 0.09$. Prox1+ and CR+; two-tailed unpaired Student's t-test, $p = 0.82$. Prox1+, Reln- and CR-; two-tailed unpaired Student's t-test, $p = 0.47$.

(D) Quantification of the density of all Htr3a+ interneurons (Prox1+), neurogliaform cells (Prox1+ and Reln+), bipolar cells (Prox1+ and CR+), and basket cells (Prox1+, Reln- and CR-) in control (grey box plots, $n = 3$ mice) and CNO treated mice injected with *hM4Di-mCherry* (red box plots, $n = 3$ mice) at P21. Prox1+; two-tailed unpaired Student's t-test, $p = 0.20$. Prox1+ Reln+; two-tailed unpaired Student's t-test, $p = 0.85$. Prox1+ and CR+; two-tailed unpaired Student's t-test, $p = 0.12$. Prox1+, Reln- and CR-; two-tailed unpaired Student's t-test, $p = 0.10$.

Data in panels C and D are shown as bar graphs + SEM, and the adjacent data points indicate the average cell density in each animal.

Scale bar, 100 μm.

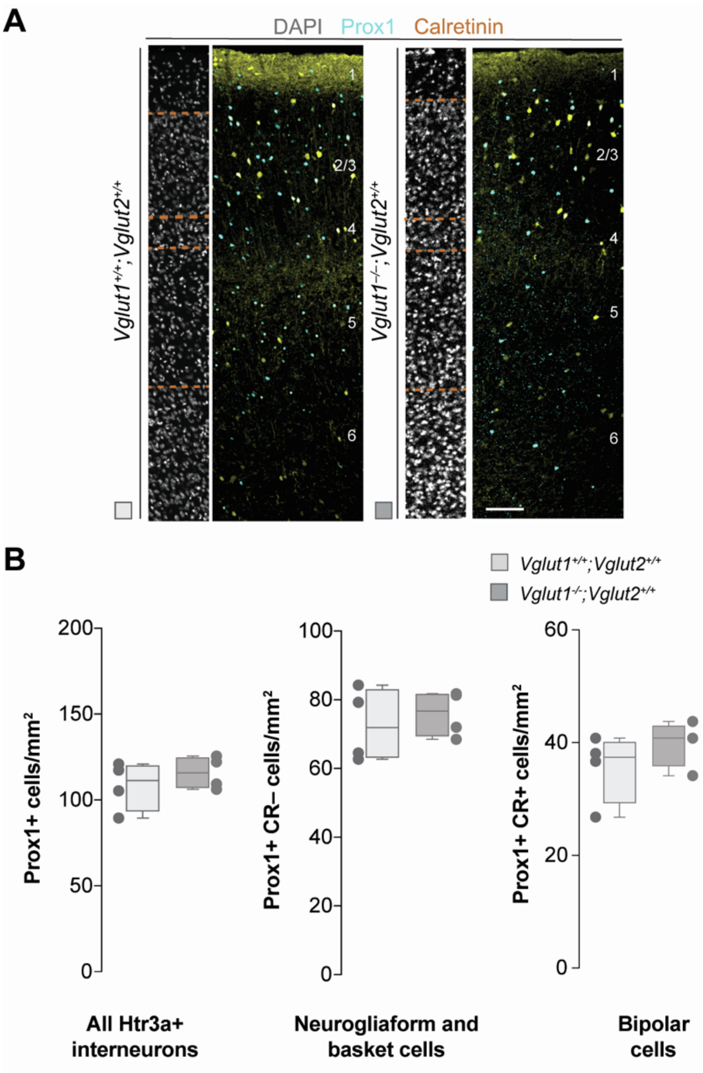

Figure S4. Removal of Vglut1 is not sufficient to alter the survival of Htr3a+ interneurons related to Figure 5

(A) Coronal sections through the primary motor cortex of *Vglut1*$^{+/+}$;*Vglut2*$^{+/+}$ mice ipsilaterally injected with Cre-expressing virus (left) and of the contralateral cortex of *Vglut1*$^{-/-}$;*Vglut2*$^{fl/fl}$ mice injected with Cre-expressing virus (right) at P21 following immunohistochemistry against Prox1 (cyan) and calretinin (yellow). DAPI is shown for counterstaining (grey).

(B) Quantification of the density of all Htr3a+ interneurons (Prox1+), neurogliaform and basket cells (Prox1+ and CR-) and bipolar cells (Prox1+ and CR+) in *Vglut1*$^{+/+}$;*Vglut2*$^{+/+}$ injected with Cre-expressing virus (light grey box plots, n = 4 mice) and *Vglut1*$^{-/-}$;*Vglut2*$^{fl/fl}$ mice injected with Cre-expressing virus at the contralateral (dark grey box plots, n = 4 mice) at P21. Prox1+; two-tailed unpaired Student's t-test, p = 0.41. Prox1+ CR-; two-tailed unpaired Student's t-test, p = 0.63. Prox1+ and CR+; two-tailed unpaired Student's t-test, p = 0.29.

Data in panel B are shown as boxplots (median, middle dash), lower and upper quartiles (box borders), and minimum and maximum (whiskers) and the adjacent data points indicate the average cell density in each animal. Scale bar, 100 μm.

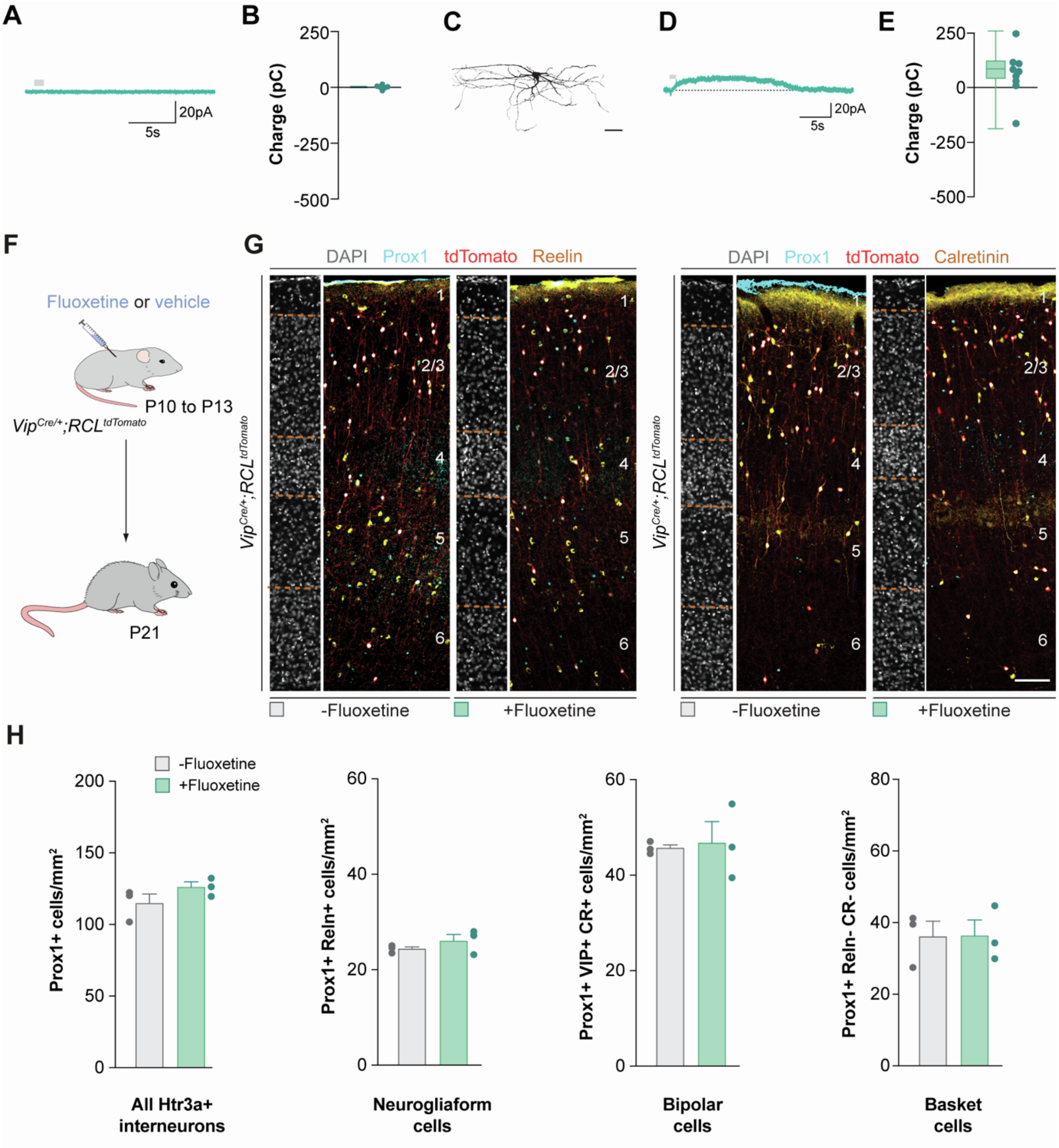

Figure S5. Neurogliaform cells do not depolarize in response to serotonin, and changes in serotonin levels beyond the normal period of cell death do not affect the survival of bipolar cells related to Figure 6

(A) Example of voltage-clamp traces of L2/3 GFP+ cells in response to aCSF puffs (grey bars).

(B) Quantification of cell charge of L2/3 GFP+ (green box plot, $n = 4$ cells).

(C) Typical morphology of reconstructed neurogliaform cell.

(D) Example of voltage-clamp traces of neurogliaform in response to serotonin puffs (grey bars).

(E) Quantification of cell charge of L1 neurogliaform cell (green box plot, $n = 9$ cells).

(F) Schematic of experimental design.

(G) Coronal sections through the primary somatosensory cortex of *Vip*$^{Cre/+}$;*RCL*^{tdTomato} mice at P21 injected vehicle or fluoxetine treatment immunostained for Prox1 (cyan), tdTomato (red) and reelin (yellow, left) or calretinin (yellow, right). DAPI is shown for counterstaining (grey).

(H) Quantification of the density of all Htr3a+ interneurons (Prox1+), neurogliaform (Prox1+ and Reln+), bipolar cells (Prox1+, VIP+ and CR+) and basket cells (Prox1+ Reln- and CR-) in control (grey bars, n = 3 mice) and fluoxetine injected mice (green box bars, n = 3 mice) at P21. Prox1+; two-tailed unpaired Student's t-test, p = 0.21. Prox1+ and Reln+; two-tailed unpaired Student's t-test, p = 0.30. Prox1+, VIP+ and CR+: two-tailed unpaired Student's t-test: p = 0.43. Prox1+, CR- and Reln-; two-tailed unpaired Student's t-test, p = 0.97.

Data in panels B and E are shown as boxplots (median, middle dash), lower and upper quartiles (box borders), and minimum and maximum (whiskers), and the adjacent data points indicate the cell charge for each cell. Data in panel H are shown as bar graphs + SEM, and the adjacent data points indicate the average cell density in each animal.

Scale bar, 100 μm.

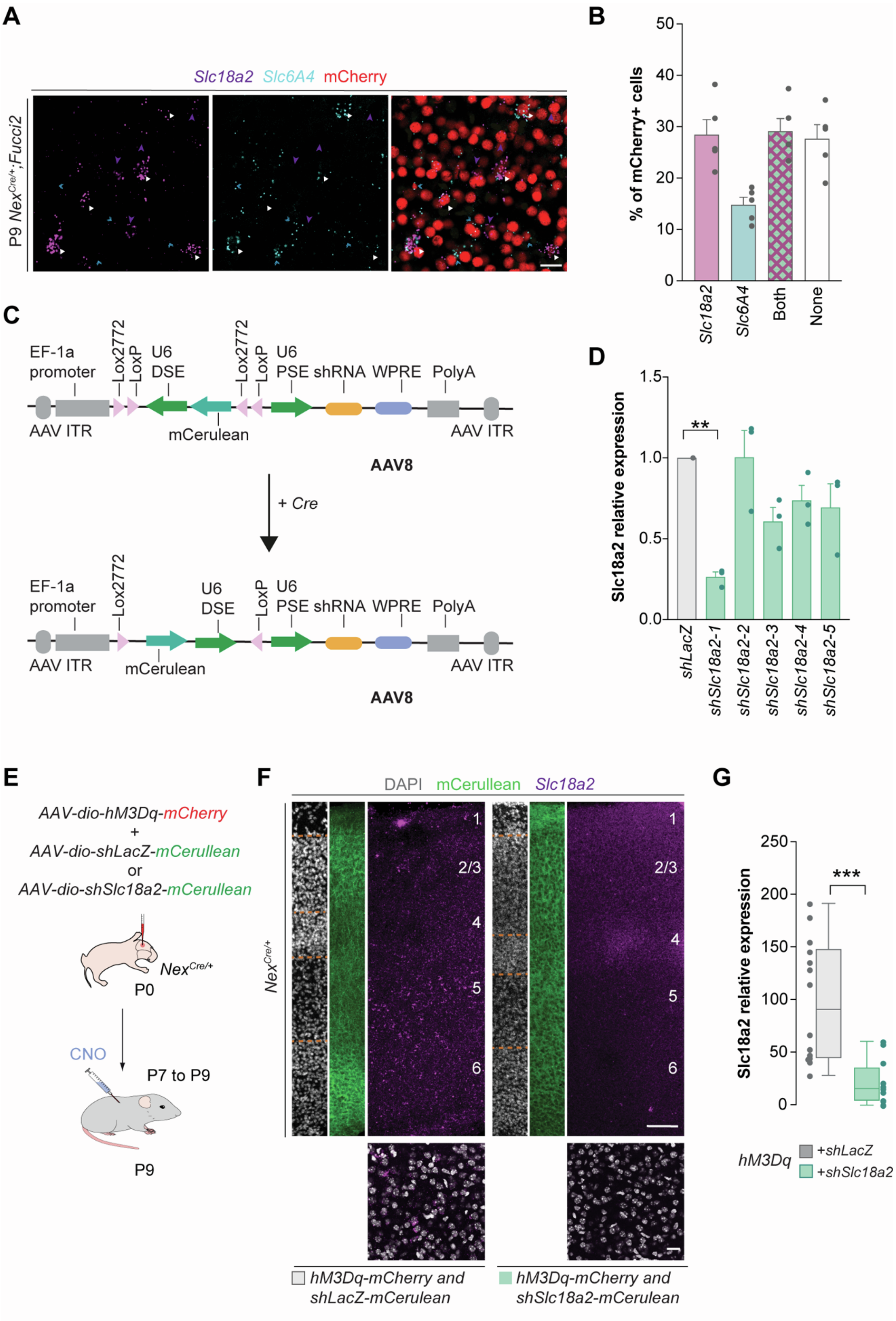

Figure S6. Validation of shSlc18a2-mCerullean construct related to Figure 7

(A) Coronal sections through the infragranular layers of the neocortex of *Nex*$^{Cre/+}$*; Fucci2* mice at P9 immunostained for mCherry (red), and RNAscope for *Slc18a2* (magenta) and *Slc6A4* (cyan). White arrowheads

are examples of pyramidal cells expressing both *Slc18a2* and *Slc6a4*, magenta arrowheads for *Slc18a2* only and cyan arrowheads for *Slc6a4* only.

(B) Quantification of mCherry+ cells expressing *Slc18a2* (magenta bar, n = 5 mice), *Slc6A4* (cyan bar, n = 5 mice), both *Slc18a2* and *Slc6a4* (checkered bar, n = 5 mice) or none of these genes (white bar, n = 5 mice).

(C) Schematic of plasmid design.

(D) Quantification of *Slc18a2* relative expression in vitro using qPCR with control (shLacZ, grey bar, n = 3 biological replicates) and sh18a2 constructs 1 to 5 (green bars, n = 3 biological replicates). *shSlc18a2-1*: one-way ANOVA with Dunnett's multiple comparison, **p = 0.0015; *shSlc18a2-2*: one-way ANOVA with Dunnett's multiple comparison, p = 0.99; *shSlc18a2-3*: one-way ANOVA with Dunnett's multiple comparison, p = 0.08; *shSlc18a2-4*: one-way ANOVA with Dunnett's multiple comparison, p = 0.32; *shSlc18a2-5*: one-way ANOVA with Dunnett's multiple comparison, p = 0.21. *shSlc18a2-1* was selected for in vivo experiments.

(E) Schematic of experimental design.

(F) Coronal sections through the primary somatosensory cortex of $Nex^{Cre/+}$ mice at P9 injected with *hM3Dq-mCherry* and *shLacZ-mCerulean* (left) or *shSlc18a2-mCerrulean* (right) virus followed by CNO treatment immunostained for mCerulean (green), and RNAscope for *Slc18a2* (magenta). DAPI is shown for counterstaining (grey). The insert below is a higher magnification of L5 of the RNAscope for *Slc18a2* (magenta) and DAPI counterstain.

(G) Quantification of the relative expression of *Slc18a2* of L5 and 6 with control (shLacZ, grey box plot, n =14 images) and sh18a2 constructs (green box plot, n =12 images). Mann Whitney test, ***p < 0.0001.

Data in panels B and D are shown as bar graphs ± SEM. The adjacent data points indicate the average cell density in each animal (B) and average expression from each biological replicate. Data in panel G are shown as boxplots (median, middle dash), lower and upper quartiles (box borders), and minimum and maximum (whiskers). The adjacent data points indicate the number of images.

Scale bars, 20 μm (A) and 100 μm (F).

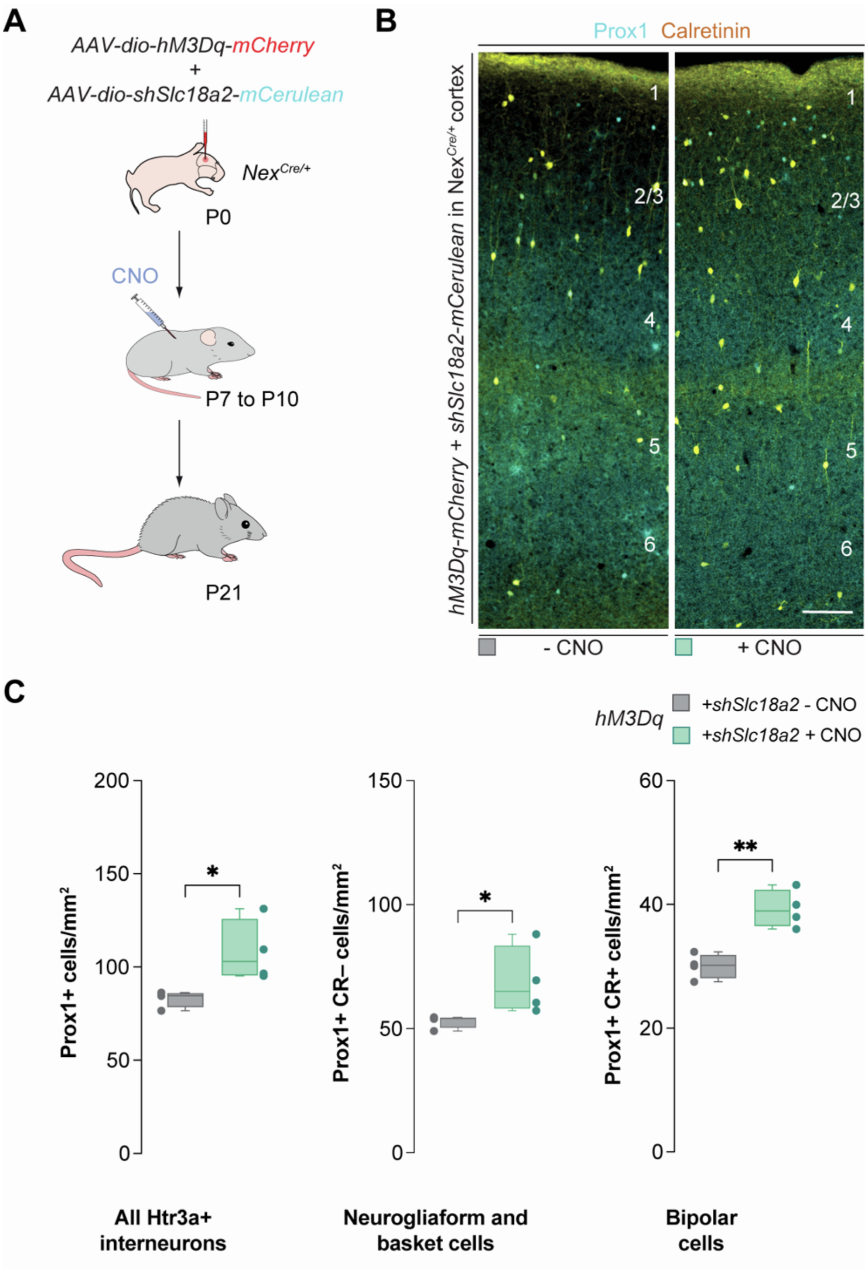

Figure S7. Knockdown of Slc18a2 alone is not sufficient to prevent the cell death of Htr3a+ interneurons related to Figure 7

(A) Schematic of experimental design.

(B) Coronal sections through the primary somatosensory cortex of $Nex^{Cre/+}$ mice at P21 injected with *hM3Dq-mCherry* and *shSlc18a2-mCerrulean* virus followed by vehicle (left) or CNO (right) treatment immunostained for Prox1 (cyan), and calretinin (yellow).

(C) Quantification of the density of all Htr3a+ interneurons (Prox1+), neurogliaform and basket cells (Prox1+ and CR-) and bipolar cells (Prox1+ and CR+) in *shSlc18a2-mCerrulean* mice followed by vehicle (grey box plots, n = 4 mice) or CNO treatment (green box plots, n = 4 mice) at P21. All mice were injected with the *hM3Dq-mCherry* virus. Prox1+; two-tailed unpaired Student's t-test, *$p = 0.027$. Prox1+ CR-; Mann-Whitney, *$p = 0.029$. Prox1+ and CR+; two-tailed unpaired Student's t-test, **$p = 0.0023$.

Data in panel C are shown as boxplots (median, middle dash), lower and upper quartiles (box borders), and minimum and maximum (whiskers) and the adjacent data points indicate the average cell density in each animal. Scale bar, 100 μm.

Table S1. Summary of data and statistical analyses, related to Figures 1-7 and Figures S1-S7

FIGURE 1	Measurement	Values	N	Statistical	P value
Figure 1B	CGE interneuron cell densities (mean ± SEM)	**Prox1+**: Control Htr3a-Cre: 128.93891 ± 9.7075; Htr3aCreBaxBak: 170.999 ± 7.6148	[brains] Control Htr3a-Cre, n = 5, Htr3aCreBaxBak, n = 4	2-tailed unpaired Student's t-test	p = 0.0138 (*)
		**Prox1+ Re+**: Control Htr3a-Cre: 30.018342 ± 2.4084662; Htr3aCreBaxBak: 39.798868 ± 2.812634	[brains] Control Htr3a-Cre, n = 5, Htr3aCreBaxBak, n = 4	2-tailed unpaired Student's t-test	p = 0.0327 (*)
		**Prox1+ CR+**: Control Htr3a-Cre: 33.222357 ± 2.5819982; Htr3aCreBaxBak:44.76981 ± 3.1535676	[brains] Control Htr3a-Cre, n = 5, Htr3aCreBaxBak, n = 4	2-tailed unpaired Student's t-test	p = 0.0309 (*)
		**Prox1+ CR-Re-**: Control Htr3a-Cre: 65.698208 ± 6.7524041; Htr3aCreBaxBak: 86.430708 ± 3.031828	[brains] Control Htr3a-Cre, n = 5, Htr3aCreBaxBak, n = 4	2-tailed unpaired Student's t-test	p = 0.0327 (*)
**FIGURE 2**	Measurement	Values	N	Statistical	P value
Figure 2B	CGE interneuron cell densities (mean ± SEM)	**Prox1+**: hM3Dq-CNO: 63.134157 ±2.855373; hM3Dq+CNO: 77.079124 ± 2.998725	[brains] n = 6 for both groups	2-tailed unpaired Student's t-test	p = 0.007 (**)
		**Prox1+ Re+**: hM3Dq-CNO: 18.0878276 ± 1.22917991; hM3Dq+CNO: 25.807598± 2.9800354	[brains] n = 6 for both groups	2-tailed unpaired Student's t-test	p = 0.0377 (*)
		**Prox1+ CR+**: hM3Dq-CNO: 23.053624 ±1.6097517; hM3Dq+CNO: 31.14537 ± 1.8146412	[brains] n = 6 for both groups	2-tailed unpaired Student's t-test	p = 0.0121 (*)
		**Prox1+ CR-Re-**: hM3Dq-CNO: 27.4930811 ± 3.02664295; hM3Dq+CNO: 37.6835104 ± 2.75023481	[brains] n = 6 for both groups	2-tailed unpaired Student's t-test	p = 0.0319 (*)
Figure 2C	CGE interneuron cell densities (mean ± SEM)	**Prox1+**: hM4Di-CNO: 68.915974 ±5.2489537; hM4Di+CNO: 55.0325501 ± 1.96627922	[brains] hM4Di -CNO, n = 4, hM4Di+CNO n = 5	2-tailed unpaired Student's t-test	p = 0.0259 (*)
		**Prox1+ Re+**: hM4Di-CNO: 24.9941011 ±1.1484826; hM4Di+CNO: 17.5705351 ± 1.39640632	[brains] hM4Di -CNO, n = 4, hM4Di+CNO n = 5	2-tailed unpaired Student's t-test	p = 0.0055 (**)
		**Prox1+ CR+**: hM4Di-CNO: 30.0366435 ± 2.11197677; hM4Di+CNO: 21.4524183 ± 1.13356151	[brains] hM4Di -CNO, n = 4, hM4Di+CNO n = 5	2-tailed unpaired Student's t-test	p = 0.0047 (**)
		**Prox1+ CR-Re-**: hM4Di-CNO: 20.8646658 ± 0.74526041; hM4Di+CNO: 12.2050725 ± 0.83210095	[brains] hM4Di -CNO, n = 4, hM4Di+CNO n = 5	2-tailed unpaired Student's t-test	p = 0.0001 (***)
**FIGURE 3**	Measurement	Values	N	Statistical	P value
Figure 3B	CGE interneuron cell densities (mean ± SEM)	**Prox1+**: Control Nex-Cre:125.955086 ±4.9773057; NexCreBaxBak: 171.70553 ±8.352957	[brains] n = 7 for both groups	2-tailed unpaired Student's t-test	p = 0.0005 (***)
		**Prox1+ Re+**: Control Nex-Cre: 29.690536 ± 1.9780853; NexCreBaxBak: 38.699654 ± 2.2600626	[brains] n = 7 for both groups	2-tailed unpaired Student's t-test	p = 0.0113 (*)
		**Prox1+ CR+**: Control Nex-Cre: 29.707267 ± 1.3975519; NexCreBaxBak: 42.090428 ± 3.091244	[brains] n = 7 for both groups	2-tailed unpaired Student's t-test	p = 0.0036 (**)
		**Prox1+ CR-Re-**: Control Nex-Cre: 66.553056 ± 3.38164542; NexCreBaxBak: 90.979209 ± 4.2374106	[brains] n = 7 for both groups	2-tailed unpaired Student's t-test	p = 0.002 (**)
**FIGURE 4**	Measurement	Values	N	Statistical	P value
Figure 4C	CGE interneuron cell densities (mean ± SEM)	**Prox1+**: hM3Dq-CNO: 140.22185 ±6.1886976; hM3Dq+CNO: 168.24575 ±9.2067176	[brains] n = 7 for both groups	2-tailed unpaired Student's t-test	p = 0.0266 (*)
		**Prox1+ Re+**: hM3Dq-CNO: 30.5171011 ± 1.28699855; hM3Dq+CNO: 40.5280454 ± 1.0966376	[brains] n = 7 for both groups	2-tailed unpaired Student's t-test	p < 0.0001 (***)
		**Prox1+ CR+**: hM3Dq-CNO: 41,618613 ± 1.9027866; hM3Dq+CNO: 51.031663 ± 1.7405535	[brains] n = 7 for both groups	2-tailed unpaired Student's t-test	p = 0.0033 (**)
		**Prox1+ CR-Re-**: hM3Dq-CNO: 42.1597898 ± 2.2139241; hM3Dq+CNO: 60.3700123 ± 3.03048226	[brains] n = 7 for both groups	2-tailed unpaired Student's t-test	p = 0.0004 (***)

Figure 4D	CGE interneuron cell densities (mean ± SEM)	**Prox1+**: hM4Di-CNO: 175.759708 ± 6.2889318; hM4Di+CNO:153.962902 ± 6.37779553	[brains] n = 6 for both groups	2-tailed unpaired Student's t-test	*p* = 0.0352 (*)
		**Prox1+ Re+**: hM4Di-CNO: 34.9688292 ± 1.89106344; hM4Di+CNO: 26.9684615 ± 1.43152338	[brains] hM4Di -CNO, n = 5, hM4Di+CNO n = 6	2-tailed unpaired Student's t-test	*p* = 0.0074 (**)
		**Prox1+ CR+**: hM4Di-CNO: 50.7213356 ± 1.6083140; hM4Di+CNO: 40.5899939 ± 3.37004675	[brains] n = 6 for both groups	2-tailed unpaired Student's t-test	*p* = 0.0218 (*)
		**Prox1+ CR-Re-**: hM4Di-CNO: 76.786516 ± 4.023283; hM4Di+CNO: 58.709725 ± 5.5024846	[brains] n = 6 for both groups	2-tailed unpaired Student's t-test	*p* = 0.0242 (*)
**FIGURE 5**	Measurement	Values	N	Statistical	P value
Figure 5D	CGE interneuron cell densities (mean ± SEM)	**Prox1+**: Control: 108.282796 ± 7.10449118; VG1/VG2 KO: 90.2908768 ± 5.94786571	[brains] n = 4 for both groups	2-tailed unpaired Student's t-test	*p* = 0.1002 (NS)
		**Prox1+ CR-**: Control: 72.6899503 ± 5.35347727; VG1/VG2 KO: 54.7620478 ± 2.43474891	[brains] n = 4 for both groups	2-tailed unpaired Student's t-test	*p* = 0.026 (*)
		**Prox1+ CR+**: Control: 35.5928459 ± 3.05633804; VG1/VG2 KO: 35.528829 ± 3.99582597	[brains] n = 4 for both groups	2-tailed unpaired Student's t-test	*p* = 0.9903 (NS)
**FIGURE 6**	Measurement	Values	N	Statistical	P value
Figure 6C	Charge (mean ± SEM)	L2/3 Bipolar cells: 76704.81 ± 29550.95	[cells] n = 7		
		L2/3 Basket cells: -229518 ± 51407.16	[cells] n = 7		
Figure 6D	CGE interneuron cell densities (mean ± SEM)	**Prox1+**: Vehicle: 140.167528 ± 5.97875389; Fluoxetine: 146.833266 ± 10.612415	[brains] n = 4 for both groups	2-tailed unpaired Student's t-test	*p* = 0.6093 (NS)
		**Prox1+ Re+**: Vehicle: 145.348521 ± 5.79093263; Fluoxetine: 151.235858 ± 18.8224061	[brains] n = 4 for both groups	2-tailed unpaired Student's t-test	*p* = 0.9834 (NS)
		**Prox1+ VIP+ CR+**: Vehicle: 36.6817236 ± 1.65394208; Fluoxetine: 49.087944 ± 3.12794821	[brains] n = 4 for both groups	2-tailed unpaired Student's t-test	*p* = 0.0127 (*)
		**Prox1+ CR-Re-**: Vehicle: 55.493346 ± 8.7814519; Fluoxetine: 41.861755 ± 5.0760216	[brains] n = 4 for both groups	2-tailed unpaired Student's t-test	*p* = 0.2276 (NS)
**FIGURE 7**	Measurement	Values	N	Statistical	P value
Figure 7C	CGE interneuron cell densities (mean ± SEM)	**Prox1+**: shLacZ: 117.845708 ± 7.74735528; shVmat2: 111.997696 ± 8.354898	[brains] n = 4 for both groups	2-tailed unpaired Student's t-test	*p* = 0.5347 (NS)
		**Prox1+ CR-**: shLacZ: 72.9697875 ± 7.84245371; shVmat2: 71.6411254 ± 6.92400581	[brains] n = 4 for both groups	2-tailed unpaired Student's t-test	*p* = 0.8547 (NS)
		**Prox1+ CR+**: shLacZ: 44.8759186 ± 1.32738498; shVmat2: 39.2727969 ± 1.52053381	[brains] n = 4 for both groups	2-tailed unpaired Student's t-test	*p* = 0.0343 (*)
**FIGURE S1**	Measurement	Values	N	Statistical	P value
Figure S1A	Proportion of cells (%)	**Expected results**: NGF/SBC: 30%; BP: 27%; BC: 43%; **Observed results**: NGF/SBC: 23%; BP: 25%; BC 52%	[brains] Control Htr3a-Cre, n = 5, Htr3aCreBaxBak, n = 4	Chi-quare	*p* = 0.16 (NS)
**FIGURE S2**	Measurement	Values	N	Statistical	P value
Figure S2C	CGE interneuron cell densities (mean ± SEM)	**Prox1+**: hM3Dq-CNO: 66.284849 ± 1.5028887; hM3Dq+CNO: 60.064579 ±3.047367	[brains] n = 4 for both groups	2-tailed unpaired Student's t-test	*p* = 0.1169 (NS)
		**Prox1+ Re+**: hM3Dq-CNO: 26.8957068 ± 3.53002062; hM3Dq+CNO: 25.1264708 ± 4.90953428	[brains] n = 4 for both groups	2-tailed unpaired Student's t-test	*p* = 0.6692 (NS)
		**Prox1+ CR+**: hM3Dq-CNO: 26.3253613 ± 2.43294121; hM3Dq+CNO: 23.5634017 ± 3.39731554	[brains] n = 4 for both groups	2-tailed unpaired Student's t-test	*p* = 0.5532 (NS)
		**Prox1+ CR-Re-**: hM3Dq-CNO: 19.42593 ± 2.509301; hM3Dq+CNO: 18.053787 ± 0.9675375	[brains] n = 4 for both groups	2-tailed unpaired Student's t-test	*p* = 0.5436 (NS)
**FIGURE S3**	Measurement	Values	N	Statistical	P value
		**Prox1+**: hM3Dq-CNO: 113.063459 ±4.09645928; hM3Dq+CNO: 118.699518 ± 6.80751163	[brains] hM3Dq -CNO, n = 3, hM3Dq+CNO n = 4	2-tailed unpaired Student's t-test	*p* = 0.5480 (NS)

Figure S3C	CGE interneuron cell densities (mean ± SEM)	**Prox1+ Re+**: hM3Dq-CNO: 23.5569756 ± 1.62279407; hM3Dq+CNO: 19.612295 ± 1.10857406	[brains] hM3Dq -CNO, n = 3, hM3Dq+CNO n = 4	2-tailed unpaired Student's t-test	*p* = 0.0910 (NS)
		**Prox1+ CR+**: hM3Dq-CNO: 41.360781 ± 2.99063331; hM3Dq+CNO: 40.424176 ± 2.46681341	[brains] hM3Dq -CNO, n = 3, hM3Dq+CNO n = 4	2-tailed unpaired Student's t-test	*p* = 0.8172 (NS)
		**Prox1+ CR-Re-**: hM3Dq-CNO: 64.6671703 ± 7.73374349; hM3Dq+CNO: 73.0327285 ± 7.24395448	[brains] hM3Dq -CNO, n = 3, hM3Dq+CNO n = 4	2-tailed unpaired Student's t-test	*p* = 0.4712 (NS)
Figure S3D	CGE interneuron cell densities (mean ± SEM)	**Prox1+**: hM4Di-CNO: 127.116395 ± 1.37724603; hM4Di+CNO: 123.338106 ± 2.02843504	[brains] n = 3 for both groups	2-tailed unpaired Student's t-test	*p* = 0.1982 (NS)
		**Prox1+ Re+**: hM4Di-CNO: 31.0610279 ± 3.09209328; hM4Di+CNO: 30.3948183 ± 1.20604652	[brains] n = 3 for both groups	2-tailed unpaired Student's t-test	*p* = 0.8507 (NS)
		**Prox1+ CR+**: hM4Di-CNO: 33.2822563 ± 0.71278089; hM4Di+CNO: 31.0766782 ± 0.1240153	[brains] n = 3 for both groups	2-tailed unpaired Student's t-test	*p* = 0.1240 (NS)
		**Prox1+ CR-Re-**: hM4Di-CNO: 76.1695 ± 3.079432; hM4Di+CNO: 69.42585 ± 1.046768	[brains] n = 3 for both groups	2-tailed unpaired Student's t-test	*p* = 0.1068 (NS)
**Figure S4**	Measurement	Values	N	Statistical	P value
Figure S4B	CGE interneuron cell densities (mean ± SEM)	**Prox1+**: *Vglut1+/+;Vglut2+/+* : 108.282796 ± 7.10449118 *Vglut1-/-;Vglut2+/+* : 115.77086 ± 4.7356754	[brains] n = 4 for all groups	2-tailed unpaired Student's t-test	p = 0.4142 (NS)
		**Prox1+ CR-**: Vglut1+/+;Vglut2+/+: 72.6899503 ± 5.35347727; Vglut1-/-;Vglut2+/+: 75.899582 ± 3.3346367	[brains] n = 4 for all groups	2-tailed unpaired Student's t-test	p = 0.6290 (NS)
		**Prox1+ CR+**: Vglut1+/+;Vglut2+/+: 35.5928459 ± 3.05633804; Vglut1-/-;Vglut+/+: 39.871273 ± 2.0342391	[brains] n = 4 for all groups	2-tailed unpaired Student's t-test	p = 0.2881 (NS)
**FIGURE S5**	Measurement	Values	N	Statistical	P value
Figure S5A	Charge (mean ± SEM)	GFP+ cells: 971.4932 ± 4764.166	[cells] n = 6		
Figure S5B	Charge (mean ± SEM)	L1 NGF: 62304.41 ± 36233.81	[cells] n = 9		
Figure S5H	CGE interneuron cell densities (mean ± SEM)	**Prox1+**: Vehicle: 114.782539 ± 6.54602903; Fluoxetine: 126.079933 ±3.68675634	[brains] n = 3 for both groups	2-tailed unpaired Student's t-test	*p* = 0.2071 (NS)
		**Prox1+ Re+**: Vehicle: 24.3711865 ±0.40454126; Fluoxetine: 26.0408337 ±1.32701948	[brains] n = 3 for both groups	2-tailed unpaired Student's t-test	*p* = 0.2951 (NS)
		**Prox1+ VIP+ CR+**: Vehicle: 49.0143131 ± 0.79261738; Fluoxetine: 50.1843306 ± 4.20798866	[brains] n = 3 for both groups	2-tailed unpaired Student's t-test	*p* = 0.4295 (NS)
		**Prox1+ CR-Re-**: Vehicle: 36.062905 ± 4.33895612; Fluoxetine: 36.3178117 ± 4.38526967	[brains] n = 3 for both groups	2-tailed unpaired Student's t-test	*p* = 0.9694 (NS)
**FIGURE S6**	Measurement	Values	N	Statistical	P value
Figure S6B	% of mCherry+ cells (mean ± SEM)	Slc18a+ only cells: 28.42467 ± 2.9634; Slc6A4+ only cells: 13.81044 ± 1.4421; Slc18a+ and Slc6A4+ cells: 29.11470 ± 2.46729; Slc18a0- and Slc6A4- cells: 27.65019 ± 2.7433	[brains] n = 5 for all groups		
Figure S6D	Slc18a2 relative expression (mean ± SEM)	shslc18a2-1: 026333 ± 0.03179797; shslc18a2-2: 1.003333 ± 0.16676664; shslc18a2-3: 0.6066667 ± 0.08819171; shslc18a2-4: 0.7366667 ± 0.09333333; shslc18a2-5: 06933333 ± 0.14678026	[experiments] n = 3 for all groups	1-way Anova	*p* = 0.0015 (**) shLacZ vs *shSlc18a2-1*
Figure S6G	Slc18a2 relative expression (mean ± SEM)	shLacZ: 100 ± 15.3447603, shslc18a2: 22.4988438 ± 6.0643048	[images] shLacZ = 14, shslc18a1 = 12	Mann Whitney	*p* < 0.0001 (***)
**FIGURE S7**	Measurement	Values	N	Statistical	P value
Figure S7C	CGE interneuron cell densities (mean ± SEM)	**Prox1+:** -CNO: 83.01319 ± 2.18582;+CNO: 111.997696 ± 8.354898	[brains] n = 4 for both groups	2-tailed unpaired Student's t-test	*p* = 0.027 (*)
		**Prox1+ CR-** : -CNO: 52.97631 ± 1.30069; +CNO: 71.6411254 ± 6.92400581	[brains] n = 4 for both groups	Mann Whitney	*p* = 0.0286 (*)
		**Prox1+ CR+** : -CNO: 30.03689 ± 0.9959; +CNO: 39.2727969 ± 1.52053381	[brains] n = 4 for both groups	2-tailed unpaired Student's t-test	*p* = 0.0023 (**)
