# Supplementary material for: Serotonergic regulation of bipolar cell survival in the developing cerebral cortex
Source: Cell Rep. 2022 Jul 5;40(1):111037. doi: 10.1016/j.celrep.2022.111037 (PMC9638000; doi:10.1016/j.celrep.2022.111037)
Supplement: Document S2. Article plus supplemental information [file mmc2.pdf]

Cell Reports

Article

Serotonergic regulation of bipolar cell survival in the developing cerebral cortex

Graphical abstract

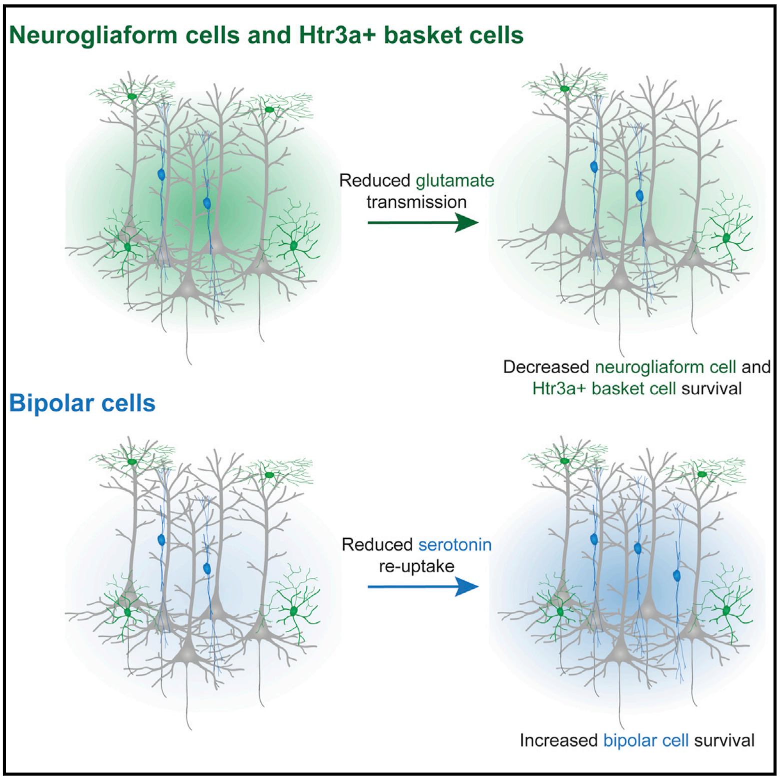

Highlights

- Htr3a+ interneurons undergo activity-dependent programmed cell death
- The survival of Htr3a+ interneurons is regulated in an input-specific manner
- The survival of neurogliaform and basket cells depend on glutamatergic inputs
- The survival of bipolar cells depends on serotonergic inputs

Authors

Fong Kuan Wong, Martijn Selten, Claudia Rosés-Novella, ..., Fazal Oozeer, Robert Edwards, Oscar Marín

Correspondence

oscar.marin@kcl.ac.uk

In brief

Wong et al. demonstrate that the number of Htr3a+ interneurons in mice is regulated in an input-specific manner during the period of programmed cell death. The survival of neurogliaform and Htr3a+ basket cells rely on glutamatergic neurotransmission. In contrast, the survival of bipolar cells depends on long-range serotonergic inputs.

Wong et al., 2022, Cell Reports *40*, 111037
July 5, 2022 © 2022 The Authors.
https://doi.org/10.1016/j.celrep.2022.111037

CellPress

Cell Reports

CellPress
OPEN ACCESS

Article

Serotonergic regulation of bipolar cell survival in the developing cerebral cortex

Fong Kuan Wong,[1,2,4] Martijn Selten,[1,2,5] Claudia Rosés-Novella,[1,2,5] Varun Sreenivasan,[1,2] Noemí Pallas-Bazarra,[1,2] Eleni Serafeimidou-Pouliou,[1,2] Alicia Hanusz-Godoy,[1,2] Fazal Oozeer,[1,2] Robert Edwards,[3] and Oscar Marín[1,2,6,*]
[1]Centre for Developmental Neurobiology, Institute of Psychiatry, Psychology and Neuroscience, King's College London, London SE1 1UL, UK
[2]MRC Centre for Neurodevelopmental Disorders, King's College London, London SE1 1UL, UK
[3]Department of Physiology and Department of Neurology, School of Medicine, University of California San Francisco, San Francisco, CA, USA
[4]Present address: Division of Developmental Biology and Medicine, Faculty of Biology, Medicine and Health, University of Manchester, Manchester M13 9PT, UK
[5]These authors contributed equally
[6]Lead contact
*Correspondence: oscar.marin@kcl.ac.uk
https://doi.org/10.1016/j.celrep.2022.111037

SUMMARY

One key factor underlying the functional balance of cortical networks is the ratio of excitatory and inhibitory neurons. The mechanisms controlling the ultimate number of interneurons are beginning to be elucidated, but to what extent similar principles govern the survival of the large diversity of cortical inhibitory cells remains to be investigated. Here, we investigate the mechanisms regulating developmental cell death in neurogliaform cells, bipolar cells, and basket cells, the three main populations of interneurons originating from the caudal ganglionic eminence and the preoptic region. We found that all three subclasses of interneurons undergo activity-dependent programmed cell death. However, while neurogliaform cells and basket cells require glutamatergic transmission to survive, the final number of bipolar cells is instead modulated by serotonergic signaling. Together, our results demonstrate that input-specific modulation of neuronal activity controls the survival of cortical interneurons during the critical period of programmed cell death.

INTRODUCTION

A large diversity of interneurons, defined by specific morphological, electrophysiological, and molecular properties, enables the great variety of inhibitory motifs that exist in the cerebral cortex (Lim et al., 2018). The contribution of each interneuron subtype to the modulation of information processing in cortical circuits largely depends on their number and precise connectivity patterns (Fishell and Rudy, 2011; Jiang et al., 2015; Kätzel et al., 2011), which are both refined during distinct developmental critical periods. Dysregulation of these processes has been associated neurodevelopmental disorders such as autism and epilepsy (Marín, 2016).

The final number of neurons in the developing brain is regulated through an evolutionary-conserved process known as programmed cell death (Hamburger and Levi-Montalcini, 1949; Oppenheim, 1991; Raff et al., 1993). In the cerebral cortex, glutamatergic pyramidal cells and gamma-aminobutyric acid-expressing (GABAergic) interneurons are first overproduced during neurogenesis (Southwell et al., 2012; Thomaidou et al., 1997). Subsequently, many neurons are eliminated to establish the final ratios of excitatory and inhibitory neurons (Blanquie et al., 2017; Wong and Marín, 2019). Multiple lines of evidence suggest that cortical neurons undergo programmed cell death unless they sustain a certain level of activity during a specific period of development (Blanquie et al., 2017; Denaxa et al., 2018; Duan et al., 2020; Priya et al., 2018; Wong et al., 2018). This process ensures that only active neurons are fully integrated into nascent circuits (Duan et al., 2020; Wong et al., 2018), and appropriate ratios of excitatory and inhibitory neurons are retained in the cerebral cortex.

One population of interneurons has been shown to flout this requirement—the vasoactive intestinal peptide-expressing (VIP+) interneurons (Duan et al., 2020; Priya et al., 2018). VIP+ interneurons are overproduced during development and subsequently pruned through programmed cell death. However, in contrast to all other cortical interneurons, neuronal activity does not appear to impact their maturation or survival (De Marco García et al., 2011; Priya et al., 2018), which suggests that the density of VIP+ interneurons in the cerebral cortex might be established independently of their ability to integrate into the nascent circuits. This possibility is intriguing since many VIP+ interneurons directly regulate the function of other interneurons (Fu et al., 2014; García-Junco-Clemente et al., 2017; Lee et al., 2013; Pfeffer et al., 2013; Pi et al., 2013), and disruption of their development causes functional deficits that are associated with autism spectrum disorder (ASD) (Batista-Brito et al., 2017; Goff and Goldberg, 2019; Mossner et al., 2020). Elucidating the mechanisms that determine the final complement of VIP+ interneurons is critical for understanding the organization of disinhibitory circuits in the cerebral cortex.

This is an open access article under the CC BY license (http://creativecommons.org/licenses/by/4.0/).

One limitation of previous attempts at investigating the development of VIP+ interneurons is the heterogeneity of this population. VIP+ interneurons belong to a large and diverse group of cortical interneurons that, in the mouse, expresses the serotonin receptor Htr3a (Rudy et al., 2011). It consists of at least three major subclasses: reelin-expressing (Reln+) neurogliaform cells, calretinin-expressing (CR+) bipolar cells, and basket cells, many of which also express the neuropeptide cholecystokinin (Lim et al., 2018). Most Htr3a+ interneurons are produced during development from the caudal ganglionic eminence (CGE) (Butt et al., 2005; Miyoshi et al., 2010; Nery et al., 2005), but at least a fraction of neurogliaform cells seems to derive from progenitor cells in the preoptic-hypothalamic (POH) border region (Gelman et al., 2009; Lim et al., 2018; Niquille et al., 2018). VIP is expressed by most CR+ bipolar cells and many CGE-derived basket cells (Prönneke et al., 2015), so, in principle, it is conceivable that these two subtypes of VIP+ interneurons may follow different developmental programs.

In contrast to previous work, here we found that the survival of all three main subclasses of cortical Htr3a+ interneurons is regulated by neuronal activity during a critical time window in postnatal mouse development. This process is dependent on the ability of interneurons to integrate into nascent cortical circuits, as inputs from pyramidal cells can regulate their survival. Remarkably, while neurogliaform cells and CGE-derived basket cells depend on glutamatergic transmission for their survival, bipolar cells do not. Instead, we found that serotonin is a potent modulator of the survival of bipolar cells. Our experiments reveal a mechanism through which long-range inputs from distant brain regions regulate the final number of a specific subtype of interneuron in the developing cerebral cortex.

RESULTS

Activity-dependent survival of Htr3a+ interneurons

It has been previously shown that Reln+ cells and VIP+ cells undergo Bax-mediated programmed cell death in the postnatal cortex, although only Reln+, but not VIP+, cells appear to depend on activity for their survival (Priya et al., 2018). Since VIP is expressed in both bipolar cells and some CGE-derived basket cells (hereafter referred to as basket cells) (Prönneke et al., 2015), we designed a strategy to identify the three main subclasses of Htr3a+ interneurons simultaneously. We used triple immunohistochemistry against Prox1, a transcription factor that is universally expressed in Htr3a+ interneurons (Rubin and Kessaris, 2013), CR, and Reln, to discriminate neurogliaform cells (Prox1+ and Reln+), bipolar cells (Prox1+ and CR+), and basket cells (Prox1+, CR−, and Reln−) (Figure S1; see STAR Methods). We validated this approach by comparing the percentage of cells recovered in the cerebral cortex with previous studies based on genetic methods (Lim et al., 2018) (Figure S1). We then used Htr3a-Cre mice to generate conditional mice in which all Htr3a+ interneurons lack the two BCL2 family genes Bax and Bak1 (Lindsten et al., 2000) and quantified the number of neurogliaform cells, bipolar cells, and basket cells in the primary somatosensory cortex (S1) at postnatal day 30 (P30). We found that suppressing programmed cell death leads to a ~30% increase in the density of the three main subclasses of Htr3a+ interneurons (Figure 1). These results confirmed that neurogliaform cells, bipolar cells, and basket cells undergo programmed cell death in the developing postnatal cortex.

We next investigated whether cell-autonomous manipulation of the activity of the three main subclasses of Htr3a+ interneurons influences their survival. To this end, we transiently modified their activity using a chemogenetic approach based on the use of designer receptors exclusively activated by designer drugs (DREADDs). In brief, we injected the S1 of P0 Htr3a-Cre mice with adeno-associated viruses (AAVs) encoding mutant G-protein-coupled receptors that either enhance (hM3Dq) or dampen (hM4di) neuronal activity following the administration of the ligand clozapine-N-oxide (CNO) (Figure 2A). We administered CNO to pups twice daily between P7 and P10, which overlaps with the peak of programmed cell death for Htr3a+ interneurons (Priya et al., 2018), and examined their distribution at P21. In contrast to previous observations (Priya et al., 2018), we found that cell-autonomous alteration of neuronal activity modified the density of all Htr3a+ interneurons, including the subtypes characterized by the expression of VIP, bipolar cells (Prox1+ and CR+), and basket cells (Prox1+, CR-, and Reln-). Specifically, activation of Htr3a+ interneurons led to increased density of neurogliaform cells, bipolar cells, and basket cells (Figures 2B and 2C). Conversely, inhibition of Htr3a+ interneurons decreased the final density of all three subclasses of interneurons (Figures 2B and 2D). The role of activity in regulating the survival of Htr3a+ interneurons is associated with their standard period of programmed cell death because cell-autonomous manipulation of neuronal activity between P10 and P13 did not modify their density (Figure S2). Altogether, these results demonstrated that all Htr3a+ interneurons, including VIP+ subtypes, undergo activity-dependent programmed cell death in the mouse during a defined time window of early postnatal development.

Pyramidal cells regulate the survival of Htr3a+ interneurons

We have previously shown that the survival of interneurons originating from the medial ganglionic eminence (MGE) and the preoptic area (POA) is regulated by the number and activity of excitatory pyramidal cells (Wong et al., 2018). We investigated whether the survival of Htr3a+ interneurons also depends on the number of these cells. To this end, we generated mice in which pyramidal cells lack *Bax* and *Bak1* and fail to undergo programmed cell death (Wong et al., 2018). Analysis of S1 at P30 revealed that the densities of neurogliaform cells, bipolar cells, and basket cells were significantly increased in $Nex^{Cre/+}$;$Bak1^{-/-}$;$Bax^{fl/fl}$ mice compared with controls (Figures 3A and 3B). This experiment revealed that the number of pyramidal cells influences the survival of all three main groups of Htr3a+ interneurons.

We next investigated whether changes in the activity of pyramidal cells would also influence the survival of Htr3a+ interneurons. To this end, we injected the S1 of $Nex^{Cre/+}$ mice with AAVs encoding hM3Dq or hM4Di at P0, modified the activity of pyramidal cells by administrating CNO between P7 and P10, and analyzed the distribution of interneurons at P21 (Figure 4A). We found that the activation of pyramidal cells led to an increase

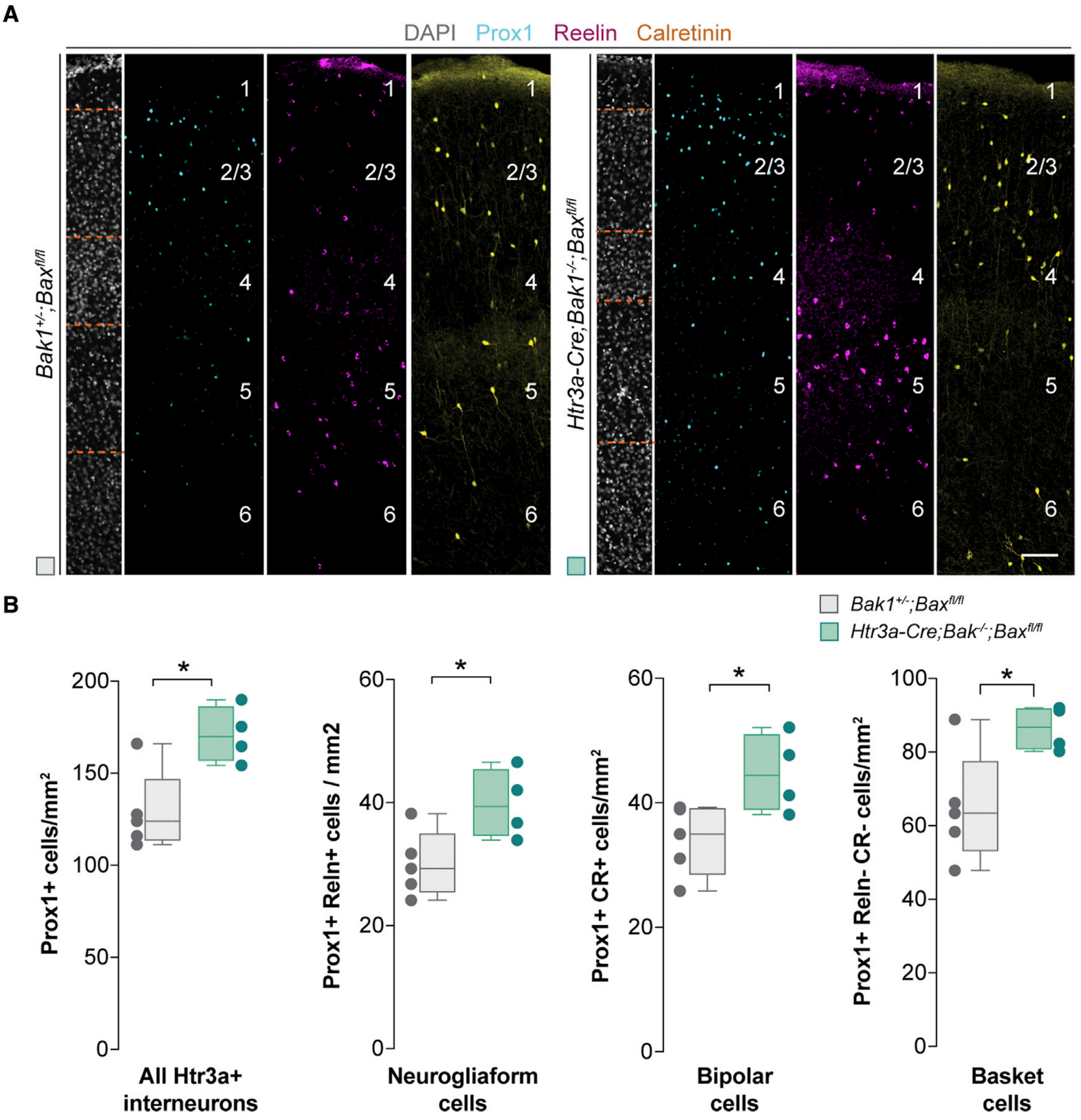

Figure 1. The three main subclasses of Htr3a+ interneurons undergo programmed cell death

(A) Coronal sections through the primary somatosensory cortex of control and *Htr3a-Cre*;*Bak*$^{-/-}$;*Bax*$^{fl/fl}$ mice at P30 following immunohistochemistry against Prox1 (cyan), reelin (magenta), and calretinin (yellow). DAPI is shown for counterstaining (grey).

(B) Quantification of the density of all Htr3a+ interneurons (Prox1+), neurogliaform cells (Prox1+ and Reln+), bipolar cells (Prox1+ and CR+), and basket cells (Prox1+, Reln−, and CR−) in control (gray boxplots, n = 5 mice) and *Htr3a-Cre*;*Bak*$^{-/-}$;*Bax*$^{fl/fl}$ mice (green boxplots, n = 4 mice) at P30. Prox1+: two-tailed unpaired Student's t test, *p = 0.01. Prox1+ Reln+: two-tailed unpaired Student's t test, *p = 0.03. Prox1+ and CR+: two-tailed unpaired Student's t test, *p = 0.03. Prox1+, Reln−, and CR−: two-tailed unpaired Student's t test, *p = 0.03.

Data in (B) are shown as boxplots (median, middle dash), lower and upper quartiles (box borders), and minimum and maximum (whiskers), and the adjacent data points indicate the average cell density in each animal. Scale bar, 100 μm. See also Figure S1.

in the density of neurogliaform cells, bipolar cells, and basket cells (Figures 4B and 4C), whereas dampening the activity of pyramidal cells decreased the density of all three subclasses of interneurons (Figures 4B and 4D). In contrast, modifying the activity of pyramidal cells after the period of interneuron programmed cell death did not modify the density of Htr3a+ interneurons (Figure S3). These experiments revealed that bidirectional modulation of the activity of pyramidal cells during the standard period of interneuron programmed cell death influences the survival of these cells.

Bipolar cell survival is independent of glutamatergic transmission

Our previous experiments demonstrated that pyramidal cells have a non-cell autonomous role in regulating the survival of Htr3a+ interneurons. We hypothesized that the depolarizing

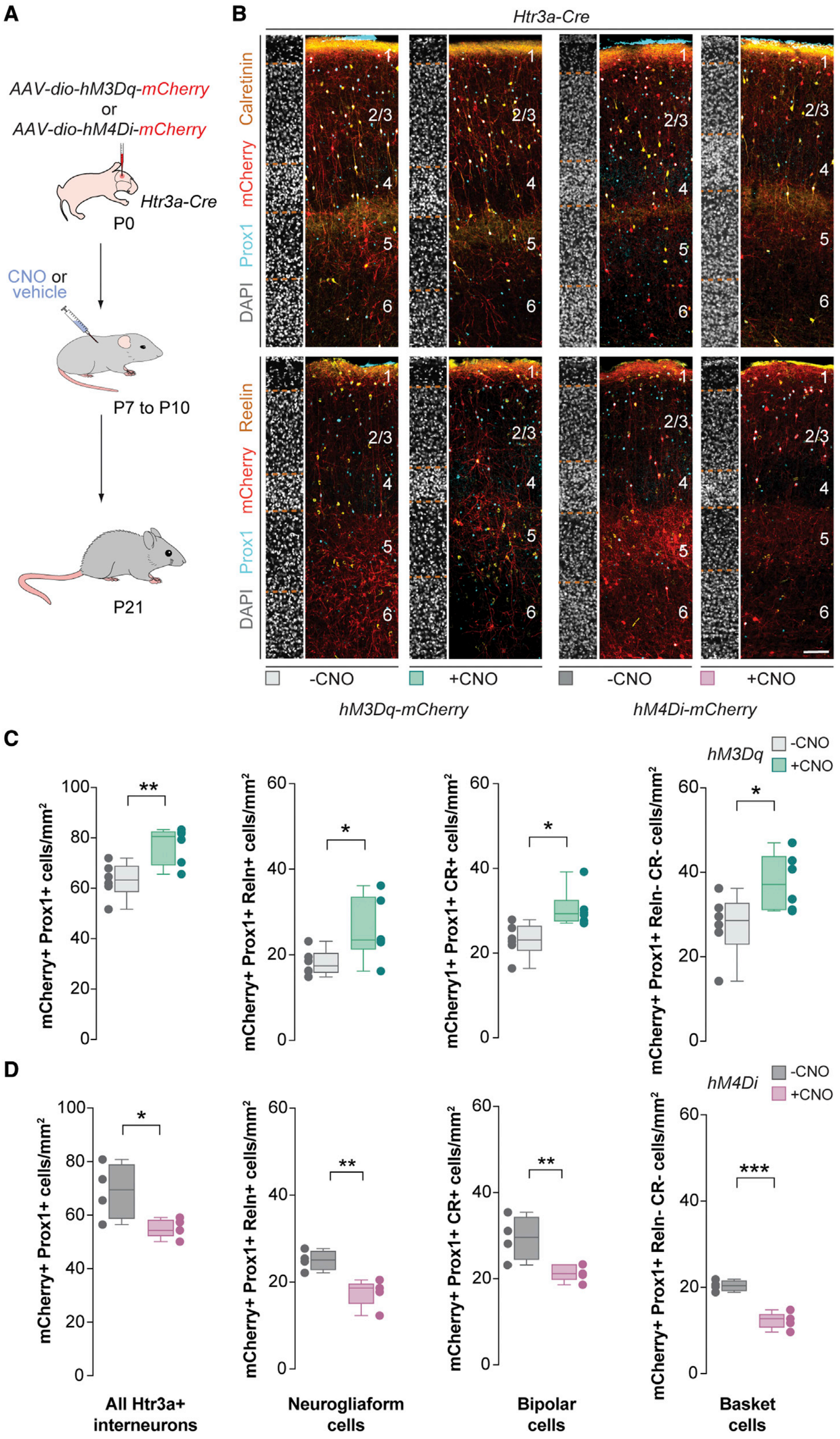

(legend on next page)

effect of glutamatergic transmission could mediate this effect. To test this hypothesis, we prevented exocytotic glutamate release from pyramidal cells by generating mice lacking the vesicular glutamate transporters Vglut1 and Vglut2 in cortical neurons. We deleted both vesicular glutamate transporters simultaneously because the deletion of one of them can be functionally compensated by the other in thalamic neurons (Li et al., 2013). Although Vglut1 is the primary transporter expressed by cortical neurons, Vglut2 is transiently expressed in the cortex during the first week of postnatal development (Fremeau et al., 2004). Because null *Vglut2* mice die at birth (Moechars et al., 2006), we crossed null *Vglut1* mice (Fremeau et al., 2004) with floxed *Vglut2* mice (Hnasko et al., 2010) and injected Cre-expressing AAVs in the neocortex of control and $Vglut1^{-/-}$;$Vglut2^{fl/fl}$ mice at P1 (Figure 5A). Consistent with the observation that the number and activity of pyramidal cells influence the survival of Htr3a+ interneurons, we observed a prominent decrease in the density of neurogliaform cells and basket cells (collectively identified as Prox1+ and CR− cells) in the cortex of Cre-injected $Vglut1^{-/-}$;$Vglut2^{fl/fl}$ mice compared with controls at P21 (Figures 5B and 5C). In contrast, we observed no significant difference in the density of bipolar cells between both experimental groups (Figures 5B and 5C). We also found that the removal of *Vglut1* from pyramidal cells is not sufficient to affect the survival of Htr3a+ interneurons (Figure S4), which indicates that *Vglut2* seems to be able to compensate for the loss of Vglut1 in pyramidal cells during the time window of interneuron cell death. These results revealed that glutamatergic transmission mediates the role of pyramidal cells in the survival of neurogliaform cells and basket cells. Unexpectedly, these experiments also demonstrated that although the survival of bipolar cells is modulated by the number and activity of pyramidal cells, this effect does not seem to be mediated by glutamatergic transmission.

Serotonin regulates bipolar cell survival

The unexpected independence of bipolar cell survival from glutamatergic transmission is consistent with the notion that VIP+ interneurons are more poorly integrated into the local excitatory network than MGE-derived interneurons during the period of programmed cell death (Anastasiades et al., 2016; Che et al., 2018; Duan et al., 2020; Vagnoni et al., 2020). This observation led us to hypothesize that the survival of bipolar cells could be regulated by other factors whose activity or release could be influenced by pyramidal cells, either directly or indirectly. Previous work has shown that the modulation of serotonin levels influences cell death in the cerebral cortex (Stankovski et al., 2007). Since Htr3a+ interneurons express functional serotonin receptors (Lee et al., 2010), we tested whether serotonin could modulate the survival of these cells. We first determined whether Htr3a+ interneurons contain functional serotonin receptors during the period of programmed cell death. To this end, we prepared acute slices of *Htr3a-Cre*;*RCE* mice (in which CGE/POH-derived interneurons express GFP) between P7 and P10 and performed voltage-clamp recordings from layer (L) 2/3 S1 GFP+ cells in the presence of synaptic blockers while applying serotonin (100 μM) directly onto the soma of GFP+ cells through a second pipette. Consistent with previous observations, the morphologies of the recovered neurons resembled bipolar and multipolar VIP+ interneurons located in L2/3 (Figure 6A) (Prönneke et al., 2015).

Interestingly, we recorded two different responses from these populations at these stages. Cells with multipolar morphology (putative basket cells) responded to serotonin application with a modest outward current (Figures 6B and 6C). In contrast, bipolar cells exhibited an inward current that, in current-clamp mode, evoked action potentials in six out of the seven cells (Figures 6B and 6C). Focal application of artificial cerebrospinal fluid alone elicited no responses from these cells (Figures S5A and S5B). To examine the effect of serotonin on neurogliaform cells, we recorded GFP+ cells in S1 L1, where the majority of Htr3a+ interneurons belong to this subclass of interneuron (Poorthuis et al., 2018). We found that most L1 GFP+ cells responded to the application of serotonin with a modest outward current (8/9 cells; Figures S5C–S5E). These experiments revealed that bipolar cells constitute the only population of CGE/POH-derived interneurons selectively depolarized by serotonin during programmed cell death.

We next tested whether serotonin affects the survival of bipolar cells. To this end, we modified serotonin levels specifically during Htr3a+ interneuron cell death by administrating the selective serotonin-reuptake inhibitor fluoxetine. We injected fluoxetine twice daily intraperitoneally between P7 and P10 in $Vip^{Cre/+}$;$RCL^{tdTomato}$ mice and studied the distribution of Htr3a+ interneurons at P21. We found that increasing cortical serotonin levels during the period of interneuron cell death led to a significant increase in the density of bipolar cells, with no significant changes in any of the other populations of Htr3a+ interneurons

Figure 2. Cell-autonomous changes in neuronal activity regulate the survival of Htr3a+ interneurons

(A) Schematic of experimental design.

(B) Coronal sections through the primary somatosensory cortex of *Htr3a-Cre* mice at P21 injected with *hM3Dq-mCherry* (left) or *hM4Di-mCherry* (right) virus followed by vehicle or CNO treatment immunostained for Prox1 (cyan), mCherry (red), calretinin (yellow, top), and reelin (yellow, bottom). DAPI is shown for counterstaining (gray).

(C) Quantification of the density of all transfected Htr3a+ interneurons (mCherry+, Prox1+), neurogliaform cells (mCherry+, Prox1+, and Reln+), bipolar cells (mCherry+, Prox1+, and CR+), and basket cells (mCherry+, Prox1+, Reln−, and CR−) in control (gray boxplots, n = 6 mice) and CNO-treated mice injected with *hM3Dq-mCherry* (green boxplots, n = 6 mice) at P21. Prox1+: two-tailed unpaired Student's t test, **p = 0.007. Prox1+ Reln+: two-tailed unpaired Student's t test, *p = 0.04. Prox1+ and CR+: two-tailed unpaired Student's t test, *p = 0.01. Prox1+, Reln−, and CR−: two-tailed unpaired Student's t test, *p = 0.03.

(D) Quantification of the density of all transfected Htr3a+ interneurons (mCherry+, Prox1+), neurogliaform cells (mCherry+, Prox1+, and Reln+), bipolar cells (mCherry+, Prox1+, and CR+), and basket cells (mCherry+, Prox1+, Reln−, and CR−) in control (gray boxplots, n = 4 mice) and CNO-treated mice injected with *hM4Di-mCherry* (red boxplots, n = 5 mice) at P21. Prox1+: two-tailed unpaired Student's t test, *p = 0.03. Prox1+ Reln+: two-tailed unpaired Student's t test, **p = 0.006. Prox1+ and CR+: two-tailed unpaired Student's t test, **p = 0.005. Prox1+, Reln−, and CR−: two-tailed unpaired Student's t test, ***p = 0.0001.

Data in (C) and (D) are shown as boxplots (median, middle dash), lower and upper quartiles (box borders), and minimum and maximum (whiskers), and the adjacent data points indicate the average cell density in each animal. Scale bar, 100 μm. See also Figure S1.

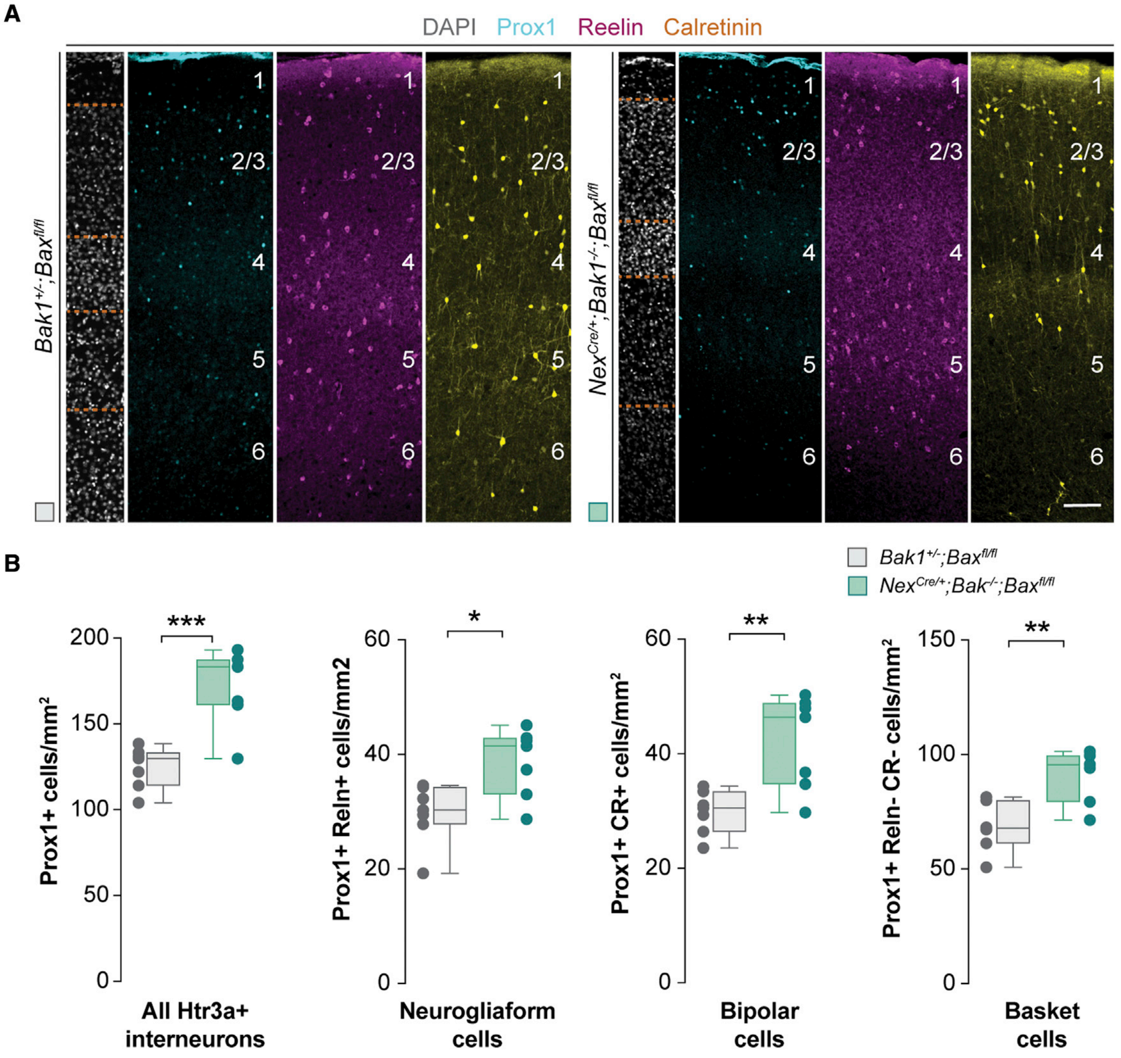

Figure 3. An increased number of pyramidal cells enhances the survival of Htr3a+ interneurons

(A) Coronal sections through the primary somatosensory cortex of control and *Nex*$^{Cre/+}$;*Bak*$^{-/-}$;*Bax*$^{fl/fl}$ mice at P30 following immunohistochemistry against Prox1 (cyan), reelin (magenta), and calretinin (yellow). DAPI is shown for counterstaining (gray).

(B) Quantification of the density of all Htr3a+ interneurons (Prox1+), neurogliaform cells (Prox1+ and Reln+), bipolar cells (Prox1+ and CR+), and basket cells (Prox1+, Reln−, and CR−) in control (gray boxplots, n = 7 mice) and *Nex*$^{Cre/+}$;*Bak*$^{-/-}$;*Bax*$^{fl/fl}$ mice (green boxplots, n = 7 mice) at P30. Prox1+: two-tailed unpaired Student's t test, ***p = 0.0005. Prox1+ Reln+: two-tailed unpaired Student's t test, *p = 0.01. Prox1+ and CR+: two-tailed unpaired Student's t test, **p = 0.004. Prox1+, Reln−, and CR−: two-tailed unpaired Student's t test, **p = 0.002.

Data in (B) are shown as boxplots (median, middle dash), lower and upper quartiles (box borders), and minimum and maximum (whiskers), and the adjacent data points indicate the average cell density in each animal. Scale bar, 100 μm. See also Figures S1 and S2.

(Figures 6D–6F). By contrast, administration of fluoxetine beyond the period of programmed cell death did not impact the final number of bipolar cells present in the cortex (Figures S5F–S5H). Altogether, these experiments suggested that the depolarizing effect of serotonin during the period of programmed cell death regulates the survival of bipolar cells.

Transient expression of vesicular monoamine transporter in pyramidal cells contributes to bipolar cell survival

Our previous experiments demonstrated that both changes in the activity of pyramidal cells and serotonin levels impact the survival of bipolar cells in a manner independent of glutamatergic transmission. Interestingly, although serotonin is exclusively synthesized by raphe neurons in the brainstem, nearly one-third of pyramidal cells in the infragranular layers of the mouse somatosensory cortex transiently express *Slc6A4* and *Slc18a2*, the genes encoding the serotonin and the vesicular monoamine transporters (SERT and VMAT2, respectively) during early postnatal development (Figures S6A and S6B). SERT transports serotonin from the synaptic cleft back into the presynaptic neuron, whereas VMAT2 regulates the packaging of monoamines (including serotonin) into vesicles for subsequent release at the synapse. We hypothesized that the transient

CellPress
OPEN ACCESS

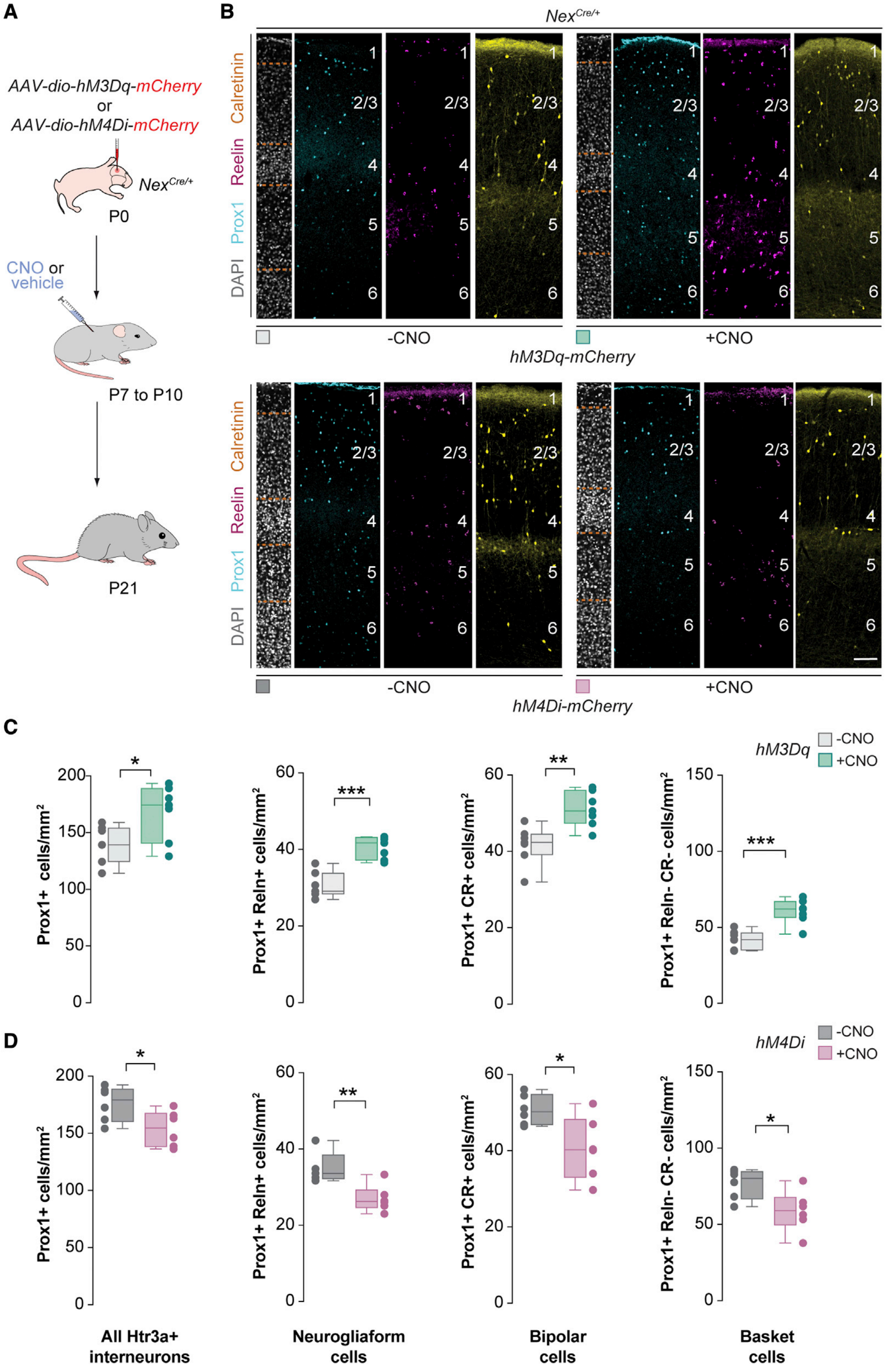

(legend on next page)

expression of these genes might confer pyramidal cells the capability to release serotonin in an activity-dependent manner, thereby contributing to regulating the survival of bipolar cells. To test this hypothesis, we used a conditional gene-knockdown strategy using AAV vectors that express microRNA (miRNA)-based short hairpin RNA against *Slc18a2*, which encodes VMAT2 (Figure S6C). We first confirmed the efficacy of the designed short hairpin RNAs (shRNAs) in downregulating *Slc18a2* expression *in vitro* and *in vivo* (Figures S6D–S6G). We next examined whether preventing monoamine release by pyramidal cells would interfere with the effect that increasing the activity of pyramidal cells during the period of programmed cell death has on the survival of bipolar cells. To this end, we injected AAVs encoding *hM3Dq* and control or *Slc18a2 shRNA* in the cortex of $Nex^{Cre/+}$ mice and then administered pups with CNO twice daily between P7 and P10 (Figures 7A and 7B). We found that knock down of *Slc18a2* in pyramidal cells greatly reduced (by ~50%) the positive effect of pyramidal cell activation on the survival of bipolar cells, with no effect on other types of Htr3a+ interneurons (Figures 7C and 7D). Of note, knocking down *Slc18a2* without increasing the activity of pyramidal cells activity is not sufficient to prevent Htr3a+ interneurons from undergoing programmed cell death (Figures S7A–S7C). These experiments revealed that pyramidal cells contribute to the survival of bipolar cells at least in part by regulating the levels of cortical monoamines such as serotonin during their period of cell death.

DISCUSSION

We have found that the final number of bipolar cells in the mouse cerebral cortex is established through the activity-dependent modulation of programmed cell death by serotonin. In contrast to other cortical interneurons, the survival of bipolar cells does not seem to depend on local glutamatergic transmission but rather on the depolarizing effect of serotonin released in the developing cortex during the period of interneuron programmed cell death. Unexpectedly, we found that pyramidal cells can transiently modulate cortical serotonin levels during this period and therefore influence the survival of bipolar cells. Our findings reveal that long-range serotonergic inputs play a fundamental role in sculping the cellular composition of the cerebral cortex during early postnatal development.

Activity-dependent regulation of interneuron survival

Neuronal activity has been shown to play an essential role in regulating cell maturation and survival (Denaxa et al., 2018; De Marco García et al., 2011; Priya et al., 2018). In contrast to the early specification of neuronal identity by dedicated transcriptional programs (Mayer et al., 2018; Mi et al., 2018), the dependence of neuronal maturation and survival on activity ensures that the neurons that remain in the adult brain are functionally integrated into appropriate neural circuits. We and others have previously shown that in the early postnatal neocortex, MGE/POA-derived interneurons that are inactive have a higher propensity to undergo apoptosis (Wong et al., 2018) and that the modulation of their activity during the period of programmed cell death regulates their survival (Denaxa et al., 2018; Priya et al., 2018). Our current study suggests that the activity-dependent modulation of programmed cell death is universal for cortical interneurons, including the three main subclasses of Hrt3a+ interneurons.

Previous results based on the experimental manipulation of neocortical VIP+ interneurons during development suggested that the survival of these cells does not depend on activity-dependent mechanisms (Priya et al., 2018). Although it is unclear whether these observations related to bipolar cells and/or a subset of VIP+ basket cells, the results nonetheless suggested the existence of interneurons whose survival is independent of neuronal activity. In contrast, we found that the activity of all Htr3a+ interneurons (including neurogliaform cells, bipolar cells, and CGE-derived basket cells) during the period of programmed cell death is directly linked to their survival (Figure 2). The survival of VIP+ interneurons in the hippocampus also depends on activity (Akgul et al., 2019). One possible explanation for these differences is the length of the manipulation. While the chemogenetic approach used in our study allows for a transient manipulation of neuronal activity, the expression of specific ion channels employed by Priya and colleagues led to a permanent change in the activity of these cells. It is conceivable that differences in the duration of the experimental manipulation (i.e., chronic versus acute) may impact activity-dependent biological processes, such as neuronal maturation (De Marco García et al., 2011), or induce compensatory mechanisms to counter the chronic manipulation of neuronal activity.

The activity of a neuron reflects the inputs it receives. Most subclasses of cortical interneurons are well embedded within nascent local circuits during the period of programmed cell

Figure 4. Bidirectional modulation of pyramidal cell activity regulates the survival of Htr3a+ interneuron

(A) Schematic of experimental design.

(B) Coronal sections through the primary somatosensory cortex of $Nex^{Cre/+}$ mice at P21 injected with *hM3Dq-mCherry* virus (top) or *hM4Di-mCherry* (bottom) virus followed by vehicle or CNO treatment immunostained for Prox1 (cyan), reelin (magenta), and calretinin (yellow). DAPI is shown for counterstaining (gray).

(C) Quantification of the density of all Htr3a+ interneurons (Prox1+), neurogliaform cells (Prox1+ and Reln+), bipolar cells (Prox1+ and CR+), and basket cells (Prox1+, Reln−, and CR−) in control (gray boxplots, n = 7 mice) and CNO-treated mice injected with *hM3Dq-mCherry* (green boxplots, n = 7 mice) at P21. Prox1+: two-tailed unpaired Student’s t test, *p = 0.03. Prox1+ and Reln+: two-tailed unpaired Student’s t test, ***p < 0.001. Prox1+ and CR+: two-tailed unpaired Student’s t test, **p = 0.003. Prox1+, Reln−, and CR−: two-tailed unpaired Student’s t test, ***p = 0.0004.

(D) Quantification of the density of all Htr3a+ interneurons (Prox1+), neurogliaform cells (Prox1+ and Reln+), bipolar cells (Prox1+ and CR+), and basket cells (Prox1+, Reln−, and CR−) in control (gray boxplots, n = 6 mice) and CNO-treated mice injected with *hM4Di-mCherry* (red box plots, n = 6 mice) at P21. Prox1+: two-tailed unpaired Student’s t test, *p = 0.04. Prox1+ and Reln+: two-tailed unpaired Student’s t test, **p = 0.007. Prox1+ and CR+: two-tailed unpaired Student’s t test, *p = 0.02. Prox1+, Reln−, and CR−: two-tailed unpaired Student’s t test, *p = 0.02.

Data in (C) and (D) are shown as boxplots (median, middle dash), lower and upper quartiles (box borders), and minimum and maximum (whiskers), and the adjacent data points indicate the average cell density in each animal. Scale bar, 100 μm. See also Figures S1 and S3.

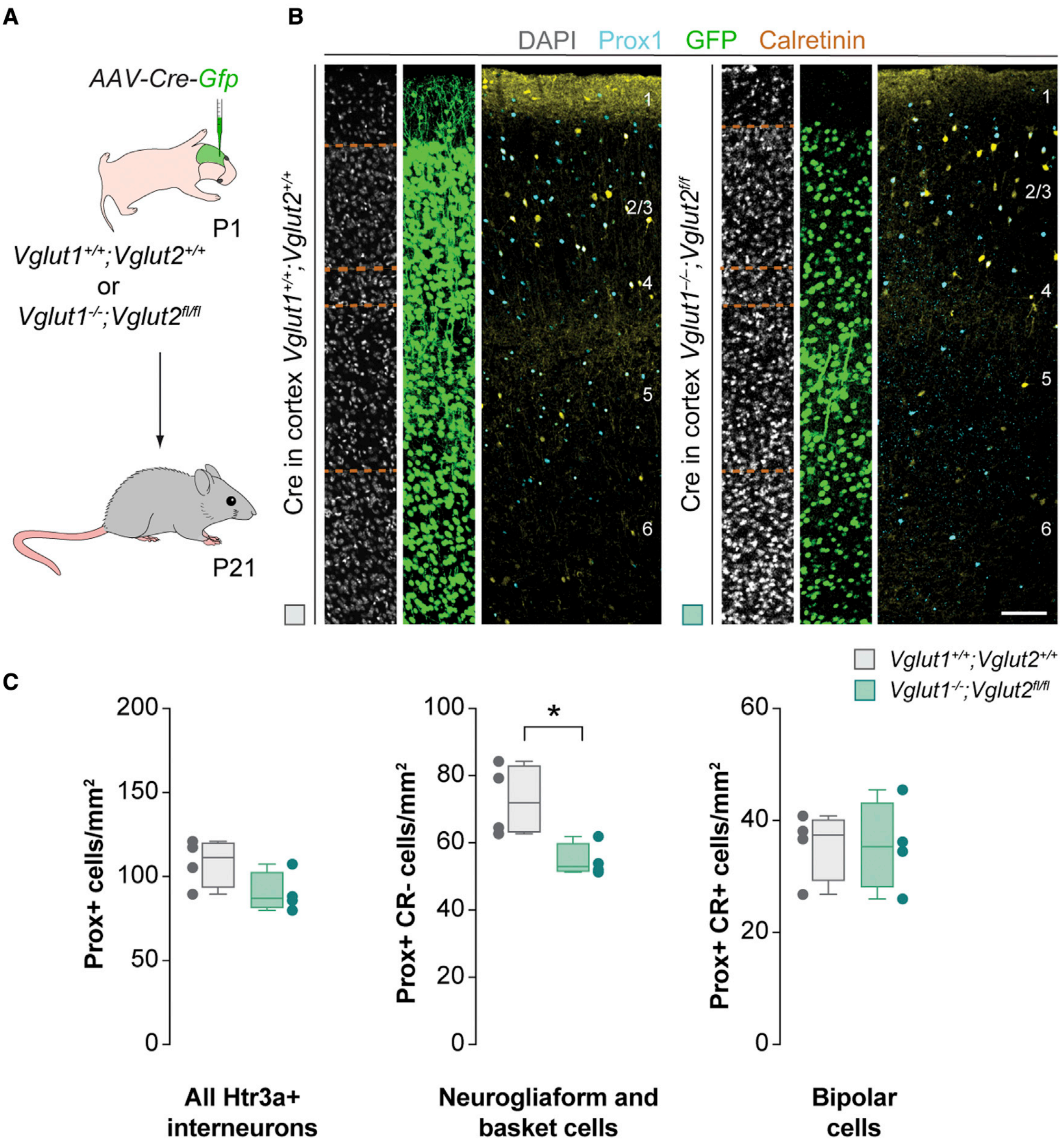

Figure 5. The survival of bipolar cells is independent of glutamate transmission

(A) Schematic of experimental design.

(B) Coronal sections through the primary motor cortex of control and *Vglut1*$^{-/-}$;*Vglut2*$^{fl/fl}$ mice at P21 following immunohistochemistry against Prox1 (cyan), GFP (green), and calretinin (yellow). DAPI is shown for counterstaining (gray).

(C) Quantification of the density of all Htr3a+ interneurons (Prox1+), neurogliaform cells and basket cells (Prox1+ and CR-), and bipolar cells (Prox1+ and CR+) in control (gray boxplots, n = 4 mice) and *Vglut1*$^{-/-}$;*Vglut2*$^{fl/fl}$ mice (green boxplots, n = 4 mice) at P21. Prox1+: two-tailed unpaired Student's t test, p = 0.10. Prox1+ and CR−: two-tailed unpaired Student's t test, *p = 0.02. Prox1+ and CR+: two-tailed unpaired Student's t test, p = 0.99.

Data in (C) are shown as boxplots (median, middle dash), lower and upper quartiles (box borders), and minimum and maximum (whiskers), and the adjacent data points indicate the average cell density in each animal. Scale bar, 100 μm. See also Figures S1 and S4.

death. Consequently, we hypothesized that most cortical interneurons would require glutamatergic transmission from pyramidal cells for survival and found that this was the case for neurogliaform cells and CGE-derived basket cells. Somehow surprisingly, we found that the survival of bipolar cells seems largely independent of cortical glutamatergic transmission. This is consistent with the notion that bipolar cells receive less local glutamatergic inputs (Anastasiades et al., 2016; Che et al., 2018; Duan et al., 2020; Vagnoni et al., 2020) than long-range connections, which include neuromodulatory (e.g., serotonergic and cholinergic) (Lee et al., 2013; Wall et al., 2016; Zhang et al., 2014) as well as other subcortical projections (e.g., thalamocortical) (Kastli et al., 2020). While this long-range regulation of cell survival might be unique for bipolar cells among

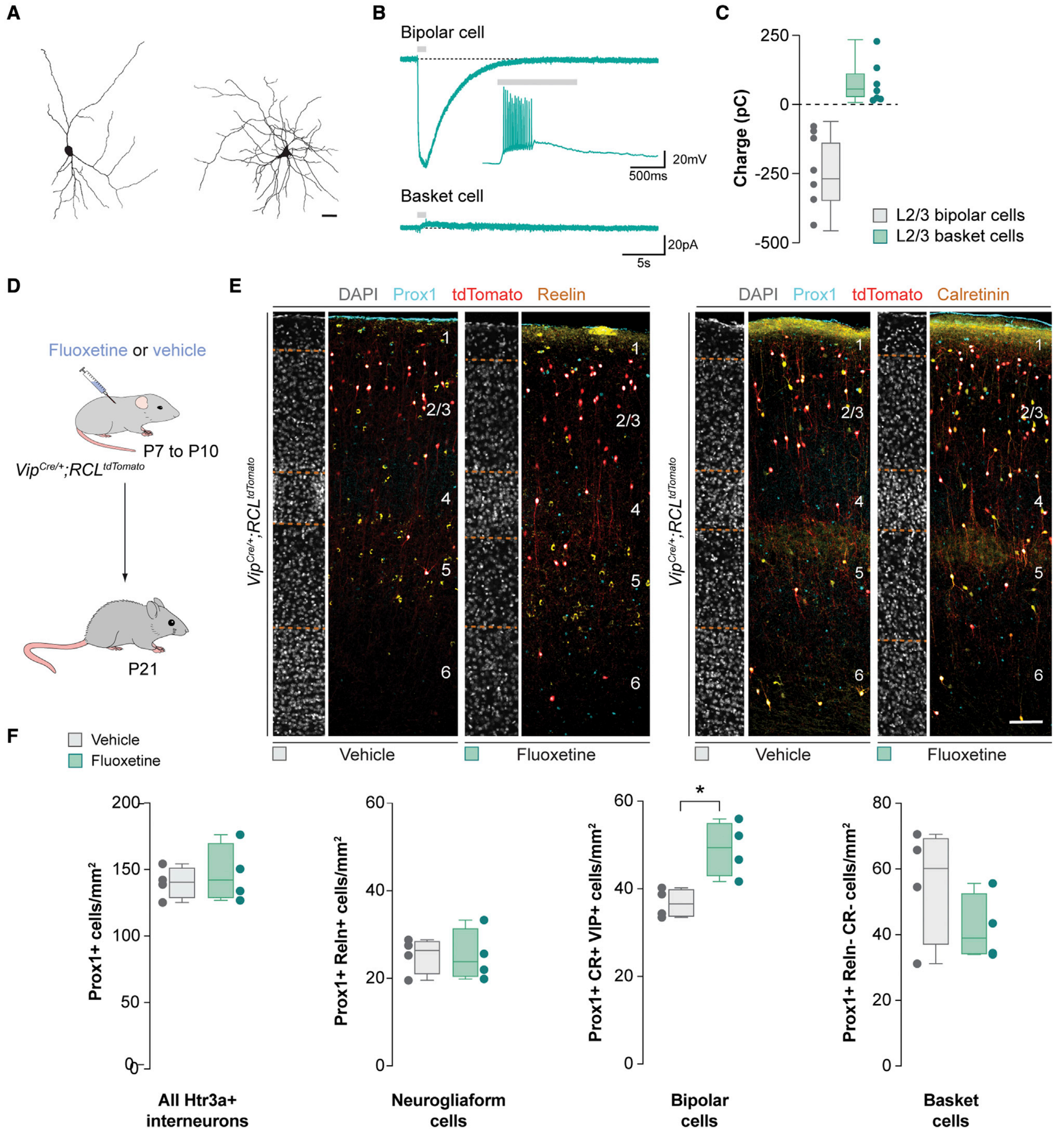

Figure 6. Serotonin selectively depolarizes bipolar cells and regulates their survival

(A) Typical morphology of reconstructed bipolar cells (left) and basket cells (right).

(B) Example of voltage-clamp traces of bipolar cells (top) and basket cells (bottom) in response to serotonin puffs (gray bars). The insert is an example of a current clamp trace of a bipolar cell responding to serotonin puffs (gray bars).

(C) Quantification of cell charge of L2/3 bipolar cells (gray boxplot, n = 7 cells) and basket cells (green boxplot, n = 7 cells).

(D) Schematic of experimental design.

(E) Coronal sections through the primary somatosensory cortex of $Vip^{Cre/+}$;$RCL^{tdTomato}$ mice at P21 injected with vehicle or fluoxetine treatment immunostained for Prox1 (cyan), tdTomato (red), and reelin (yellow, left) or calretinin (yellow, right). DAPI is shown for counterstaining (gray).

(F) Quantification of the density of all Htr3a+ interneurons (Prox1+), neurogliaform cells (Prox1+ and Reln+), bipolar cells (Prox1+, CR+, and VIP+), and basket cells (Prox1+, Reln−, and CR−) in control (gray boxplots, n = 4 mice) and fluoxetine-injected mice (green boxplots, n = 4 mice) at P21. Prox1+: two-tailed unpaired

(legend continued on next page)

cortical interneurons, the survival of at least some types of striatal interneurons also seems to be modulated by the activity of neurons that are not embedded within the local neural network (Sreenivasan et al., 2022).

Selective regulation of bipolar cell survival by serotonin

Gene-expression dynamics play a key role in regulating different aspects of the functional maturation of cortical circuits. Developmental changes in serotonin-receptor expression have been previously described for pyramidal cells (Béïque et al., 2004; Zhang, 2003) but not for interneurons. Previous studies have shown that CGE-derived interneurons across all layers are rapidly depolarized in response to selective Htr3a agonists by the third postnatal week (Lee et al., 2010). However, our results indicate that only bipolar cells, but not neurogliaform cells or basket cells, are strongly depolarized by serotonin during the period of interneuron programmed cell death (Figure 6). Consistently, experimentally increasing brain serotonin levels during this period enhances the survival of bipolar cells but does not affect other classes of CGE/POH-derived interneurons. This suggests a developmental switch in the expression of serotonin receptors in CGE/POH-derived interneurons before the cell-death period, limiting the function of serotonin in regulating cell survival in bipolar cells.

Serotonin plays vital roles in brain development (Teissier et al., 2017). For example, abnormally high serotonin levels during cortical development, as found in mice lacking SERT or monoamine oxidase A (MAOA), disrupt the development of thalamocortical projections (Cases et al., 1996; Persico et al., 2001). Only the serotonergic neurons in the raphe nuclei produce and reuptake serotonin in the adult brain. However, in the developing cortex, transient expression of the serotonin transporter enables thalamocortical axons and pyramidal cells to reuptake serotonin (Narboux-Nême et al., 2008), and disruption of this process disrupts sensory cortex architecture (Chen et al., 2015). Our results indicate that serotonin also regulates the survival of bipolar cells during early postnatal development and that this process is modulated by the activity of pyramidal cells independently of glutamatergic transmission. One mechanism through which pyramidal cells may influence the survival of bipolar cells is by acting as a sink of serotonin, as previously suggested for the regulation of other processes during cortical development (Chen et al., 2015; Soiza-Reilly et al., 2019). However, a prominent fraction of pyramidal cells co-express VMAT2 and SERT, allowing them to recapture serotonin from the extracellular space and load it into synaptic vesicles and release it following their activation (LeBrand et al., 1998; Soiza-Reilly et al., 2019). Since pyramidal cells do not express the transporters for other monoamines such as dopamine and norepinephrine during early mouse postnatal development (LeBrand et al., 1998), these cells may likely release serotonin under certain conditions circumstances. This hypothesis is consistent with the observation that increasing the activity of pyramidal cells enhances the survival of bipolar cells while disrupting glutamatergic transmission has no effect. Altogether, our results suggest that pyramidal cells can transiently modulate the activity of bipolar cells via the regulation of serotonin levels, thereby contributing to the final number of bipolar cells in the developing cortex. This mechanism may enable the integration of long-range and local inputs in establishing the appropriate ratio of this critical type of cortical interneuron.

Functional implications

A comparison of the neuronal composition of the cerebral cortex in rodents and primates has highlighted an evolutionary increase in the relative proportion of certain classes of interneurons. Specifically, the CGE seems to generate a more significant proportion of interneurons in primates than in rodents (Hladnik et al., 2014), which leads to a notable increase in the number of cortical VIP+/CR+ interneurons (Džaja et al., 2014; Krienen et al., 2020). This suggests that bipolar cells may have acquired a more prominent position in cortical circuits and the evolutionary expansion of the superficial layers of the neocortex.

Bipolar cells play critical roles in cortical processing due to their ability to regulate the function of other cortical interneurons (Ayzenshtat et al., 2016; Fu et al., 2014; García-Junco-Clemente et al., 2017; Keller et al., 2020; Lee et al., 2013; Pakan et al., 2016; Pfeffer et al., 2013; Pi et al., 2013). Their dysfunction in mice causes deficits in sensory processing that are reminiscent of alterations found in ASD (Batista-Brito et al., 2017; Goff and Goldberg, 2019; Mossner et al., 2020). Remarkably, elevated serotonin blood levels have been reported in approximately 1 in 4 individuals with ASD, with differences being particularly prominent in children (Gabriele et al., 2014). Rare mutations in SLC6A4 have also been identified in some individuals with ASD (Neale et al., 2012). Furthermore, selective serotonin-reuptake inhibitors (SSRIs) such as fluoxetine are commonly prescribed to treat depression and anxiety disorders, including during pregnancy (Olfson and Marcus, 2009). SSRIs can readily cross the placenta and are present in breast milk (Kim et al., 2006; Rampono et al., 2009), and several studies have suggested that they may contribute to adverse neonatal outcomes (Glover and Clinton, 2016; Grieb and Ragan, 2019; Millard et al., 2017). Thus, although it is presently unclear whether cortical interneurons undergo programmed cell death in humans, our findings suggest that elevated serotonin levels—due to genetic or pharmacological insults—could potentially disrupt cortical architecture in newborns by modifying the density of bipolar cells, a population of interneurons that seems to have greatly expanded during evolution.

Limitations of the study

Our study implicates pyramidal cells in regulating the survival of Htr3a+ interneurons. Our experiments suggest that while the survival of neurogliaform cells and basket cells depends on glutamate neurotransmission, the survival of bipolar cells

Student's t test, p = 0.61. Prox1+ and Reln+: two-tailed unpaired Student's t test, p = 0.98. Prox1+, mCherry+, and CR+: two-tailed unpaired Student's t test: *p = 0.01. Prox1+, CR−, and Reln−: two-tailed unpaired Student's t test, p = 0.23.

Data in (C) and (F) are shown as boxplots (median, middle dash), lower and upper quartiles (box borders), and minimum and maximum (whiskers), and the adjacent data points indicate the charge of each cell (C) and average cell density in each animal (F). Scale bar, 100 μm. See also Figures S1 and S5.

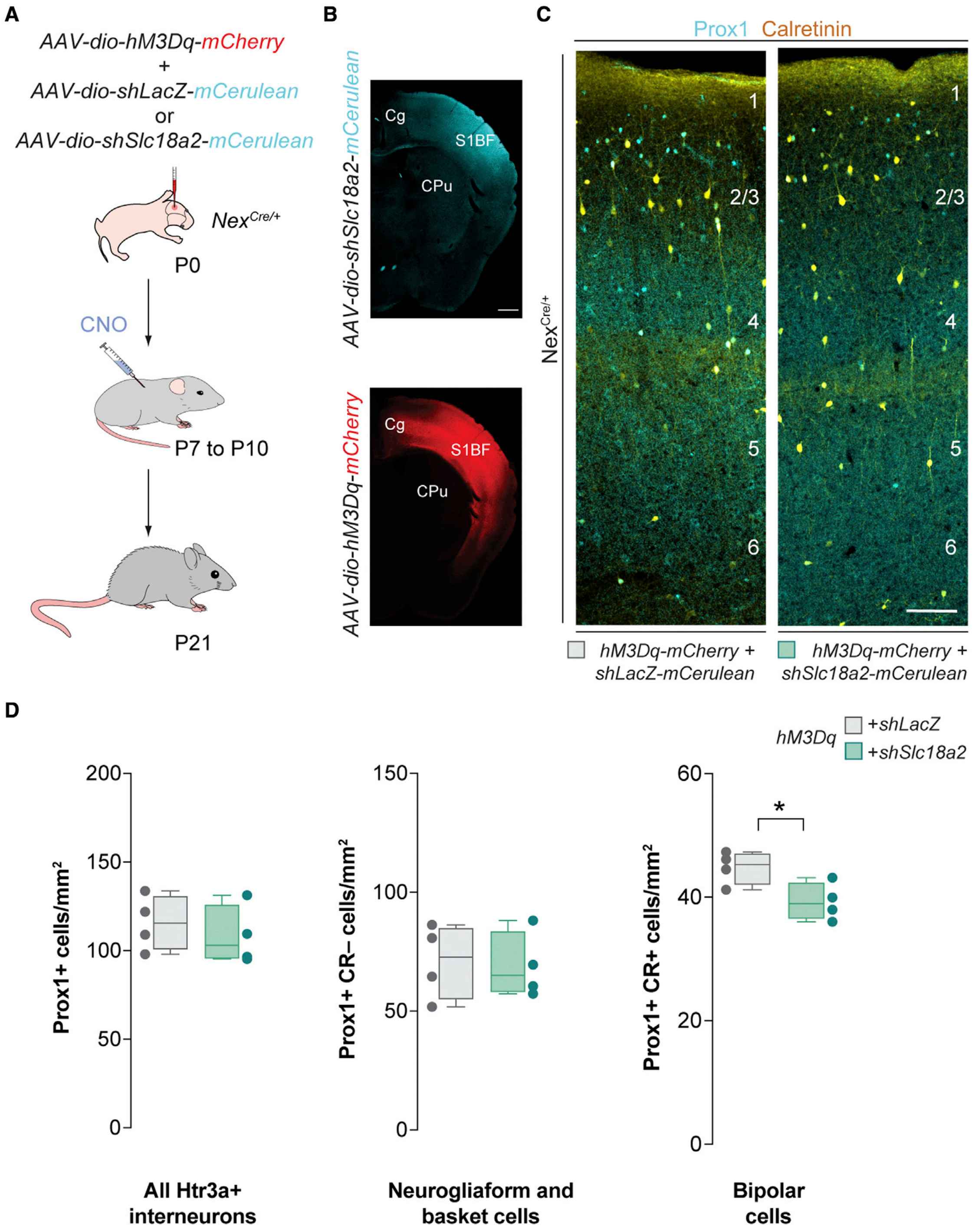

Figure 7. The survival of bipolar cells depends on monoamine transmission from pyramidal cells

(A) Schematic of experimental design.

(B) mCerulean (top) and mCherry (bottom) expression at P21 following AAV injections at P0.

(C) Coronal sections through the primary somatosensory cortex of *Nex*$^{Cre/+}$ mice at P21 injected with *hM3Dq-mCherry* and *shLacZ-mCerulean* (left) or *shSlc18a2-mCerrulean* (right) virus followed by CNO treatment immunostained for Prox1 (cyan) and calretinin (yellow).

(D) Quantification of the density of all Htr3a+ interneurons (Prox1+), neurogliaform cells and basket cells (Prox1+ and CR-), and bipolar cells (Prox1+ and CR+) in *shLacZ-mCerulean* (gray boxplots, n = 4 mice) and *shSlc18a2-mCerrulean* mice (green boxplots, n = 4 mice) at P21. Prox1+: two-tailed unpaired Student's t test, p = 0.53. Prox1+ and CR−: two-tailed unpaired Student's t test, p = 0.85. Prox1+ and CR+: two-tailed unpaired Student's t test, *p = 0.03.

Data in (C) are shown as boxplots (median, middle dash), lower and upper quartiles (box borders), and minimum and maximum (whiskers), and the adjacent data points indicate the average cell density in each animal. Scale bar, 100 μm. See also Figures S1, S6, and S7.

CellPress
OPEN ACCESS

depends on the levels of serotonin during the period of programmed cell death. We have shown that a population of infragranular pyramidal cells express both VMAT2 and SERT during early postnatal development, which would allow these cells to reuptake and load serotonin into synaptic vesicles. However, we have not demonstrated that these pyramidal cells are indeed capable of releasing serotonin. Further electrophysiological studies would be required to demonstrate that pyramidal cells release serotonin when stimulated. Moreover, preventing serotonin release via knock down of VMAT2 only partially reduces the effect of activating pyramidal cells on the survival of bipolar cells. This partial reduction highlights two possible limitations in this study: (1) the relative inefficiency of the shRNA approach to completely deplete *Slc18a2* expression, and (2) the existence of other mechanisms mediating the role of pyramidal cells in the survival of bipolar cell survival besides glutamate and serotonin. A further limitation of our study relates to identifying the specific serotonin receptors involved in regulating bipolar cell survival. Most CGE/POH-derived interneurons express a combination of ionotropic and metabotropic serotonin receptors, and so further experiments would be required to identify the specific serotonin receptors involved in this process.

STAR★METHODS

Detailed methods are provided in the online version of this paper and include the following:

- KEY RESOURCES TABLE
- RESOURCE AVAILABILITY
  - Lead contact
  - Materials availability
  - Data and code availability
- EXPERIMENTAL MODELS AND SUBJECT DETAILS
- METHODS DETAILS
  - Generation of DNA constructs
  - AAV production
  - Intracranial injections
  - Electrophysiology
  - Drugs
  - Histology
  - Single-molecule fluorescent *in situ* hybridization histochemistry
  - Image acquisition and image analysis
- QUANTIFICATION AND STATISTICAL ANALYSIS

SUPPLEMENTAL INFORMATION

Supplemental information can be found online at https://doi.org/10.1016/j.celrep.2022.111037.

ACKNOWLEDGMENTS

We thank I. Andrews for excellent technical assistance, K. Nave (Max Planck Institute for Experimental Medicine, Germany) for *Nex*$^{Cre/+}$ mice, J. Burrone and B. Rico for critical reading of the manuscript, and members of the Marín and Rico laboratories for stimulating discussions and ideas. This work was supported by grants from the European Research Council (ERC-2017-AdG 787355) and the European Union's Horizon 2020 Research and Innovation Programme AIMS-2-TRIALS (777394) to O.M.

AUTHOR CONTRIBUTIONS

F.K.W. and O.M. conceived the study. F.K.W. performed most experiments described in this manuscript. F.K.W. and C.R.-N. performed initial experiments establishing the programmed cell death of Htr3a+ interneurons. M.S. performed electrophysiological experiments. V.S., E.S.-P., and A.H.-G. contributed to data collection. F.O. prepared AAVs. N.P.-B. validated the shRNA constructs and viruses. R.E. contributed *Vglut1* and *Vglut2* mice. F.K.W. and O.M. wrote the paper with input from all authors.

DECLARATION OF INTERESTS

The authors declare no competing interests.

Received: October 9, 2021
Revised: March 9, 2022
Accepted: June 11, 2022
Published: July 5, 2022

REFERENCES

Akgül, G., Abebe, D., Yuan, X.Q., Auville, K., and McBain, C.J. (2019). The role of AMPARs in the maturation and integration of caudal ganglionic eminence-derived interneurons into developing hippocampal microcircuits. Sci. Rep. *9*, 5435. https://doi.org/10.1038/s41598-019-41920-9.

Anastasiades, P.G., Marques-Smith, A., Lyngholm, D., Lickiss, T., Raffiq, S., Kätzel, D., Miesenböck, G., and Butt, S.J.B. (2016). GABAergic interneurons form transient layer-specific circuits in early postnatal neocortex. Nat. Commun. *7*, 10584. https://doi.org/10.1038/ncomms10584.

Ayzenshtat, I., Karnani, M.M., Jackson, J., and Yuste, R. (2016). Cortical control of spatial resolution by VIP+ interneurons. J. Neurosci. *36*, 11498–11509. https://doi.org/10.1523/jneurosci.1920-16.2016.

Batista-Brito, R., Vinck, M., Ferguson, K.A., Chang, J.T., Laubender, D., Lur, G., Mossner, J.M., Hernandez, V.G., Ramakrishnan, C., Deisseroth, K., et al. (2017). Developmental dysfunction of VIP interneurons impairs cortical circuits. Neuron *95*, 884–895.e9. https://doi.org/10.1016/j.neuron.2017.07.034.

Béïque, J.-C., Campbell, B., Perring, P., Hamblin, M.W., Walker, P., Mladenovic, L., and Andrade, R. (2004). Serotonergic regulation of membrane potential in developing rat prefrontal cortex: coordinated expression of 5-hydroxytryptamine 5-HT1A, 5-HT2A, and 5-HT7 receptors. J. Neurosci. *24*, 4807–4817. https://doi.org/10.1523/jneurosci.5113-03.2004.

Blanquie, O., Yang, J.-W., Kilb, W., Sharopov, S., Sinning, A., and Luhmann, H.J. (2017). Electrical activity controls area-specific expression of neuronal apoptosis in the mouse developing cerebral cortex. Elife *6*, e27696. https://doi.org/10.7554/elife.27696.

Butt, S.J.B., Fuccillo, M., Nery, S., Noctor, S., Kriegstein, A., Corbin, J.G., and Fishell, G. (2005). The temporal and spatial origins of cortical interneuron s predict their physiological subtype. Neuron *48*, 591–604. https://doi.org/10.1016/j.neuron.2005.09.034.

Cases, O., Vitalis, T., Seif, I., De Maeyer, E., Sotelo, C., and Gaspar, P. (1996). Lack of barrels in the somatosensory cortex of monoamine oxidase A-deficient mice: role of a serotonin excess during the critical period. Neuron *16*, 297–307. https://doi.org/10.1016/s0896-6273(00)80048-3.

Che, A., Babij, R., Iannone, A.F., Fetcho, R.N., Ferrer, M., Liston, C., Fishell, G., and De Marco García, N.V. (2018). Layer I interneurons sharpen sensory maps during neonatal development. Neuron *99*, 98–116.e7. https://doi.org/10.1016/j.neuron.2018.06.002.

Chen, X., Ye, R., Gargus, J.J., Blakely, R.D., Dobrenis, K., and Sze, J.Y. (2015). Disruption of transient serotonin accumulation by non-serotonin producing neurons impairs cortical map development. Cell Rep. *10*, 346–358. https://doi.org/10.1016/j.celrep.2014.12.033.

Chittajallu, R., Craig, M.T., McFarland, A., Yuan, X., Gerfen, S., Tricoire, L., Erkkila, B., Barron, S.C., Lopez, C.M., Liang, B.J., et al. (2013). Dual origins of functionally distinct O-LM interneurons revealed by differential 5-HT3AR expression. Nat. Neurosci. *16*, 1598–1607. https://doi.org/10.1038/nn.3538.

Denaxa, M., Neves, G., Rabinowitz, A., Kemlo, S., Liodis, P., Burrone, J., and Pachnis, V. (2018). Modulation of apoptosis controls inhibitory interneuron number in the cortex. Cell Rep. *22*, 1710–1721. https://doi.org/10.1016/j.celrep.2018.01.064.

Duan, Z.R.S., Che, A., Chu, P., Modol, L., Bollmann, Y., Babij, R., Fetcho, R.N., Otsuka, T., Fuccillo, M.V., Liston, C., et al. (2020). GABAergic restriction of network dynamics regulates interneuron survival in the developing cortex. Neuron *105*, 75–92.e5. https://doi.org/10.1016/j.neuron.2019.10.008.

Džaja, D., Hladnik, A., Bičanić, I., Baković, M., and Petanjek, Z. (2014). Neocortical calretinin neurons in primates: increase in proportion and microcircuitry structure. Front. Neuroanat. *8*, 103. https://doi.org/10.3389/fnana.2014.00103.

Favuzzi, E., Deogracias, R., Marques-Smith, A., Maeso, P., Jezequel, J., Exposito-Alonso, D., Balia, M., Kroon, T., Hinojosa, A.J., F Maraver, E., and Rico, B. (2019). Distinct molecular programs regulate synapse specificity in cortical inhibitory circuits. Science *363*, 413–417. https://doi.org/10.1126/science.aau8977.

Fishell, G., and Rudy, B. (2011). Mechanisms of inhibition within the telencephalon: "where the wild things are". Annu. Rev. Neurosci. *34*, 535–567. https://doi.org/10.1146/annurev-neuro-061010-113717.

Fremeau, R.T., Kam, K., Qureshi, T., Johnson, J., Copenhagen, D.R., Storm-Mathisen, J., Chaudhry, F.A., Nicoll, R.A., and Edwards, R.H. (2004). Vesicular glutamate transporters 1 and 2 target to functionally distinct synaptic release sites. Science *304*, 1815–1819. https://doi.org/10.1126/science.1097468.

Fu, Y., Tucciarone, J.M., Espinosa, J.S., Sheng, N., Darcy, D.P., Nicoll, R.A., Huang, Z.J., and Stryker, M.P. (2014). A cortical circuit for gain control by behavioral state. Cell *156*, 1139–1152. https://doi.org/10.1016/j.cell.2014.01.050.

Gabriele, S., Sacco, R., and Persico, A.M. (2014). Blood serotonin levels in autism spectrum disorder: a systematic review and meta-analysis. Eur. Neuropsychopharmacol *24*, 919–929. https://doi.org/10.1016/j.euroneuro.2014.02.004.

De Marco García, N.V., Karayannis, T., and Fishell, G. (2011). Neuronal activity is required for the development of specific cortical interneuron subtypes. Nature *472*, 351–355. https://doi.org/10.1038/nature09865.

García-Junco-Clemente, P., Ikrar, T., Tring, E., Xu, X., Ringach, D.L., and Trachtenberg, J.T. (2017). An inhibitory pull-push circuit in frontal cortex. Nat. Neurosci. *20*, 389–392. https://doi.org/10.1038/nn.4483.

Gelman, D.M., Martini, F.J., Nóbrega-Pereira, S., Pierani, A., Kessaris, N., and Marín, O. (2009). The embryonic preoptic area is a novel source of cortical GABAergic interneurons. J. Neurosci. *29*, 9380–9389. https://doi.org/10.1523/jneurosci.0604-09.2009.

Glover, M.E., and Clinton, S.M. (2016). Of rodents and humans: a comparative review of the neurobehavioral effects of early life SSRI exposure in preclinical and clinical research. Int. J. Dev. Neurosci. *51*, 50–72. https://doi.org/10.1016/j.ijdevneu.2016.04.008.

Goebbels, S., Bormuth, I., Bode, U., Hermanson, O., Schwab, M.H., and Nave, K.A. (2006). Genetic targeting of principal neurons in neocortex and hippocampus of NEX-Cre mice. Genesis *44*, 611–621. https://doi.org/10.1002/dvg.20256.

Goff, K.M., and Goldberg, E.M. (2019). Vasoactive intestinal peptide-expressing interneurons are impaired in a mouse model of Dravet syndrome. Elife *8*, e46846. https://doi.org/10.7554/eLife.46846.

Grieb, Z.A., and Ragan, C.M. (2019). The effects of perinatal SSRI exposure on anxious behavior and neurobiology in rodent and human offspring. Eur. Neuropsychopharmacol *29*, 1169–1184. https://doi.org/10.1016/j.euroneuro.2019.07.239.

Hamburger, V., and Levi-Montalcini, R. (1949). Proliferation, differentiation and degeneration in the spinal ganglia of the chick embryo under normal and experimental conditions. J. Exp. Zool. *111*, 457–501. https://doi.org/10.1002/jez.1401110308.

Hladnik, A., DÅ¾ aja, D., Darmopil, S., Jovanov-MiloÅ¸eviÄ‡, N., and Petanjek, Z. (2014). Spatio-temporal extension in site of origin for cortical calretinin neurons in primates. Front. Neuroanat. *8*, 50. https://doi.org/10.3389/fnana.2014.00050.

Hnasko, T.S., Chuhma, N., Zhang, H., Goh, G.Y., Sulzer, D., Palmiter, R.D., Rayport, S., and Edwards, R.H. (2010). Vesicular glutamate transport promotes dopamine storage and glutamate corelease in vivo. Neuron *65*, 643–656.

Jiang, X., Shen, S., Cadwell, C.R., Berens, P., Sinz, F., Ecker, A.S., Patel, S., and Tolias, A.S. (2015). Principles of connectivity among morphologically defined cell types in adult neocortex. Science *350*, aac9462. https://doi.org/10.1126/science.aac9462.

Kastli, R., Vighagen, R., van der Bourg, A., Argunsah, A.Ö., Iqbal, A., Voigt, F.F., Kirschenbaum, D., Aguzzi, A., Helmchen, F., and Karayannis, T. (2020). Developmental divergence of sensory stimulus representation in cortical interneurons. Nat. Commun. *11*, 5729. https://doi.org/10.1038/s41467-020-19427-z.

Kätzel, D., Zemelman, B.V., Buetfering, C., Wölfel, M., and Miesenböck, G. (2011). The columnar and laminar organization of inhibitory connections to neocortical excitatory cells. Nat. Neurosci. *14*, 100–107. https://doi.org/10.1038/nn.2687.

Keller, A.J., Dipoppa, M., Roth, M.M., Caudill, M.S., Ingrosso, A., Miller, K.D., and Scanziani, M. (2020). A disinhibitory circuit for contextual modulation in primary visual cortex. Neuron *108*, 1181–1193.e8. https://doi.org/10.1016/j.neuron.2020.11.013.

Kim, J., Riggs, K.W., Misri, S., Kent, N., Oberlander, T.F., Grunau, R.E., Fitzgerald, C., and Rurak, D.W. (2006). Stereoselective disposition of fluoxetine and norfluoxetine during pregnancy and breast-feeding. Br. J. Clin. Pharmacol. *61*, 155–163. https://doi.org/10.1111/j.1365-2125.2005.02538.x.

Krashes, M.J., Koda, S., Ye, C., Rogan, S.C., Adams, A.C., Cusher, D.S., Maratos-Flier, E., Roth, B.L., and Lowell, B.B. (2011). Rapid, reversible activation of AgRP neurons drives feeding behavior in mice. J. Clin. Invest. *121*, 1424–1428. https://doi.org/10.1172/JCI46229.

Krienen, F.M., Goldman, M., Zhang, Q., C H del Rosario, R., Florio, M., Machold, R., Saunders, A., Levandowski, K., Zaniewski, H., Schuman, B., et al. (2020). Innovations present in the primate interneuron repertoire. Nature *586*, 262–269. https://doi.org/10.1038/s41586-020-2781-z.

LeBrand, C., Cases, O., Wehrlé, R., Blakely, R.D., Edwards, R.H., and Gaspar, P. (1998). Transient developmental expression of monoamine transporters in the rodent forebrain. J. Comp. Neurol. *401*, 506–524. https://doi.org/10.1002/(sici)1096-9861(19981130)401:4<506::aid-cne5>3.0.co;2-#.

Lee, S., Hjerling-Leffler, J., Zagha, E., Fishell, G., and Rudy, B. (2010). The largest group of superficial neocortical GABAergic interneurons expresses ionotropic serotonin receptors. J. Neurosci. *30*, 16796–16808. https://doi.org/10.1523/jneurosci.1869-10.2010.

Lee, S., Kruglikov, I., Huang, Z.J., Fishell, G., and Rudy, B. (2013). A disinhibitory circuit mediates motor integration in the somatosensory cortex. Nat. Neurosci. *16*, 1662–1670. https://doi.org/10.1038/nn.3544.

Li, H., Fertuzinhos, S., Mohns, E., Hnasko, T.S., Verhage, M., Edwards, R., Sestan, N., and Crair, M.C. (2013). Laminar and columnar development of barrel cortex relies on thalamocortical neurotransmission. Neuron *79*, 970–986. https://doi.org/10.1016/j.neuron.2013.06.043.

Lim, L., Mi, D., Llorca, A., and Marín, O. (2018). Development and functional diversification of cortical interneurons. Neuron *100*, 294–313.

Lindsten, T., Ross, A.J., King, A., Zong, W.-X., Rathmell, J.C., Shiels, H.A., Ulrich, E., Waymire, K.G., Mahar, P., Frauwirth, K., et al. (2000). The combined functions of proapoptotic Bcl-2 Family Members Bak and Bax are essential for normal development of multiple tissues. Mol. Cell *6*, 1389–1399. https://doi.org/10.1016/s1097-2765(00)00136-2.

Madisen, L., Garner, A.R., Shimaoka, D., Chuong, A.S., Klapoetke, N.C., Li, L., van der Bourg, A., Niino, Y., Egolf, L., Monetti, C., et al. (2015). Transgenic mice for intersectional targeting of neural sensors and effectors with high specificity and performance. Neuron *85*, 942–958. https://doi.org/10.1016/j.neuron.2015.02.022.

Marín, O. (2016). Developmental timing and critical windows for the treatment of psychiatric disorders. Nat. Med. *22*, 1229–1238. https://doi.org/10.1038/nm.4225.

Mayer, C., Hafemeister, C., Bandler, R.C., Machold, R., Batista Brito, R., Jaglin, X., Allaway, K., Butler, A., Fishell, G., and Satija, R. (2018). Developmental diversification of cortical inhibitory interneurons. Nature *555*, 457–462. https://doi.org/10.1038/nature25999.

Mi, D., Li, Z., Lynette, L., Li, M., Moissidis, M., Yang, Y., Gao, T., Hu, T.X., Pratt, T., Price, D.J., et al. (2018). Early emergence of cortical interneuron diversity in the mouse embryo. Science *360*, 81–85. https://doi.org/10.1126/science.aar6821.

Millard, S.J., Weston-Green, K., and Newell, K.A. (2017). The effects of maternal antidepressant use on offspring behaviour and brain development: implications for risk of neurodevelopmental disorders. Neurosci. Biobehav. Rev. *80*, 743–765. https://doi.org/10.1016/j.neubiorev.2017.06.008.

Miyoshi, G., Hjerling-Leffler, J., Karayannis, T., Sousa, V.H., Butt, S.J.B., Battiste, J., Johnson, J.E., Machold, R.P., and Fishell, G. (2010). Genetic fate mapping reveals that the caudal ganglionic eminence produces a large and diverse population of superficial cortical interneurons. J. Neurosci. *30*, 1582–1594. https://doi.org/10.1523/jneurosci.4515-09.2010.

Moechars, D., Weston, M.C., Leo, S., Callaerts-Vegh, Z., Goris, I., Daneels, G., Buist, A., Cik, M., van der Spek, P., Kass, S., et al. (2006). Vesicular glutamate transporter VGLUT2 expression levels control quantal size and neuropathic pain. J. Neurosci. *26*, 12055–12066. https://doi.org/10.1523/jneurosci.2556-06.2006.

Mort, R.L., Ford, M.J., Sakaue-Sawano, A., Lindstrom, N.O., Casadio, A., Douglas, A.T., Keighren, M.A., Hohenstein, P., Miyawaki, A., and Jackson, I.J. (2014). Fucci2a: a bicistronic cell cycle reporter that allows Cre mediated tissue specific expression in mice. Cell Cycle *13*, 2681–2696. https://doi.org/10.4161/15384101.2015.945381.

Mossner, J.M., Batista-Brito, R., Pant, R., and Cardin, J.A. (2020). Developmental loss of MeCP2 from VIP interneurons impairs cortical function and behaviour. eLife *9*, e55639.

Narboux-Nême, N., Pavone, L.M., Avallone, L., Zhuang, X., and Gaspar, P. (2008). Serotonin transporter transgenic (SertCre) mouse line reveals developmental targets of serotonin specific reuptake inhibitors (SSRIs). Neuropharmacology *55*, 994–1005. https://doi.org/10.1016/j.neuropharm.2008.08.020.

Neale, B.M., Kou, Y., Liu, L., Ma'ayan, A., Samocha, K.E., Sabo, A., Lin, C.F., Stevens, C., Wang, L.S., Makarov, V., et al. (2012). Patterns and rates of exonic de novo mutations in autism spectrum disorders. Nature *485*, 242–245. https://doi.org/10.1038/nature11011.

Nery, S., Fishell, G., and Corbin, J.G. (2005). The caudal ganglionic eminence is a source of distinct cortical and subcortical cell population. Nat. Neurosci. *5*, 1279–1287.

Niquille, M., Limoni, G., Markopoulos, F., Cadilhac, C., Prados, J., Holtmaat, A., and Dayer, A. (2018). Neurogliaform cortical interneurons derive from cells in the preoptic area. Elife *7*, e32017. https://doi.org/10.7554/elife.32017.

Olfson, M., and Marcus, S.C. (2009). National patterns in antidepressant medication treatment. Arch. Gen. Psychiatr. *66*, 848. https://doi.org/10.1001/archgenpsychiatry.2009.81.

Oppenheim, R.W. (1991). Cell death during development of the nervous system. Annu. Rev. Neurosci. *14*, 453–501. https://doi.org/10.1146/annurev.ne.14.030191.002321.

Pakan, J.M., Lowe, S.C., Dylda, E., Keemink, S.W., Currie, S.P., Coutts, C.A., and Rochefort, N.L. (2016). Behavioral-state modulation of inhibition is context-dependent and cell type specific in mouse visual cortex. Elife *5*, e14985. https://doi.org/10.7554/eLife.14985.

Persico, A.M., Mengual, E., Moessner, R., Hall, S.F., Revay, R.S., Sora, I., Arellano, J., DeFelipe, J., Giménez-Amaya, J.M., Conciatori, M., et al. (2001). Barrel pattern formation requires serotonin uptake by thalamocortical afferents, and not vesicular monoamine release. J. Neurosci. *21*, 6862–6873. https://doi.org/10.1523/jneurosci.21-17-06862.2001.

Pfeffer, C.K., Xue, M., He, M., Huang, Z.J., and Scanziani, M. (2013). Inhibition of inhibition in visual cortex: the logic of connections between molecularly distinct interneurons. Nat. Neurosci. *16*, 1068–1076. https://doi.org/10.1038/nn.3446.

Pi, H.-J., Hangya, B., Kvitsiani, D., Sanders, J.I., Huang, Z.J., and Kepecs, A. (2013). Cortical interneurons that specialize in disinhibitory control. Nature *503*, 521–524. https://doi.org/10.1038/nature12676.

Poorthuis, R.B., Muhammad, K., Wang, M., Verhoog, M.B., Junek, S., Wrana, A., Mansvelder, H.D., and Letzkus, J.J. (2018). Rapid neuromodulation of layer 1 interneurons in human neocortex. Cell Rep. *23*, 951–958. https://doi.org/10.1016/j.celrep.2018.03.111.

Priya, R., Paredes, M.F., Karayannis, T., Yusuf, N., Liu, X., Jaglin, X., Graef, I., Alvarez-Buylla, A., and Fishell, G. (2018). Activity regulates cell death within cortical interneurons through a calcineurin-dependent mechanism. Cell Rep. *22*, 1695–1709. https://doi.org/10.1016/j.celrep.2018.01.007.

Prönneke, A., Scheuer, B., Wagener, R.J., Möck, M., Witte, M., and Staiger, J.F. (2015). Characterizing VIP neurons in the barrel cortex of VIPcre/tdtomato mice reveals layer-specific differences. Cerebr. Cortex *25*, 4854–4868. https://doi.org/10.1093/cercor/bhv202.

Raff, M.C., Barres, B.A., Burne, J.F., Coles, H.S., Ishizaki, Y., and Jacobson, M.D. (1993). Programmed cell death and the control of cell survival: lessons from the nervous system. Science *262*, 695–700. https://doi.org/10.1126/science.8235590.

Rampono, J., Simmer, K., Ilett, K.F., Hackett, L.P., Doherty, D.A., Elliot, R., Kok, C.H., Coenen, A., and Forman, T. (2009). Placental transfer of SSRI and SNRI antidepressants and effects on the neonate. Pharmacopsychiatry *42*, 95–100. https://doi.org/10.1055/s-0028-1103296.

Rubin, A.N., and Kessaris, N. (2013). Prox1: a lineage tracer for cortical interneurons originating in the lateral/caudal ganglionic eminence and preoptic area. PLoS One *8*, e77339. https://doi.org/10.1371/journal.pone.0077339.

Rudy, B., Fishell, G., Lee, S., and Hjerling-Leffler, J. (2011). Three groups of interneurons account for nearly 100% of neocortical GABAergic neurons. Dev. Neurobiol. *71*, 45–61. https://doi.org/10.1002/dneu.20853.

Soiza-Reilly, M., Meye, F.J., Olusakin, J., Telley, L., Petit, E., Chen, X., Mameli, M., Jabaudon, D., Sze, J.Y., and Gaspar, P. (2019). SSRIs target prefrontal to raphe circuits during development modulating synaptic connectivity and emotional behavior. Mol. Psychiatr. *24*, 726–745. https://doi.org/10.1038/s41380-018-0260-9.

Southwell, D.G., Paredes, M.F., Galvao, R.P., Jones, D.L., Froemke, R.C., Sebe, J.Y., Alfaro-Cervello, C., Tang, Y., Garcia-Verdugo, J.M., Rubenstein, J.L., et al. (2012). Intrinsically determined cell death of developing cortical interneurons. Nature *491*, 109–113. https://doi.org/10.1038/nature11523.

Sreenivasan, V., Serafeimidou-Pouliou, E., Exposito-Alonso, D., Bercsenyi, K., Bernard, C., Bae, S.E., Oozeer, F., Hanusz-Godoy, A., Edwards, R.H., and Marín, O. (2022). Input-specific control of interneuron numbers in nascent striatal networks. Proc. Natl. Acad. Sci. U S A. *119*, e2118430119. https://doi.org/10.1073/pnas.2118430119.

Stankovski, L., Alvarez, C., Ouimet, T., Vitalis, T., El-Hachimi, K.H., Price, D., Deneris, E., Gaspar, P., and Cases, O. (2007). Developmental cell death is enhanced in the cerebral cortex of mice lacking the brain vesicular monoamine transporter. J. Neurosci. *27*, 1315–1324. https://doi.org/10.1523/jneurosci.4395-06.2007.

Takeuchi, O., Fisher, J., Suh, H., Harada, H., Malynn, B.A., and Korsmeyer, S.J. (2005). Essential role of BAX, BAK in B cell homeostasis and prevention of autoimmune disease. Proc. Natl. Acad. Sci. U S A. *102*, 11272–11277. https://doi.org/10.1073/pnas.0504783102.

Teissier, A., Soiza-Reilly, M., and Gaspar, P. (2017). Refining the role of 5-HT in postnatal development of brain circuits. Front. Cell. Neurosci. *11*, 139. https://doi.org/10.3389/fncel.2017.00139.

Thomaidou, D., Mione, M.C., Cavanagh, J.F.R., and Parnavelas, J.G. (1997). Apoptosis and its relation to the cell cycle in the developing cerebral cortex. J. Neurosci. *17*, 1075–1085. https://doi.org/10.1523/jneurosci.17-03-01075.1997.

Vagnoni, C., Baruchin, L.J., Ghezzi, F., Ratti, S., Molnár, Z., and Butt, S.J.B. (2020). Ontogeny of the VIP+ interneuron sensory-motor circuit prior to active whisking. Preprint at bioRxiv. https://doi.org/10.1101/2020.07.01.182238.

Vong, L., Ye, C., Yang, Z., Choi, B., Chua, S., Jr., and Lowell, B.B. (2011). Leptin action on GABAergic neurons prevents obesity and reduces inhibitory tone to POMC neurons. Neuron *71*, 142–154. https://doi.org/10.1016/j.neuron.2011.05.028.

Wall, N.R., De La Parra, M., Sorokin, J.M., Taniguchi, H., Huang, Z.J., and Callaway, E.M. (2016). Brain-wide maps of synaptic input to cortical interneurons. J. Neurosci. *36*, 4000–4009. https://doi.org/10.1523/jneurosci.3967-15.2016.

Wong, F.K., and Marín, O. (2019). Developmental cell death in the cerebral cortex. Annu. Rev. Cell Dev. Biol. *35*, 523–542. https://doi.org/10.1146/annurev-cellbio-100818-125204.

Wong, F.K., Bercsenyi, K., Sreenivasan, V., Portalés, A., Fernández-Otero, M., and Marín, O. (2018). Pyramidal cell regulation of interneuron survival sculpts cortical networks. Nature *557*, 668–673. https://doi.org/10.1038/s41586-018-0139-6.

Zhang, S., Xu, M., Kamigaki, T., Hoang Do, J.P., Chang, W.-C., Jenvay, S., Miyamichi, K., Luo, L., and Dan, Y. (2014). Long-range and local circuits for top-down modulation of visual cortex processing. Science *345*, 660–665. https://doi.org/10.1126/science.1254126.

Zhang, Z.-W. (2003). Serotonin induces tonic firing in layer V pyramidal neurons of rat prefrontal cortex during postnatal development. J. Neurosci. *23*, 3373–3384. https://doi.org/10.1523/jneurosci.23-08-03373.2003.

Zolotukhin, S., Byrne, B.J., Mason, E., Zolotukhin, I., Potter, M., Chesnut, K., Summerford, C., Samulski, R.J., and Muzyczka, N. (1999). Recombinant adeno-associated virus purification using novel methods improves infectious titer and yield. Gene Ther. *6*, 973–985. https://doi.org/10.1038/sj.gt.3300938.

CellPress
OPEN ACCESS

STAR★METHODS

KEY RESOURCES TABLE

REAGENT or RESOURCE	SOURCE	IDENTIFIER
**Antibodies**		
Rabbit anti-calretinin (diluted 1:1000)	Swant	Cat: CR7699/3H; RRID: AB 10000321
Chicken anti-GFP (diluted 1:3000)	Aves Labs	Cat: GFP-1020; RRID: AB_10000240
Goat anti-Prox1 (diluted 1:300)	R&D systems	Cat: AF2727; RRID: AB_2170716
Mouse anti-Reelin (CR-50, diluted 1:300)	MBL International	Cat: D223-3; RRID: AB_843523
Goat anti mCherry (diluted in 1:125)	Antibodies-online	Cat: ABIN1440057; RRID: AB_2333093
Rat anti-RFP (diluted 1:500)	ChromoTek	Cat: 5f8-100; RRID: AB_2336064
Alexa Fluor 647 Streptavidin (diluted 1:500)	Jackson ImmunoResearch Labs	Cat: 016-600-084; RRID: AB_2341101
Donkey anti-goat IgG (HL) Alexa Fluor 488 (diluted 1:500)	Molecular Probes	Cat: A-11055; RRID: AB_2534102
Anti-chicken IgG (H + L) Alexa Fluor 488 (diluted 1:500)	Jackson ImmunoResearch Labs	Cat: 703-545-155; RRID: AB_2340375
Donkey anti-rat IgG (H + L) Cy3 (diluted 1:500)	Jackson ImmunoResearch Labs	Cat: 712-165-150; RRID: AB_2340666
Donkey anti-mouse IgG (H + L) Alexa Fluor 647 (diluted 1:500)	Molecular Probes	Cat: A-31571; RRID: AB_162542
Donkey anti-rabbit IgG (H + L) AlexaFluor 647 (diluted 1:500)	Molecular Probes	Cat: A-31573; RRID: AB_2536183
Rabbit anti-calretinin (diluted 1:1000)	Swant	Cat: CR7699/3H; RRID: AB_10000321
Chicken anti-GFP (diluted 1:3000)	Aves Labs	Cat: GFP-1020; RRID: AB_10000240
Goat anti-Prox1 (diluted 1:300)	R&D systems	Cat: AF2727; RRID: AB_2170716
**Bacterial and virus strains**		
AAV-hSyn-DIO-hM3Dq-mCherry (AAV8)	Krashes et al., (2011), Addgene	Cat: 44361
AAV-hSyn-DIO-hM4Di-mCherry (AAV8)	Krashes et al., (2011), Addgene	Cat: 44362
AAV-hSyn-GFP-Cre (AAV8)	UNC Vector Core	N/A
AAV-EF1a-DIO-shSlc18A2-mCerulean (AAV8)	This paper	N/A
AAV-EF1a-DIO-shLacZ-mCerulean (AAV8)	This paper	N/A
pCAG-DiO-mCerulean-WPRE	This paper	N/A
pAAV-EF1a-DiO-mCherry	A gift from Karl Deisseroth, Addgene	Cat: 20299
**Chemicals, peptides, and recombinant proteins**		
Clozapine-N-Oxide	Tocris	Cat:4936-10MG
Fluoxetine hydrochloride	Sigma-Aldrich	Cat: F132-10MG
Serotonin hydrochloride	Sigma-Aldrich	Cat: H9523-25MG
CNQX	Tocris	Cat: 0190
DL-2-Amino-5-Phosphonovaleric acid (AP-V)	Sigma-Aldrich	Cat: A5282-50MG
Picrotoxin (PTX)	Tocris	Cat: 1128
Bovine serum albumin (BSA)	Sigma-Aldrich	Cat: A8806
Normal donkey serum	Sigma-Aldrich	Cat: S30-100ML
4',6-Diamidine-2'-phenylindole dihydrochloride (DAPI)	Sigma-Aldrich	Cat: D9542
Fast Green	Roche	Cat: 06402712001
Isoflurane 100% inhalation vapor liquid	Piramal Critical Care Limited	N/A
Paraformaldehyde	Sigma-Aldrich	Cat: 441244

(*Continued on next page*)

Continued		
REAGENT or RESOURCE	SOURCE	IDENTIFIER
Pentobarbital sodium (Euthatal)	Merial Animal Health Ltd	N/A
Triton X-100	Sigma-Aldrich	Cat: T8787-100ML
0.9% NaCl	Sigma-Aldrich	Cat: S76530
Dulbecco's Modified Eagle Medium	Gibco	Cat: 21969-035
Penicillin/Streptomycin	Gibco	Cat: 15140-122
Critical commercial assays		
ACDBio Multiplex Fluorescent Kit v2	ACDBio	Cat: 323110
Experimental Models: Cell lines		
HEK293FT	ThermoScientific	Cat: R70007
Experimental Models: Organisms/strains		
Mice: $Nex^{Cre/+}$	Gift from K.A. Nave	MGI: 2668659
Mice: $Bak^{-/-}$;$Bax^{fl/fl}$	The Jackson Laboratory	JAX: 006329; RRID: IMSR_JAX:006329
Mice: *Htr3a-Cre*	Gensat	Cat: 037089-UCD<br>RRID: MMRRC 037089-UCD
Mice: $Vip^{Cre/+}$	The Jackson Laboratory	Cat: 010908; RRID: IMSR JAX:010908
Mice: $RCL^{tdT/+}$	The Jackson Laboratory	JAX: 007909; RRID: IMSR_JAX:007909
Mice: *CD1*	Charles River	Crl: CD1(ICR)
Mice: $Slc17a7^{-/-}$ ($Vglut1^{-/-}$)	Fremeau et al., 2004	MGI: 3617803
Mice: $Slc17a6^{fl/fl}$ ($Vglut2^{fl/fl}$)	Hnasko et al. (2010)	MGI: 4879093
Mice: $Fucci2^{fl/fl}$	Mort et al. (2014)	MGI: 5645798
Oligonucleotides		
FW primer for *shSlc18a2*	CTAGGCAAGCTGATCCTGTTCAT<br>CGCCTGACCCACGATGAACAGG<br>ATCAGCTTGCTTTTTG	
RV primer for *shSlc18a2*	AATTCAAAAAGCAAGCTGATCCT<br>GTTCATCGTGGGTCAGGCGATG<br>AACAGGATCAGCTTGC	
RNAScope® Probe-Mm-Slc18a2	ACDBio	Cat: 425331
RNAScope® Probe-MmSlc6A4	ACDBio	Cat: 315851
Software and algorithms		
FIJI (ImageJ)	National Institute of Health	https://fiji.sc/; https://imagej.nih.gov/ij/index.html; RRID: SCR_003070
MATLAB	MathWorks	https://www.mathworks.com/products/matlab.html; RRID: SCR_001622
LAS AF	Leica Microsystems	https://www.leica-microsystems.com/; RRID: SCR_013673
Stereo Investigator	MBF Biosciences	https://www.mbfbioscience.com/stereo-investigator; RRID: SCR_018948
Other		
P-2000 micropipette puller	Sutter Instrument	https://www.leicabiosystems.com/histology-equipment/microtomes/
ZEISS Apotome 2	ZEISS	https://www.zeiss.com/microscopy
Leica TCS-SP8 confocal	Leica	https://www.leica-microsystems.com/
LightCycler® 96	Roche Life Science	N/A
Optima-L100 XP	Beckman-Coulter	N/A

RESOURCE AVAILABILITY

Lead contact

Further information and requests for resources and reagents may be directed to and will be fulfilled by the lead contact, Oscar Marín (oscar.marin@kcl.ac.uk).

Materials availability

All unique reagents generated in this study are available from the lead contact with a completed Materials Transfer Agreement.

Data and code availability

- All data reported in this paper will be shared by the lead contact upon request.
- This paper does not report any original code.
- Any additional information required to reanalyze the data reported in this paper is available from the lead contact upon request.

EXPERIMENTAL MODELS AND SUBJECT DETAILS

The mouse lines $Nex^{Cre/+}$ ($Neurod6^{tm1(cre)Kan}$) (Goebbels et al., 2006), *Htr3a-Cre* (Tg(Htr3a-Cre)NO152Gsat/Mmucd) (Chittajallu et al., 2013), $Vip^{Cre/+}$ (Jackson Laboratory #010908), RCL^{tdT} ($Gt(ROSA)26Sor^{tm9(CAG-tdTomato)Hze}$) (Madisen et al., 2015), $Bak^{-/-}$,$Bax^{fl/fl}$ ($Bak1^{tm1Thsn}$ and Bax^{tm2Sjk}) (Takeuchi et al., 2005), $Vgat^{Cre/+}$ ($Slc32a1^{tm2(Cre)Lowl}$) (Vong et al., 2011), $Vglut1^{-/-}$;$Vglut2^{fl/fl}$ ($Slc17a7^{tm1Edw}$ and $Slc17a6^{tm1.1Thna}$) (Fremeau et al., 2004; Hnasko et al., 2010) and $Fucci2^{fl/fl}$ (Mort et al., 2014) were used in this study. Animals were housed in groups of up to five littermates and maintained under standard, temperature-controlled laboratory conditions. Mice were kept on a 12:12 light/dark cycle and received water and food ad libitum. Approximately the same number of male and female mice were used in our experiments and analyses. Mice were analyzed at P21 or P30, as indicated in the corresponding figure legends, except for the electrophysiological recordings, which were performed between P7 and P10. All animal procedures were approved by the ethical committee (King's College London) and conducted following European regulations and Home Office personal and project licenses under the UK Animals (Scientific Procedures) 1986 Act. We have previously shown that the loss of one Bak allele does not impact programmed cell death (Wong et al., 2018). Based on this and keeping with the 3Rs principles, we chose to use $Bak^{+/-}$;$Bax^{fl/fl}$ mice instead of $Bak^{+/+}$;$Bax^{fl/fl}$ mice as controls to reduce the total number of mice used in our experiments.

METHODS DETAILS

Generation of DNA constructs

To generate the *AAV-EF1a-DIO-shSlc18A2-mCerulean*, the plasmid *pAAV-EF1a-DiO-mCherry* (Addgene 20299) was used as a starting point. Addgene 20299 was first digested with AscI and PacI to remove the mCherry and generate a 5921 bp backbone. The mCerulean was generated by digesting *pCAG-DiO-mCerulean-WPRE* with AscI and PacI. The fragments were purified using a QIAquick gel extraction kit (Qiagen, Cat: 28704) and ligated with the backbone to generate the final construct.

To test the efficacy of the shRNA constructs *in vitro*, we cultured HEK293T cells in Dulbecco's Modified Eagle's medium supplemented with 10% fetal bovine serum, 2 mM glutamine, penicillin (50 units/mL) and streptomycin (50 g/mL). Cell cultures were incubated at 37°C in a humidified atmosphere containing 5% CO2. 24 h after plating, cells were transfected using FuGENE® HD transfection reagent (Promega E2311) at a 1:3 DNA-FuGENE ratio, following the manufacturer's instructions. Seventy-two hours after transfection, cells were processed for mRNA quantification. Total RNA was extracted using TRIzol reagent (Thermo Scientific ref. 15596018) and Direct-Zol RNA Miniprep kit (Zymo Research R2052). To ensure the complete elimination of DNA contaminants, DNase was included in the column treatment. Retrotranscription to cDNA was performed using a RevertAid H Minus First Strand cDNA Synthesis kit (Thermo Scientific ref. K1632). qPCR was carried out in triplicates on a LightCycler 480 Instrument (Roche) using 0.005 ng of initial RNA and SYBR Green PCR Master Mix (Roche). *18S* was used as a reference for *Slc18a2* and *Cerulean* expression level analyses. Primers were designed using OligoPerfect Designer (Thermo Scientific) and validated using Ensembl BLAST (ensemble.org). Sequences were as follows: *Slc18a2* (FW: ACAGCCTCCACTTCCGAAAG, RV: GTGGTAGCCT TGGGTGACTC), *Cerulean* (FW: AGGACGACGGCAACTACAAG, RV: TTGCCGTCCTCCTTGAAGTC), *18S* (FW: GTAACCCGTT GAACCCCATTCGT, RV: GTGTGTACAAAGGGCAGGGACTTAA). Primers were purchased from Sigma-Aldrich and used at 0.5 μM.

AAV production

As described before, AAVs were produced using Polyethylenimine (PEI) transfection of HEK293FT cells (Favuzzi et al., 2019). In brief, DNA and PEI were mixed in the ratio of 1:4 in uncomplemented DMEM and left at room temp for 25 min to form the DNA-PEI complex. The transfection solution was added to each plate and incubated for 72 h at 37°C in 5% CO2. The transfected cells were then scraped off the plates and pelleted. The cell pellet was lysed in buffer containing 50 mN Tris-Cl, 150 mM NaCl and 2 mM MgCl2 and 0.5% sodium deoxycholate and incubated with 100 U/mL Benzonase (Sigma-Aldrich, Cat# E1014 25KU) for 1 h to dissociate particles from membranes. Any remaining contaminants and empty or incomplete viral particles were removed with a discontinuous iodixanol (OptiPrepTM) (Sigma-Aldrich, Cat# D1556) gradient ultracentrifugation using four layers of different iodixanol concentrations of 15, 25, 40 and 58% (Zolotukhin et al., 1999). The viral suspension was loaded on the iodixanol gradient in Quick-seal polyallomer tubes (Beckman-Coulter, Cat# 342414) and spun in a VTi-50 rotor at 50,000 rpm for 75 min at 12°C in an Optima L-100 XP Beckman Coulter ultracentrifuge. The recovered virus fraction was purified by first passing through a 100-kDa molecular weight cut off (MWCO) centrifugal filter (Sartorius Vivaspin, Cat# VS2041) and then through an Amicon Ultra 2 mL Centrifugal filter (Merck Millipore, Cat# UFC210024). Storage buffer (350 mM NaCl and 5% Sorbitol in PBS) was added to the purified virus and stored in 5 μL aliquots at

−80°C. The AAV titer was determined by quantitative polymerase chain reaction (qPCR) using primers for the WPRE sequence present in the construct. The following primers were used: WPRE Forward primer: 5′GGCACTGACAATTCCGTGGT-3′. WPRE Reverse primer: 5′-CGCTGGATTGAGGGCCGAAG-3′. The extracted viral DNA and a serial dilution of the transfer plasmid DNA containing the WPRE sequence were transferred to a 96-wells plate and measured using a LightCycler® 96 instrument (Roche Life Science). AAVs produced had a titer of 2.10 x 10^{13} viral genomes/mL.

Intracranial injections

pAAV8-hSyn-DiO-hM3D(Gq)-mCherry and *pAAV-hSyn-DiO-hM4D(Gi)-mCherry* were gifts from Bryan Roth (Addgene plasmids #44361 and #44362) (Krashes et al., 2011). P0 and P1 mice were anesthetized with isoflurane and mounted in a stereotaxic frame. Pups were injected with 600 nL of *pAAV8-hSyn-DiO-hM3D(Gq)-mCherry* and *pAAV-hSyn-DiO-hM4D(Gi)-mCherry* diluted in PBS or with *pAAV8-EF1a-DiO-shSlc18A2-mCerulean* or *pAAV8-EF1a-DIO-shLacZ-mCerulean* and colored with 0.5% Fast Green (Sigma-Aldrich). Injections were targeted for the somatosensory cortex with an injection rate of 10 nL/s. Injections of *hSyn-GFP-Cre* were targeted to the motor cortex with 600 nL of viruses at a speed of 3 nL/s. We estimate that over 40% of neurons are typically infected in these experiments using this method.

Electrophysiology

Mice were anesthetized with an overdose of sodium pentobarbital before decapitation. Coronal slices (300 μm) were cut using a VT1200S vibratome (Leica) in ice-cold artificial cerebrospinal fluid (ACSF) containing (in mM): 87 NaCl, 11 glucose, 75 sucrose, 2.5 KCl, 1.25 NaH2PO4, 0.5 CaCl2, 7 MgCl2, 26 NaHCO3, oxygenated with 95% O2/5% CO2 and incubated for 1 h at 32°C and subsequently at RT. Slices were transferred to the recording setup 15 min before recording and incubated at 32°C while being continuously oxygenated with 95% O2/5% CO2 in recording ACSF containing: 124 NaCl, 1.25 NaH2PO4, 3 KCl, 26 NaHCO3, 10 Glucose, 2 CaCl2, 1 MgCl2. Pipettes (3–5 MΩ) were made from borosilicate glass capillaries using a PC-10 pipette puller (Narishige) and filled with intracellular solution containing (in mM): 130 potassium-gluconate, 5 KCl, 10 HEPES, 2.5 MgCl2, 4 Na2ATP, 0.4 Na3GTP, 10 sodium-phosphocreatine, 0.6 EGTA (pH 7.2–7.3, 285–295 mOsm) supplemented with 0.2% neurobiotin for patch-clamp recordings or Serotonin Hydrochloride (100 μM) dissolved in recording ACSF for focal application. Focal application was achieved by positioning the 'puffer' pipette approximately 10 μm from the recorded soma and applying 0.8 mbar air pressure for 1 s using a PDES-02DX pneumatic drug ejection system (NPI). Traces were recorded using a Multiclamp 700B amplifier (Molecular Devices), sampled at 20 kHz and filtered at 3 kHz. Cells were recorded in the presence of 6-cyano-7-nitroquinoxaline-2,3-dione (CNQX, 5 μM) and (2R)-amino-5-phosphonovaleric acid (APV, 100 μM) and Picrotoxin (PTX, 100 μM) at a holding potential of −60 mV. Traces were analyzed using Clampfit 10.2. Serotonin Hydrochloride was purchased from Sigma Aldrich. Neurobiotin was purchased from Vector Laboratories. All other drugs were purchased from Tocris Bioscience.

Drugs

For DREADDs experiments, Clozapine-N-Oxide (CNO, Tocris) was dissolved in 5% dimethyl sulfoxide (Sigma-Aldrich) and then diluted with 0.9% saline to 1 mg/mL. Pups were injected with vehicle (0.05% DMSO) or CNO (1 g/10 mL) subcutaneously for 4 days, twice daily. Fluoxetine hydrochloride (Sigma-Aldrich) was dissolved in 0.9% saline to 1 mg/kg. Pups were injected with either vehicle or Fluoxetine intraperitoneally for 4 days, twice daily.

Histology

Mice were anaesthetized with an overdose of sodium pentobarbital and transcardially perfused with saline, followed by 4% paraformaldehyde (PFA). Brains were post-fixed for 2 h at 4°C. Brains were sectioned on a sliding microtome or a vibratome at 60 μm. All primary and secondary antibodies were diluted in PBS containing 0.25% Triton X-100 and 2% BSA. The following antibodies were used rabbit anti-Calretinin (1:1000, Swant), chicken anti-GFP (1:3000, Aves Lab), goat anti-Prox1 (1:300, R&D systems), mouse anti-reelin (CR-50, 1:300, MBL international) and rat anti-RFP (1:500, ChromoTek). We used Alexa Fluor-conjugated secondary antibodies (Jackson ImmunoResearch Labs and Molecular Probes).

Single-molecule fluorescent *in situ* hybridization histochemistry

Single-molecule fluorescent *in situ* hybridization (smFISH) was conducted in combination with immunohistochemistry. Mice were perfused, and brains were fixed overnight in 4% PFA. Brains were immersed in 15% sucrose PBS and followed by 30% sucrose PBS for cryoprotection. Brains were sectioned using a sliding microtome as previously described at 30 μm, and sections were processed according to the ACDBio Multiplexed Fluorescent v2 kit protocol (ACDBio, Cat 323100). Briefly, tissue was pre-treated with a series of H_20_2, antigen retrieval and protease IV steps before incubation with probes for 2h at 40°C. *Slc18a2* and *Slc6a4* probes were purchased from ACDBio. Following this, three amplification steps were conducted to detect the probes. Sections were then stained for mCherry or mCerullean. Corresponding secondary antibodies from molecular probes were then used. When required, samples were counterstained with DAPI. All samples were mounted using Mowiol.

Image acquisition and image analysis

Images used for analysis were obtained on the SP8 confocal microscope (Leica) using the LAS AF software. Samples from the same experimental litter were imaged and analyzed in parallel, using similar laser powers, photo-multiplied gain, and detection filter settings. Cortical layers were identified based on their distinct histological characteristic. L1 was identified as a sparsely populated cell layer. The higher density of L4 distinguished the border between L2/3 and L4. L5 was identified as the layer deep to L4 and above L6, containing less densely packed nuclei. Cell density within cortical layers was quantified manually in a rectangular area, 551.5 μm wide at the pia surface within the somatosensory cortex. Cells were counted without using pseudocolor. A minimum of four sections were quantified for each mouse. The RNAscope signal was quantified using the Fiji plugin *Particle Analyzer* to validate the efficacy of *shSlc18a2*. In all experiments, the quantification of CGE interneurons is limited to Prox1+ cells (Figure S1). Cells that do not express Prox1 are not considered CGE/POH-derived interneurons in our quantification, even though a small fraction of them probably are. The expression of Prox1 and Reln defined neurogliaform cells. Bipolar cells were defined by the expression of Prox1 and CR or Prox1, VIP, and CR, depending on the experiment. CGE basket cells were defined by the expression of Prox1 and the absence of CR and Reln, regardless of the expression of VIP. Approximately half of all CGE basket cells express VIP, but none of them expresses Reln or CR.

QUANTIFICATION AND STATISTICAL ANALYSIS

Results were plotted and tested for statistical significance using Prism 9 (Table S1). The samples were tested for normality using the Shapiro-Wilk normality test. Paired comparisons were analyzed using a two-tailed unpaired Student's *t*-test (normally distributed) and Mann-Whitney test (for not normally distributed). Multiple comparisons with a single variable were analyzed using one-way ANOVA with post hoc Dunnett (comparing the mean of each column with the mean of a control column) or Tukey's multiple comparisons. Statistical significance was considered at p values $\leq$ 0.05. The number of animals for each experiment, type of statistical test used, and exact p values are described in each figure legend and table S1.

Cell Reports, Volume *40*

Supplemental information

Serotonergic regulation of bipolar cell survival in the developing cerebral cortex

Fong Kuan Wong, Martijn Selten, Claudia Rosés-Novella, Varun Sreenivasan, Noemí Pallas-Bazarra, Eleni Serafeimidou-Pouliou, Alicia Hanusz-Godoy, Fazal Oozeer, Robert Edwards, and Oscar Marín

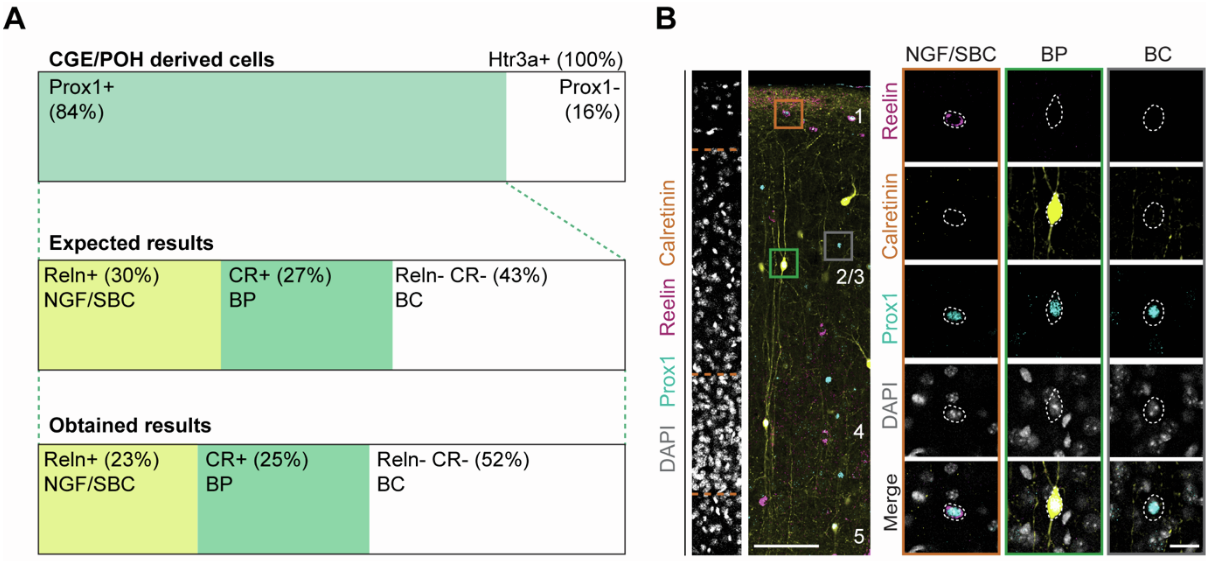

Figure S1. Validation of immunofluorescent strategy related to Figure 1-7

(A) Validation of immunofluorescent strategy. Quantification of percentage of all Htr3a+ interneurons (Prox1+), neurogliaform cells (Prox1+ and Reln+), bipolar cells (Prox1+ and CR+) and CGE-derived basket cells (Prox1+, Reln- and CR-) in wild type mice ($n = 5$ mice) at P21. Chi-squared test, $p = 0.16$.

(B) Coronal sections through the primary somatosensory cortex of wild type mice at P21 (left) with higher magnifications on the right for neurogliaform/single bouquet cells (yellow box), bipolar cells (green box) and basket cells (grey box) immunostained for Prox1 (cyan), mCherry (red) and calretinin (yellow). DAPI is shown for counterstaining (grey).

Scale bars, 100 μm (left), 20 μm (right).

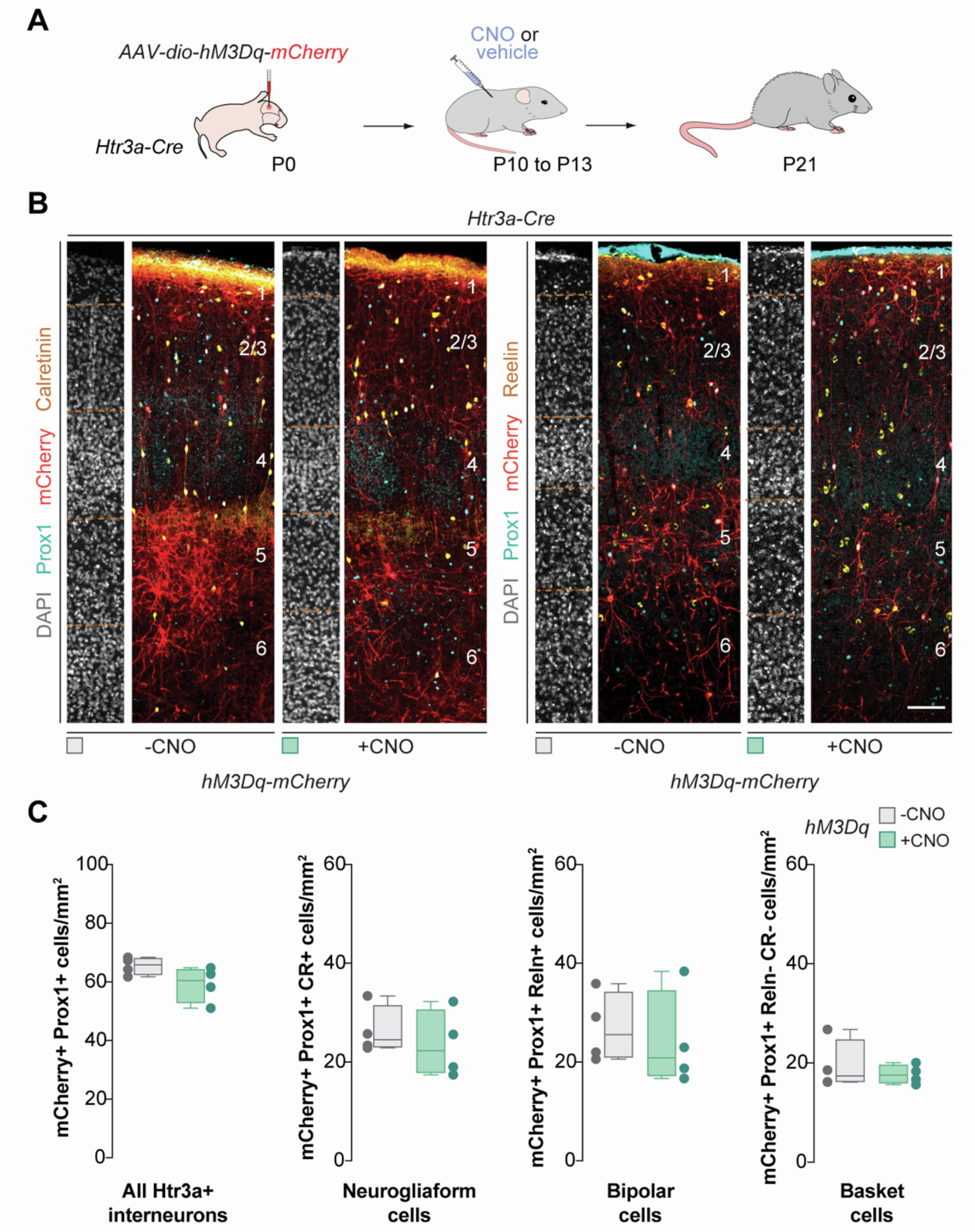

Figure S2. Cell-autonomous changes in neuronal activity beyond the normal period of cell death do not affect the survival of Htr3a+ interneurons related to Figure 3

(A) Schematic of experimental design.

(B) Coronal sections through the primary somatosensory cortex of *Htr3a-Cre* mice at P21 injected with *hM3Dq-mCherry* (left) followed by vehicle or CNO treatment immunostained for Prox1 (cyan), mCherry (red), calretinin (yellow, left) and reelin (yellow, right). DAPI is shown for counterstaining (grey).

(C) Quantification of the density of all transfected Htr3a+ interneurons (mCherry+, Prox1+), neurogliaform cells (mCherry+, Prox1+ and Reln+), bipolar cells (mCherry+, Prox1+ and CR+), and basket cells (mCherry+, Prox1+, Reln- and CR-) in control (grey box plots, $n = 4$ mice) and CNO treated mice injected with *hM3Dq-mCherry* (green box plots, $n = 4$ mice) at P21. Prox1+; two-tailed unpaired Student's t-test, $p = 0.12$. Prox1+ Reln+; two-tailed unpaired Student's t-test, $p = 0.67$. Prox1+ and CR+; two-tailed unpaired Student's t-test, $p = 0.55$. Prox1+, Reln- and CR-; two-tailed unpaired Student's t-test, $p = 0.54$.

Data in panels C are shown as boxplots (median, middle dash), lower and upper quartiles (box borders), and minimum and maximum (whiskers) and the adjacent data points indicate the average cell density in each animal.

Scale bar, 100 μm.

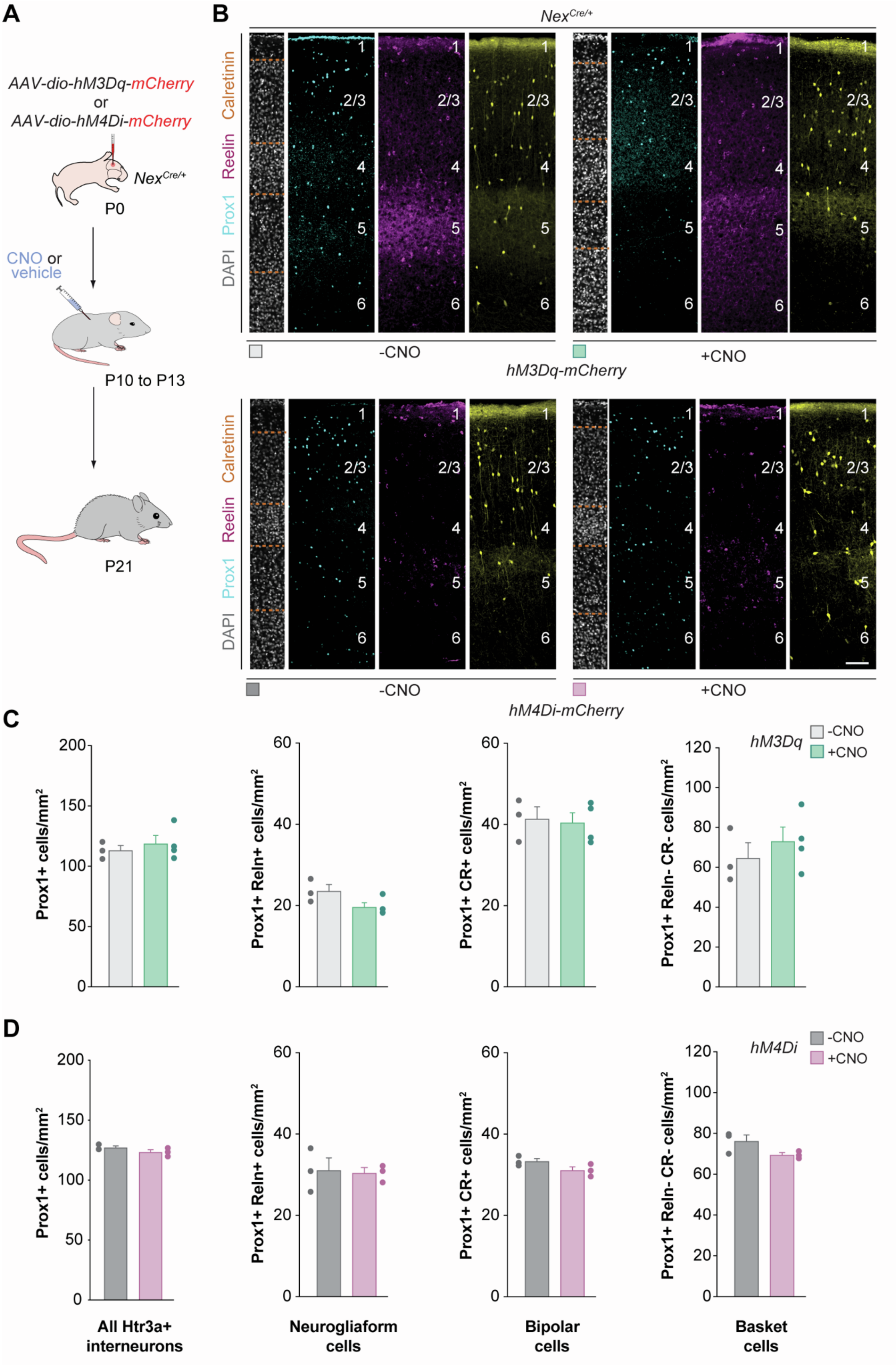

A
AAV-dio-hM3Dq-mCherry
or
AAV-dio-hM4Di-mCherry
Nex^Cre/+
P0
CNO or vehicle
P10 to P13
P21
B
Nex^Cre/+
DAPI Prox1 Reelin Calretinin
1
2/3
4
5
6
-CNO
+CNO
hM3Dq-mCherry
hM4Di-mCherry
C
hM3Dq
-CNO
+CNO
Prox1+ cells/mm²
Prox1+ Reln+ cells/mm²
Prox1+ CR+ cells/mm²
Prox1+ Reln- CR- cells/mm²
D
hM4Di
-CNO
+CNO
Prox1+ cells/mm²
Prox1+ Reln+ cells/mm²
Prox1+ CR+ cells/mm²
Prox1+ Reln- CR- cells/mm²
All Htr3a+ interneurons
Neurogliaform cells
Bipolar cells
Basket cells

Figure S3. Non-cell autonomous changes in neuronal activity beyond the normal period of cell death do not affect the survival of Htr3a+ interneuron related to Figure 4

(A) Schematic of experimental design.

(B) Coronal sections through the primary somatosensory cortex of *Nex*$^{Cre/+}$ mice at P21 injected with *hM3Dq-mCherry* virus (top) or *hM4Di-mCherry* (bottom) virus followed by vehicle (left) or CNO (right) treatment immunostained for Prox1 (cyan), reelin (magenta) and calretinin (yellow). DAPI is shown for counterstaining (grey).

(C) Quantification of the density of all Htr3a+ interneurons (Prox1+), neurogliaform cells (Prox1+ and Reln+), bipolar cells (Prox1+ and CR+), and basket cells (Prox1+, Reln- and CR-) in control (grey box plots, n = 3 mice) and CNO treated mice injected with hM3Dq-mCherry (green box plots, n = 4 mice) at P21. Prox1+; two-tailed unpaired Student's t-test, $p = 0.55$. Prox1+ Reln+; two-tailed unpaired Student's t-test, $p = 0.09$. Prox1+ and CR+; two-tailed unpaired Student's t-test, $p = 0.82$. Prox1+, Reln- and CR-; two-tailed unpaired Student's t-test, $p = 0.47$.

(D) Quantification of the density of all Htr3a+ interneurons (Prox1+), neurogliaform cells (Prox1+ and Reln+), bipolar cells (Prox1+ and CR+), and basket cells (Prox1+, Reln- and CR-) in control (grey box plots, n = 3 mice) and CNO treated mice injected with *hM4Di-mCherry* (red box plots, n = 3 mice) at P21. Prox1+; two-tailed unpaired Student's t-test, $p = 0.20$. Prox1+ Reln+; two-tailed unpaired Student's t-test, $p = 0.85$. Prox1+ and CR+; two-tailed unpaired Student's t-test, $p = 0.12$. Prox1+, Reln- and CR-; two-tailed unpaired Student's t-test, $p = 0.10$.

Data in panels C and D are shown as bar graphs + SEM, and the adjacent data points indicate the average cell density in each animal.

Scale bar, 100 μm.

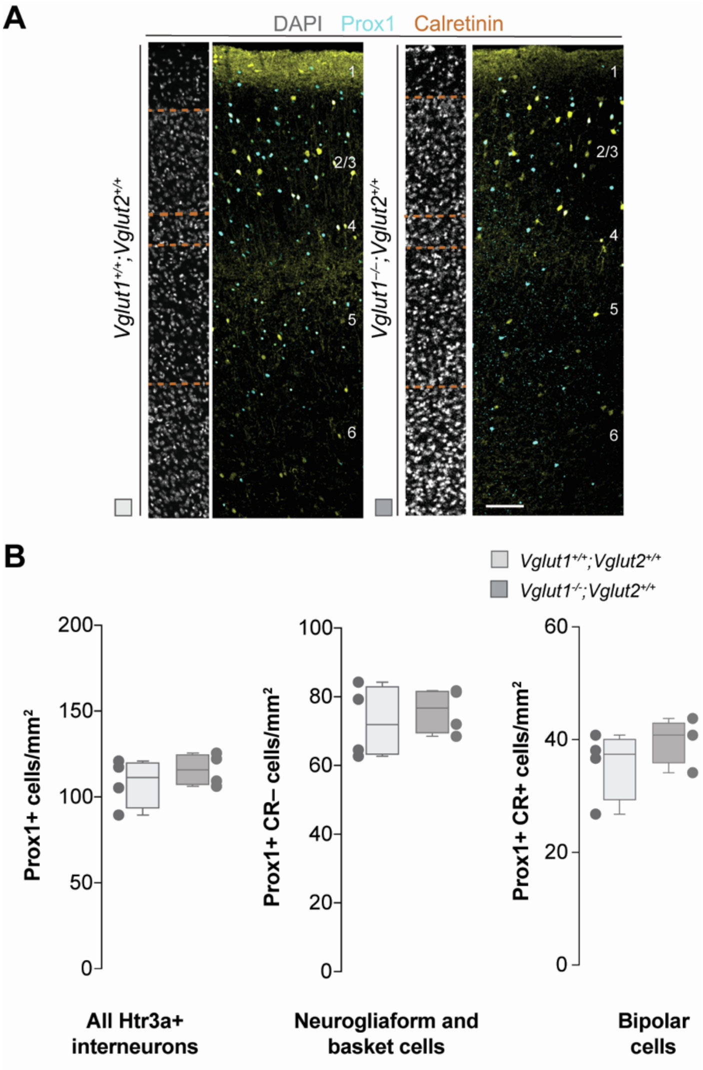

Figure S4. Removal of Vglut1 is not sufficient to alter the survival of Htr3a+ interneurons related to Figure 5

(A) Coronal sections through the primary motor cortex of *Vglut1*$^{+/+}$;*Vglut2*$^{+/+}$ mice ipsilaterally injected with Cre-expressing virus (left) and of the contralateral cortex of *Vglut1*$^{-/-}$;*Vglut2*$^{fl/fl}$ mice injected with Cre-expressing virus (right) at P21 following immunohistochemistry against Prox1 (cyan) and calretinin (yellow). DAPI is shown for counterstaining (grey).

(B) Quantification of the density of all Htr3a+ interneurons (Prox1+), neurogliaform and basket cells (Prox1+ and CR-) and bipolar cells (Prox1+ and CR+) in *Vglut1*$^{+/+}$;*Vglut2*$^{+/+}$ injected with Cre-expressing virus (light grey box plots, n = 4 mice) and *Vglut1*$^{-/-}$;*Vglut2*$^{fl/fl}$ mice injected with Cre-expressing virus at the contralateral (dark grey box plots, n = 4 mice) at P21. Prox1+; two-tailed unpaired Student's t-test, p = 0.41. Prox1+ CR-; two-tailed unpaired Student's t-test, p = 0.63. Prox1+ and CR+; two-tailed unpaired Student's t-test, p = 0.29.

Data in panel B are shown as boxplots (median, middle dash), lower and upper quartiles (box borders), and minimum and maximum (whiskers) and the adjacent data points indicate the average cell density in each animal. Scale bar, 100 μm.

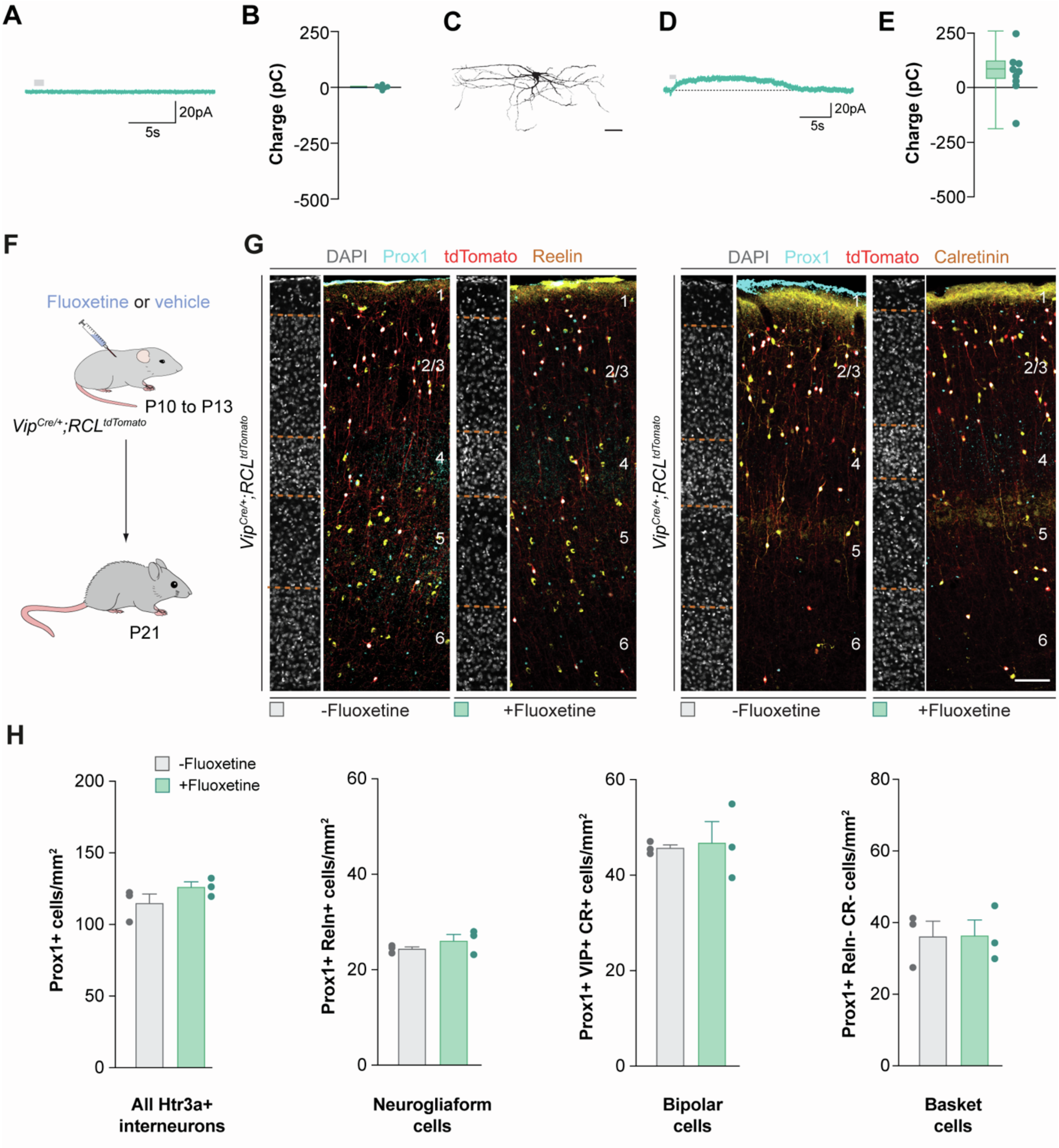

Figure S5. Neurogliaform cells do not depolarize in response to serotonin, and changes in serotonin levels beyond the normal period of cell death do not affect the survival of bipolar cells related to Figure 6

(A) Example of voltage-clamp traces of L2/3 GFP+ cells in response to aCSF puffs (grey bars).

(B) Quantification of cell charge of L2/3 GFP+ (green box plot, $n = 4$ cells).

(C) Typical morphology of reconstructed neurogliaform cell.

(D) Example of voltage-clamp traces of neurogliaform in response to serotonin puffs (grey bars).

(E) Quantification of cell charge of L1 neurogliaform cell (green box plot, $n = 9$ cells).

(F) Schematic of experimental design.

(G) Coronal sections through the primary somatosensory cortex of $Vip^{Cre/+};RCL^{tdTomato}$ mice at P21 injected vehicle or fluoxetine treatment immunostained for Prox1 (cyan), tdTomato (red) and reelin (yellow, left) or calretinin (yellow, right). DAPI is shown for counterstaining (grey).

(H) Quantification of the density of all Htr3a+ interneurons (Prox1+), neurogliaform (Prox1+ and Reln+), bipolar cells (Prox1+, VIP+ and CR+) and basket cells (Prox1+ Reln- and CR-) in control (grey bars, $n = 3$ mice) and fluoxetine injected mice (green box bars, $n = 3$ mice) at P21. Prox1+; two-tailed unpaired Student's t-test, $p = 0.21$. Prox1+ and Reln+; two-tailed unpaired Student's t-test, $p = 0.30$. Prox1+, VIP+ and CR+: two-tailed unpaired Student's t-test: $p = 0.43$. Prox1+, CR- and Reln-; two-tailed unpaired Student's t-test, $p = 0.97$.

Data in panels B and E are shown as boxplots (median, middle dash), lower and upper quartiles (box borders), and minimum and maximum (whiskers), and the adjacent data points indicate the cell charge for each cell. Data in panel H are shown as bar graphs + SEM, and the adjacent data points indicate the average cell density in each animal.

Scale bar, 100 μm.

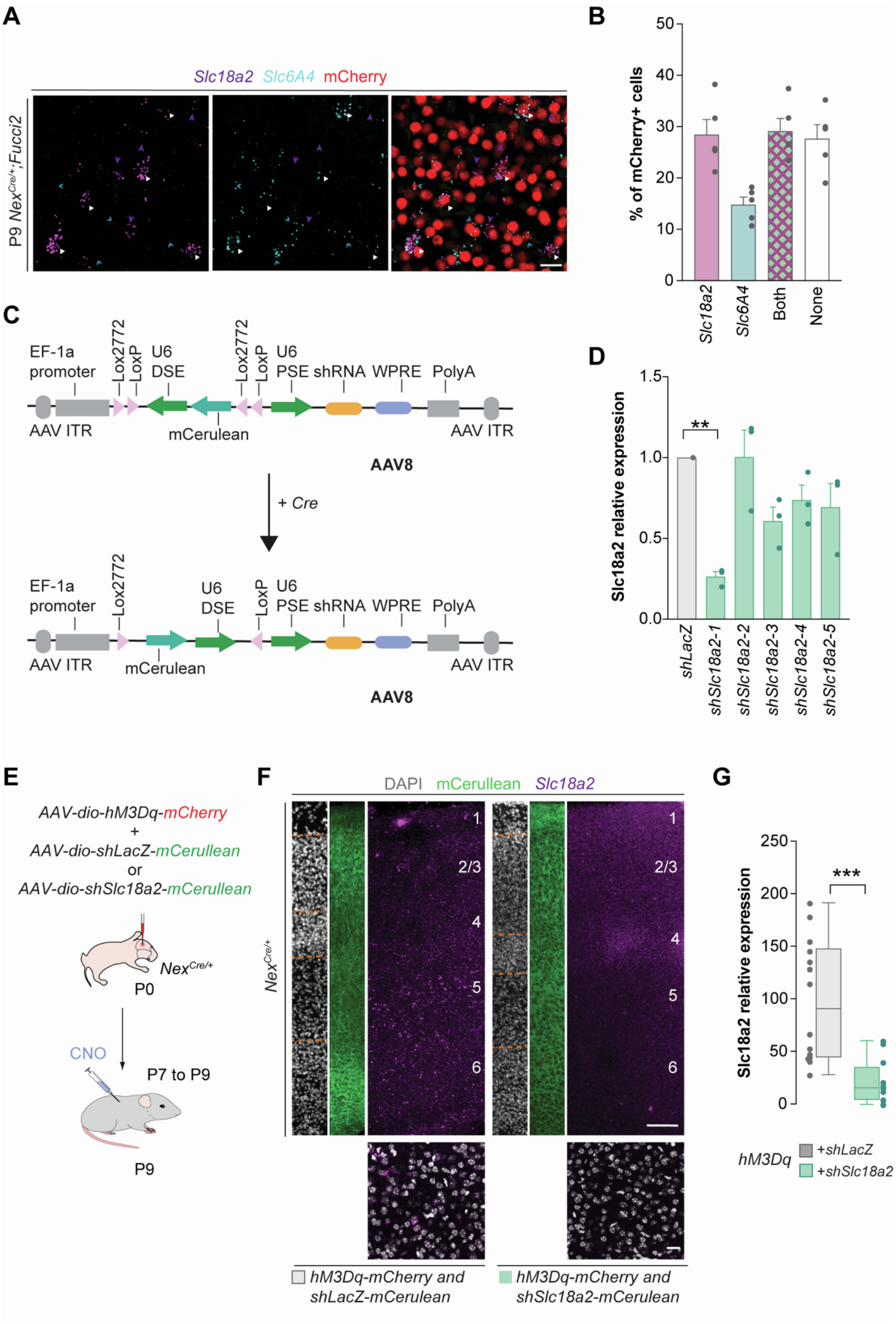

Figure S6. Validation of shSlc18a2-mCerullean construct related to Figure 7

(A) Coronal sections through the infragranular layers of the neocortex of *Nex*$^{Cre/+}$; *Fucci2* mice at P9 immunostained for mCherry (red), and RNAscope for *Slc18a2* (magenta) and *Slc6A4* (cyan). White arrowheads

are examples of pyramidal cells expressing both *Slc18a2* and *Slc6a4*, magenta arrowheads for *Slc18a2* only and cyan arrowheads for *Slc6a4* only.

(B) Quantification of mCherry+ cells expressing *Slc18a2* (magenta bar, n = 5 mice), *Slc6A4* (cyan bar, n = 5 mice), both *Slc18a2* and *Slc6a4* (checkered bar, n = 5 mice) or none of these genes (white bar, n = 5 mice).

(C) Schematic of plasmid design.

(D) Quantification of *Slc18a2* relative expression in vitro using qPCR with control (shLacZ, grey bar, n = 3 biological replicates) and sh18a2 constructs 1 to 5 (green bars, n = 3 biological replicates). *shSlc18a2-1*: one-way ANOVA with Dunnett's multiple comparison, **p = 0.0015; *shSlc18a2-2*: one-way ANOVA with Dunnett's multiple comparison, p = 0.99; *shSlc18a2-3*: one-way ANOVA with Dunnett's multiple comparison, p = 0.08; *shSlc18a2-4*: one-way ANOVA with Dunnett's multiple comparison, p = 0.32; *shSlc18a2-5*: one-way ANOVA with Dunnett's multiple comparison, p = 0.21. *shSlc18a2-1* was selected for in vivo experiments.

(E) Schematic of experimental design.

(F) Coronal sections through the primary somatosensory cortex of *Nex*$^{Cre/+}$ mice at P9 injected with *hM3Dq-mCherry* and *shLacZ-mCerulean* (left) or *shSlc18a2-mCerrulean* (right) virus followed by CNO treatment immunostained for mCerulean (green), and RNAscope for *Slc18a2* (magenta). DAPI is shown for counterstaining (grey). The insert below is a higher magnification of L5 of the RNAscope for *Slc18a2* (magenta) and DAPI counterstain.

(G) Quantification of the relative expression of *Slc18a2* of L5 and 6 with control (shLacZ, grey box plot, n =14 images) and sh18a2 constructs (green box plot, n =12 images). Mann Whitney test, ***p < 0.0001.

Data in panels B and D are shown as bar graphs ± SEM. The adjacent data points indicate the average cell density in each animal (B) and average expression from each biological replicate. Data in panel G are shown as boxplots (median, middle dash), lower and upper quartiles (box borders), and minimum and maximum (whiskers). The adjacent data points indicate the number of images.

Scale bars, 20 μm (A) and 100 μm (F).

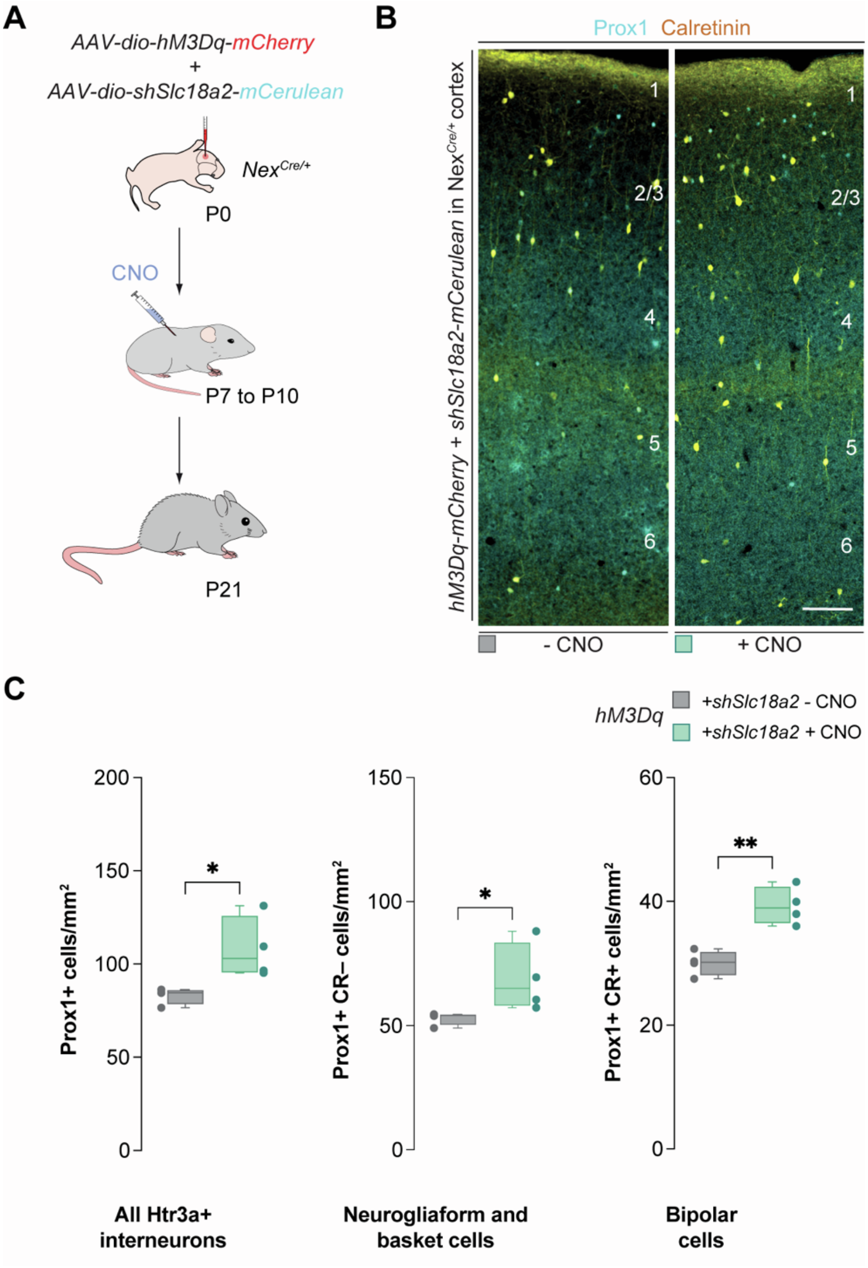

Figure S7. Knockdown of Slc18a2 alone is not sufficient to prevent the cell death of Htr3a+ interneurons related to Figure 7

(A) Schematic of experimental design.

(B) Coronal sections through the primary somatosensory cortex of *Nex*$^{Cre/+}$ mice at P21 injected with *hM3Dq-mCherry* and *shSlc18a2-mCerrulean* virus followed by vehicle (left) or CNO (right) treatment immunostained for Prox1 (cyan), and calretinin (yellow).

(C) Quantification of the density of all Htr3a+ interneurons (Prox1+), neurogliaform and basket cells (Prox1+ and CR-) and bipolar cells (Prox1+ and CR+) in *shSlc18a2-mCerrulean* mice followed by vehicle (grey box plots, $n = 4$ mice) or CNO treatment (green box plots, $n = 4$ mice) at P21. All mice were injected with the *hM3Dq-mCherry* virus. Prox1+; two-tailed unpaired Student's t-test, $*p = 0.027$. Prox1+ CR-; Mann-Whitney, $*p = 0.029$. Prox1+ and CR+; two-tailed unpaired Student's t-test, $**p = 0.0023$.

Data in panel C are shown as boxplots (median, middle dash), lower and upper quartiles (box borders), and minimum and maximum (whiskers) and the adjacent data points indicate the average cell density in each animal. Scale bar, 100 μm.

Table S1. Summary of data and statistical analyses, related to Figures 1-7 and Figures S1-S7

**FIGURE 1**	Measurement	Values	N	Statistical	P value
Figure 1B	CGE interneuron cell densities (mean ± SEM)	**Prox1+**: Control Htr3a-Cre: 128.93891 ± 9.7075; Htr3aCreBaxBak: 170.999 ± 7.6148	[brains] Control Htr3a-Cre, n = 5, Htr3aCreBaxBak, n = 4	2-tailed unpaired Student's t-test	$p = 0.0138$ (*)
		**Prox1+ Re+**: Control Htr3a-Cre: 30.018342 ± 2.4084662; Htr3aCreBaxBak: 39.798868 ± 2.812634	[brains] Control Htr3a-Cre, n = 5, Htr3aCreBaxBak, n = 4	2-tailed unpaired Student's t-test	$p = 0.0327$ (*)
		**Prox1+ CR+**: Control Htr3a-Cre: 33.222357 ± 2.5819982; Htr3aCreBaxBak:44.76981 ± 3.1535676	[brains] Control Htr3a-Cre, n = 5, Htr3aCreBaxBak, n = 4	2-tailed unpaired Student's t-test	$p = 0.0309$ (*)
		**Prox1+ CR-Re-**: Control Htr3a-Cre: 65.698208 ± 6.7524041; Htr3aCreBaxBak: 86.430708 ± 3.031828	[brains] Control Htr3a-Cre, n = 5, Htr3aCreBaxBak, n = 4	2-tailed unpaired Student's t-test	$p = 0.0327$ (*)
**FIGURE 2**	Measurement	Values	N	Statistical	P value
Figure 2B	CGE interneuron cell densities (mean ± SEM)	**Prox1+**: hM3Dq-CNO: 63.134157 ±2.855373; hM3Dq+CNO: 77.079124 ± 2.998725	[brains] n = 6 for both groups	2-tailed unpaired Student's t-test	$p = 0.007$ (**)
		**Prox1+ Re+**: hM3Dq-CNO: 18.0878276 ± 1.22917991; hM3Dq+CNO: 25.807598± 2.9800354	[brains] n = 6 for both groups	2-tailed unpaired Student's t-test	$p = 0.0377$ (*)
		**Prox1+ CR+**: hM3Dq-CNO: 23.053624 ±1.6097517; hM3Dq+CNO: 31.14537 ± 1.8146412	[brains] n = 6 for both groups	2-tailed unpaired Student's t-test	$p = 0.0121$ (*)
		**Prox1+ CR-Re-**: hM3Dq-CNO: 27.4930811 ± 3.02664295; hM3Dq+CNO: 37.6835104 ± 2.75023481	[brains] n = 6 for both groups	2-tailed unpaired Student's t-test	$p = 0.0319$ (*)
Figure 2C	CGE interneuron cell densities (mean ± SEM)	**Prox1+**: hM4Di-CNO: 68.915974 ±5.2489537; hM4Di+CNO: 55.0325501 ± 1.96627922	[brains] hM4Di -CNO, n = 4, hM4Di+CNO n = 5	2-tailed unpaired Student's t-test	$p = 0.0259$ (*)
		**Prox1+ Re+**: hM4Di-CNO: 24.9941011 ±1.1484826; hM4Di+CNO: 17.5705351 ± 1.39640632	[brains] hM4Di -CNO, n = 4, hM4Di+CNO n = 5	2-tailed unpaired Student's t-test	$p = 0.0055$ (**)
		**Prox1+ CR+**: hM4Di-CNO: 30.0366435 ± 2.11197677; hM4Di+CNO: 21.4524183 ± 1.13356151	[brains] hM4Di -CNO, n = 4, hM4Di+CNO n = 5	2-tailed unpaired Student's t-test	$p = 0.0047$ (**)
		**Prox1+ CR-Re-**: hM4Di-CNO: 20.8646658 ± 0.74526041; hM4Di+CNO: 12.2050725 ± 0.83210095	[brains] hM4Di -CNO, n = 4, hM4Di+CNO n = 5	2-tailed unpaired Student's t-test	$p = 0.0001$ (***)
**FIGURE 3**	Measurement	Values	N	Statistical	P value
Figure 3B	CGE interneuron cell densities (mean ± SEM)	**Prox1+**: Control Nex-Cre:125.955086 ±4.9773057; NexCreBaxBak: 171.70553 ±8.352957	[brains] n = 7 for both groups	2-tailed unpaired Student's t-test	$p = 0.0005$ (***)
		**Prox1+ Re+**: Control Nex-Cre: 29.690536 ± 1.9780853; NexCreBaxBak: 38.699654 ± 2.2600626	[brains] n = 7 for both groups	2-tailed unpaired Student's t-test	$p = 0.0113$ (*)
		**Prox1+ CR+**: Control Nex-Cre: 29.707267 ± 1.3975519; NexCreBaxBak: 42.090428 ± 3.091244	[brains] n = 7 for both groups	2-tailed unpaired Student's t-test	$p = 0.0036$ (**)
		**Prox1+ CR-Re-**: Control Nex-Cre: 66.553056 ± 3.38164542; NexCreBaxBak: 90.979209 ± 4.2374106	[brains] n = 7 for both groups	2-tailed unpaired Student's t-test	$p = 0.002$ (**)
**FIGURE 4**	Measurement	Values	N	Statistical	P value
Figure 4C	CGE interneuron cell densities (mean ± SEM)	**Prox1+**: hM3Dq-CNO: 140.22185 ±6.1886976; hM3Dq+CNO: 168.24575 ±9.2067176	[brains] n = 7 for both groups	2-tailed unpaired Student's t-test	$p = 0.0266$ (*)
		**Prox1+ Re+**: hM3Dq-CNO: 30.5171011 ± 1.28699855; hM3Dq+CNO: 40.5280454 ± 1.0966376	[brains] n = 7 for both groups	2-tailed unpaired Student's t-test	$p < 0.0001$ (***)
		**Prox1+ CR+**: hM3Dq-CNO: 41,618613 ± 1.9027866; hM3Dq+CNO: 51.031663 ± 1.7405535	[brains] n = 7 for both groups	2-tailed unpaired Student's t-test	$p = 0.0033$ (**)
		**Prox1+ CR-Re-**: hM3Dq-CNO: 42.1597898 ± 2.2139241; hM3Dq+CNO: 60.3700123 ± 3.03048226	[brains] n = 7 for both groups	2-tailed unpaired Student's t-test	$p = 0.0004$ (***)

Figure 4D	CGE interneuron cell densities (mean ± SEM)	**Prox1+**: hM4Di-CNO: 175.759708 ± 6.2889318; hM4Di+CNO:153.962902 ± 6.37779553	[brains] n = 6 for both groups	2-tailed unpaired Student's t-test	*p* = 0.0352 (*)
		**Prox1+ Re+**: hM4Di-CNO: 34.9688292 ± 1.89106344; hM4Di+CNO: 26.9684615 ± 1.43152338	[brains] hM4Di -CNO, n = 5, hM4Di+CNO n = 6	2-tailed unpaired Student's t-test	*p* = 0.0074 (**)
		**Prox1+ CR+**: hM4Di-CNO: 50.7213356 ± 1.6083140; hM4Di+CNO: 40.5899939 ± 3.37004675	[brains] n = 6 for both groups	2-tailed unpaired Student's t-test	*p* = 0.0218 (*)
		**Prox1+ CR-Re-**: hM4Di-CNO: 76.786516 ± 4.023283; hM4Di+CNO: 58.709725 ± 5.5024846	[brains] n = 6 for both groups	2-tailed unpaired Student's t-test	*p* = 0.0242 (*)
**FIGURE 5**	Measurement	Values	N	Statistical	P value
Figure 5D	CGE interneuron cell densities (mean ± SEM)	**Prox1+**: Control: 108.282796 ± 7.10449118; VG1/VG2 KO: 90.2908768 ± 5.94786571	[brains] n = 4 for both groups	2-tailed unpaired Student's t-test	*p* = 0.1002 (NS)
		**Prox1+ CR-**: Control: 72.6899503 ± 5.35347727; VG1/VG2 KO: 54.7620478 ± 2.43474891	[brains] n = 4 for both groups	2-tailed unpaired Student's t-test	*p* = 0.026 (*)
		**Prox1+ CR+**: Control: 35.5928459 ± 3.05633804; VG1/VG2 KO: 35.528829 ± 3.99582597	[brains] n = 4 for both groups	2-tailed unpaired Student's t-test	*p* = 0.9903 (NS)
**FIGURE 6**	Measurement	Values	N	Statistical	P value
Figure 6C	Charge (mean ± SEM)	L2/3 Bipolar cells: 76704.81 ± 29550.95	[cells] n = 7		
		L2/3 Basket cells: -229518 ± 51407.16	[cells] n = 7		
Figure 6D	CGE interneuron cell densities (mean ± SEM)	**Prox1+**: Vehicle: 140.167528 ± 5.97875389; Fluoxetine: 146.833266 ± 10.612415	[brains] n = 4 for both groups	2-tailed unpaired Student's t-test	*p* = 0.6093 (NS)
		**Prox1+ Re+**: Vehicle: 145.348521 ± 5.79093263; Fluoxetine: 151.235858 ± 18.8224061	[brains] n = 4 for both groups	2-tailed unpaired Student's t-test	*p* = 0.9834 (NS)
		**Prox1+ VIP+ CR+**: Vehicle: 36.6817236 ± 1.65394208; Fluoxetine: 49.087944 ± 3.12794821	[brains] n = 4 for both groups	2-tailed unpaired Student's t-test	*p* = 0.0127 (*)
		**Prox1+ CR-Re-**: Vehicle: 55.493346 ± 8.7814519; Fluoxetine: 41.861755 ± 5.0760216	[brains] n = 4 for both groups	2-tailed unpaired Student's t-test	*p* = 0.2276 (NS)
**FIGURE 7**	Measurement	Values	N	Statistical	P value
Figure 7C	CGE interneuron cell densities (mean ± SEM)	**Prox1+**: shLacZ: 117.845708 ± 7.74735528; shVmat2: 111.997696 ± 8.354898	[brains] n = 4 for both groups	2-tailed unpaired Student's t-test	*p* = 0.5347 (NS)
		**Prox1+ CR-**: shLacZ: 72.9697875 ± 7.84245371; shVmat2: 71.6411254 ± 6.92400581	[brains] n = 4 for both groups	2-tailed unpaired Student's t-test	*p* = 0.8547 (NS)
		**Prox1+ CR+**: shLacZ: 44.8759186 ± 1.32738498; shVmat2: 39.2727969 ± 1.52053381	[brains] n = 4 for both groups	2-tailed unpaired Student's t-test	*p* = 0.0343 (*)
**FIGURE S1**	Measurement	Values	N	Statistical	P value
Figure S1A	Proportion of cells (%)	**Expected results**: NGF/SBC: 30%; BP: 27%; BC: 43%; **Observed results**: NGF/SBC: 23%; BP: 25%; BC 52%	[brains] Control Htr3a-Cre, n = 5, Htr3aCreBaxBak, n = 4	Chi-quare	*p* = 0.16 (NS)
**FIGURE S2**	Measurement	Values	N	Statistical	P value
Figure S2C	CGE interneuron cell densities (mean ± SEM)	**Prox1+**: hM3Dq-CNO: 66.284849 ± 1.5028887; hM3Dq+CNO: 60.064579 ±3.047367	[brains] n = 4 for both groups	2-tailed unpaired Student's t-test	*p* = 0.1169 (NS)
		**Prox1+ Re+**: hM3Dq-CNO: 26.8957068 ± 3.53002062; hM3Dq+CNO: 25.1264708 ± 4.90953428	[brains] n = 4 for both groups	2-tailed unpaired Student's t-test	*p* = 0.6692 (NS)
		**Prox1+ CR+**: hM3Dq-CNO: 26.3253613 ± 2.43294121; hM3Dq+CNO: 23.5634017 ± 3.39731554	[brains] n = 4 for both groups	2-tailed unpaired Student's t-test	*p* = 0.5532 (NS)
		**Prox1+ CR-Re-**: hM3Dq-CNO: 19.42593 ± 2.509301; hM3Dq+CNO: 18.053787 ± 0.9675375	[brains] n = 4 for both groups	2-tailed unpaired Student's t-test	*p* = 0.5436 (NS)
**FIGURE S3**	Measurement	Values	N	Statistical	P value
		**Prox1+**: hM3Dq-CNO: 113.063459 ±4.09645928; hM3Dq+CNO: 118.699518 ± 6.80751163	[brains] hM3Dq -CNO, n = 3, hM3Dq+CNO n = 4	2-tailed unpaired Student's t-test	*p* = 0.5480 (NS)

Figure S3C	CGE interneuron cell densities (mean ± SEM)	**Prox1+ Re+**: hM3Dq-CNO: 23.5569756 ± 1.62279407; hM3Dq+CNO: 19.612295 ± 1.10857406	[brains] hM3Dq -CNO, n = 3, hM3Dq+CNO n = 4	2-tailed unpaired Student's t-test	*p* = 0.0910 (NS)
		**Prox1+ CR+**: hM3Dq-CNO: 41.360781 ± 2.99063331; hM3Dq+CNO: 40.424176 ± 2.46681341	[brains] hM3Dq -CNO, n = 3, hM3Dq+CNO n = 4	2-tailed unpaired Student's t-test	*p* = 0.8172 (NS)
		**Prox1+ CR-Re-**: hM3Dq-CNO: 64.6671703 ± 7.73374349; hM3Dq+CNO: 73.0327285 ± 7.24395448	[brains] hM3Dq -CNO, n = 3, hM3Dq+CNO n = 4	2-tailed unpaired Student's t-test	*p* = 0.4712 (NS)
Figure S3D	CGE interneuron cell densities (mean ± SEM)	**Prox1+**: hM4Di-CNO: 127.116395 ± 1.37724603; hM4Di+CNO: 123.338106 ± 2.02843504	[brains] n = 3 for both groups	2-tailed unpaired Student's t-test	*p* = 0.1982 (NS)
		**Prox1+ Re+**: hM4Di-CNO: 31.0610279 ± 3.09209328; hM4Di+CNO: 30.3948183 ± 1.20604652	[brains] n = 3 for both groups	2-tailed unpaired Student's t-test	*p* = 0.8507 (NS)
		**Prox1+ CR+**: hM4Di-CNO: 33.2822563 ± 0.71278089; hM4Di+CNO: 31.0766782 ± 0.1240153	[brains] n = 3 for both groups	2-tailed unpaired Student's t-test	*p* = 0.1240 (NS)
		**Prox1+ CR-Re-**: hM4Di-CNO: 76.1695 ± 3.079432; hM4Di+CNO: 69.42585 ± 1.046768	[brains] n = 3 for both groups	2-tailed unpaired Student's t-test	*p* = 0.1068 (NS)
**Figure S4**	Measurement	Values	N	Statistical	P value
Figure S4B	CGE interneuron cell densities (mean ± SEM)	**Prox1+**: *Vglut1+/+;Vglut2+/+* : 108.282796 ± 7.10449118 *Vglut1-/-;Vglut2+/+* : 115.77086 ± 4.7356754	[brains] n = 4 for all groups	2-tailed unpaired Student's t-test	p = 0.4142 (NS)
		**Prox1+ CR-**: Vglut1+/+;Vglut2+/+: 72.6899503 ± 5.35347727; Vglut1-/-;Vglut2+/+: 75.899582 ± 3.3346367	[brains] n = 4 for all groups	2-tailed unpaired Student's t-test	p = 0.6290 (NS)
		**Prox1+ CR+**: Vglut1+/+;Vglut2+/+: 35.5928459 ± 3.05633804; Vglut1-/-;Vglut+/+: 39.871273 ± 2.0342391	[brains] n = 4 for all groups	2-tailed unpaired Student's t-test	p = 0.2881 (NS)
**FIGURE S5**	Measurement	Values	N	Statistical	P value
Figure S5A	Charge (mean ± SEM)	GFP+ cells: 971.4932 ± 4764.166	[cells] n = 6		
Figure S5B	Charge (mean ± SEM)	L1 NGF: 62304.41 ± 36233.81	[cells] n = 9		
Figure S5H	CGE interneuron cell densities (mean ± SEM)	**Prox1+**: Vehicle: 114.782539 ± 6.54602903; Fluoxetine: 126.079933 ±3.68675634	[brains] n = 3 for both groups	2-tailed unpaired Student's t-test	*p* = 0.2071 (NS)
		**Prox1+ Re+**: Vehicle: 24.3711865 ±0.40454126; Fluoxetine: 26.0408337 ±1.32701948	[brains] n = 3 for both groups	2-tailed unpaired Student's t-test	*p* = 0.2951 (NS)
		**Prox1+ VIP+ CR+**: Vehicle: 49.0143131 ± 0.79261738; Fluoxetine: 50.1843306 ± 4.20798866	[brains] n = 3 for both groups	2-tailed unpaired Student's t-test	*p* = 0.4295 (NS)
		**Prox1+ CR-Re-**: Vehicle: 36.062905 ± 4.33895612; Fluoxetine: 36.3178117 ± 4.38526967	[brains] n = 3 for both groups	2-tailed unpaired Student's t-test	*p* = 0.9694 (NS)
**FIGURE S6**	Measurement	Values	N	Statistical	P value
Figure S6B	% of mCherry+ cells (mean ± SEM)	Slc18a+ only cells: 28.42467 ± 2.9634; Slc6A4+ only cells: 13.81044 ± 1.4421; Slc18a+ and Slc6A4+ cells: 29.11470 ± 2.46729; Slc18a0- and Slc6A4- cells: 27.65019 ± 2.7433	[brains] n = 5 for all groups		
Figure S6D	Slc18a2 relative expression (mean ± SEM)	shslc18a2-1: 026333 ± 0.03179797; shslc18a2-2: 1.003333 ± 0.16676664; shslc18a2-3: 0.6066667 ± 0.08819171; shslc18a2-4: 0.7366667 ± 0.09333333; shslc18a2-5: 06933333 ± 0.14678026	[experiments] n = 3 for all groups	1-way Anova	*p* = 0.0015 (**) shLacZ vs *shSlc18a2-1*
Figure S6G	Slc18a2 relative expression (mean ± SEM)	shLacZ: 100 ± 15.3447603, shslc18a2: 22.4988438 ± 6.0643048	[images] shLacZ = 14, shslc18a1 = 12	Mann Whitney	*p* < 0.0001 (***)
**FIGURE S7**	Measurement	Values	N	Statistical	P value
Figure S7C	CGE interneuron cell densities (mean ± SEM)	**Prox1+:** -CNO: 83.01319 ± 2.18582;+CNO: 111.997696 ± 8.354898	[brains] n = 4 for both groups	2-tailed unpaired Student's t-test	*p* = 0.027 (*)
		**Prox1+ CR-** : -CNO: 52.97631 ± 1.30069; +CNO: 71.6411254 ± 6.92400581	[brains] n = 4 for both groups	Mann Whitney	*p* = 0.0286 (*)
		**Prox1+ CR+** : -CNO: 30.03689 ± 0.9959; +CNO: 39.2727969 ± 1.52053381	[brains] n = 4 for both groups	2-tailed unpaired Student's t-test	*p* = 0.0023 (**)
